# Patient-centered burdens and economic outcomes among patients who are veterans, people with intellectual and developmental disabilities, and people living in rural areas and their caregivers: a scoping review

**DOI:** 10.3389/fpubh.2026.1749239

**Published:** 2026-04-21

**Authors:** Naomi Buell, Ella Shenkar, Sydney Kirsch, Diana Poehler, Olga Khavjou, Kristen Giombi

**Affiliations:** RTI International, Durham, NC, United States

**Keywords:** caregiving - informal, intellectual and developmental disabilities, patient-centered burdens and economic outcomes, rural health, veteran health

## Abstract

**Background:**

This review synthesized literature on patient-centered burdens and economic outcomes (PCBEOs) for three populations that may be underrepresented in the literature—military veterans, people with intellectual and developmental disabilities (IDD), and individuals living in rural areas—to better understand how PCBEOs are captured for these populations.

**Methods:**

We searched PubMed, CINAHL, EconLit, Web of Science, and APA PsycInfo (January 2015–April 2025) for U.S.-based studies of PCBEOs due to medical reasons for veterans, people with IDD, or people living in rural areas or their caregivers. We categorized PCBEOs into direct medical costs, direct non-medical costs, indirect impacts, and intangible burdens. We examined population-specific rationales for studying these outcomes reported in the studies.

**Results:**

Of 1,549 identified records, 126 met inclusion criteria. Intangible burdens were the most frequently reported PCBEOs (*n* = 84, 67%), while direct medical (*n* = 47, 37%) and non-medical costs (*n* = 28, 22%) were least commonly assessed. Patterns in PCBEOs varied across populations: studies of veterans focused narrowly on intangible burdens (*n* = 23, 85% of veteran studies), specifically composite measures of caregiver burden (*n* = 17 studies, 63% of veteran studies on intangible burdens). Studies on people with IDD and rural populations more often examined multiple PCBEO categories, including indirect impacts (IDD *n* = 33, 58%; rural *n* = 22, 47%) such as unpaid caregiving time; direct medical costs (IDD *n* = 25, 44%; rural *n* = 19, 40%) such as out-of-pocket medical expenses, and direct non-medical costs (IDD *n* = 14, 25%; rural *n* = 14, 30%) such as travel costs. Across all groups, few studies assessed the full spectrum of PCBEOs. Most studies (*n* = 96, 76%) provided research justifications tailored to their study population, though this varied across groups—about half of veteran studies (*n* = 13, 48%) included justifications, compared with 84% (*n* = 48) for IDD and 70% (*n* = 33) for rural populations.

**Conclusion:**

The frequent reporting of intangible burdens across all populations highlights substantial emotional and psychological strains faced by these groups. Considerable variability in PCBEOs examined across populations reveals gaps in comprehensive assessment of the full range of PCBEOs that each group experienced. These findings underscore the need for systematic data collection to more fully capture the range of burdens for these populations.

## 1 Introduction

Patients and caregivers in the United States experience many challenges affording and accessing healthcare, with 44% of adults reporting difficulty affording healthcare costs (1). These challenges vary across populations, with many patient populations facing higher healthcare costs and increased barriers to care (1–3). Military veterans, people with intellectual and developmental disabilities (IDD), and people living in rural areas face systemic barriers to inclusion in health systems that often intensify financial and non-financial burdens (4–8). These groups were intentionally selected because they represent diverse structural and clinical challenges, ranging from service complexity to geographic isolation, allowing us to compare how patient-centered burdens and economic outcomes (PCBEOs) manifest across contexts and how their study is justified in the literature (4, 9–11).

Veterans’ healthcare experiences often include many barriers, including complex navigation between U.S. Department of Veterans Affairs (VA) and civilian systems, long travel distances to VA facilities, and delays in accessing specialized care (4, 5, 12). These factors increase time costs, transportation burdens, and stress for veterans and their caregivers (4, 5, 12). In addition, the high prevalence of military service-related conditions can drive greater healthcare utilization, out-of-pocket expenses, lost work productivity, and caregiving demands (13). People with IDD often encounter significant barriers in healthcare, including limited access to trained providers, communication challenges, and fragmented care systems (6, 7, 14). These challenges, coupled with higher rates of chronic conditions and substantial caregiving demands, contribute to increased time, financial, and emotional burdens in this population and their caregivers (15). People living in rural areas experience barriers related to limited provider availability, geographic isolation, and lack of economic resources, leading to higher time costs, travel burdens, and delayed care (8).

**Figure 1:**
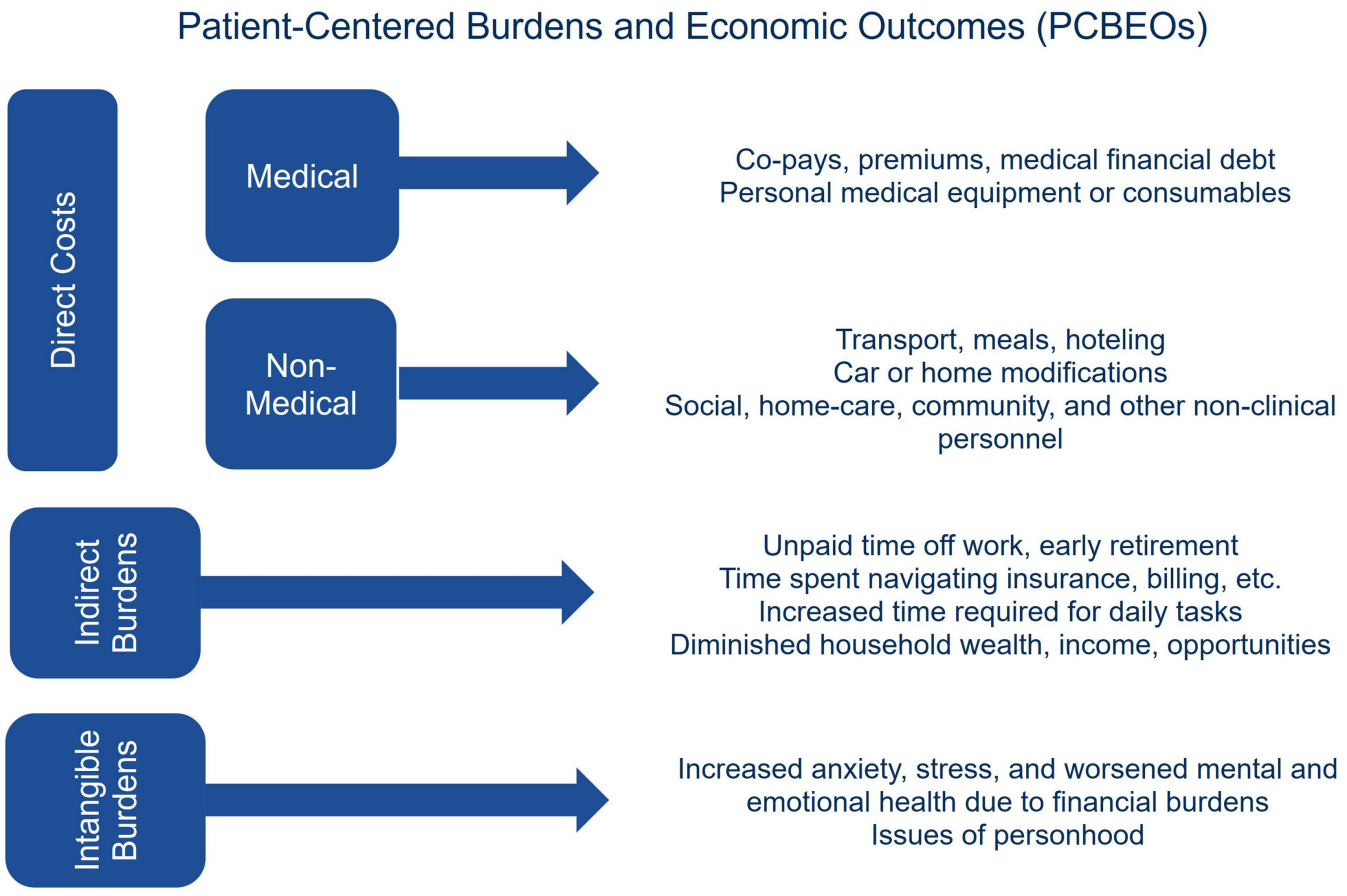
Categorization of patient-centered burdens and economic outcomes based on the Patient-Centered Outcomes Research Institute PCBEO landscape (16). Patient-Centered Outcomes Research Institute. Patient-Centered Burdens and Economic Outcomes Landscape. September 2024., Reprinted with permission from the Patient-Centered Outcomes Research Institute. © 2025 Patient-Centered Outcomes Research Institute. All Rights Reserved., https://www.pcori.org/sites/default/files/PCORI-Patient-Centered-Economic-Outcomes-Landscape-090524.pdf#page=13.08.

These burdens are considered PCBEOs, a framework developed by the Patient-Centered Outcomes Research Institute (PCORI) to capture “the full range of clinical and patient-centered outcomes, which include potential burdens and economic impacts of the utilization of medical treatments, items, and services on different stakeholders and decision makers” (16). PCBEOs capture outcomes that extend beyond conventional monetary cost measures to encompass the financial, time, and emotional impacts experienced by patients and caregivers (see Figure 1 for categorization of PCBEOs with examples). Research on PCBEOs plays a critical role in informing policies and programs that address the financial and non-financial costs borne by patients and their caregivers. However, certain populations remain underrepresented in the literature, limiting the evidence base needed to fully understand their experiences.

This study aims to review and synthesize extant evidence on PCBEOs reported for veterans, people with IDD, and people living in rural areas—three populations that face distinct but overlapping barriers to healthcare access and may experience heightened financial and non-financial burdens. To our knowledge, this review is the first to detail the peer-reviewed evidence on the PCBEOs experienced by each of these three populations and to explicitly identify the justifications cited for this research. By examining these groups together, this study explores how the literature frames their inclusion in PCBEO research and identifies common and population-specific drivers of burden. Considering these groups jointly allows us to identify common drivers of burden that may be overlooked when each group is analyzed in isolation, while also supporting more PCBEO-specific interpretations across different settings. The current scoping review seeks to answer the following research questions (RQs):

- (RQ1) What PCBEOs are reported in PCBEO studies that include veterans, people with IDD, and people living in rural areas?
- (RQ2) Among the PCBEO studies identified in RQ1, what are the population-specific justifications or reasons that the studies reported for the need to research PCBEOs for veterans, people with IDD, and people living in rural areas?

## 2 Materials and methods

We conducted a scoping review of the literature following the Preferred Reporting Items for Systematic Reviews and Meta-Analysis (PRISMA) and Joanna Briggs Institute guidance for scoping reviews (17, 18). We registered the protocol for this scoping review at AsPredicted on April 3, 2025.

### 2.1 Search strategy and data sources

We developed a search strategy that included terms related to patients and caregivers among the three populations of interest, drawing from existing systematic reviews of veterans (19, 20), people with IDD (11, 21–24), and people living in rural areas (25–27). Additionally, terms captured PCBEOs, such as copayments, missed work, and financial burden, based on the PCORI PCBEO landscape report (16). Supplementary material A presents the detailed search strategy. We searched PubMed, CINAHL, EconLit, Web of Science, and APA PsycInfo for original research published in English from January 1, 2015, through April 8, 2025.

### 2.2 Study inclusion criteria

Supplementary material B details the study selection criteria. The population included patients in the United States from the populations of interest (veterans, people with IDD, and people living in rural areas) and/or their families or caregivers. We identified an initial list of included IDD conditions from the literature while developing the search strategy and added relevant conditions as they were identified during the review process. Supplementary material C presents the IDD conditions captured in the review. The intervention was physical, emotional, financial, or time-related burdens from a medical condition or medical care. Eligible outcomes included PCBEOs (i.e., direct medical costs, direct non-medical costs, indirect impacts, intangible burdens) reported for any population of interest and the justification for measuring the PCBEOs for veterans, people with IDD, or people living in rural areas. We excluded literature reviews, studies conducted outside the United States, studies that did not focus on a population of interest (e.g., medical providers or healthcare systems), and studies that did not report PCBEOs related to medical reasons (e.g., childcare costs unrelated to health conditions).

### 2.3 Study selection and abstraction

We conducted a two-stage review using DistillerSR (28) to abstract the study’s populations of interest (i.e., veteran, IDD, and/or rural patients and/or caregivers), sample size, medical conditions, purpose, focus, geography of data collection, data sources, PCBEOs studied, and population-specific research justifications. In the first stage, two reviewers independently screened the article title and abstract. In cases of disagreements, a third reviewer determined whether the article title and abstract met inclusion criteria. Included articles from the title and abstract review stage advanced to the second stage for full-text review.

In the second stage, one reviewer screened each article for inclusion. If the reviewer determined the article met the inclusion criteria, they abstracted relevant study characteristics and outcomes. Then, a second reviewer screened the article. If the first and second reviewers decided to include the article, the second reviewer checked the abstraction for completeness and accuracy. If the first reviewer excluded the article and the second reviewer included the article, the second reviewer abstracted the relevant information. A third reviewer determined inclusion or exclusion for disagreements. When the third reviewer determined the article met the inclusion criteria, they also checked the abstracted information.

As part of the abstraction process and in accordance with the guidelines in the PCORI PCBEO landscape (16), reviewers characterized data on PCBEOs as follows:

- Direct medical costs: Monetary costs incurred by patients, families, or caregivers for patients receiving direct medical care, such as copayments for a doctor’s appointment or out-of-pocket costs of medication.
- Direct non-medical costs: Monetary costs incurred by patients, families, or caregivers that are not directly a part of the medical care received but are necessary to obtaining that care, such as childcare costs during or transportation costs to medical appointments.
- Indirect impacts: Tangible impacts incurred by patients, families, or caregivers that do not reflect direct spending, such as time spent going to the doctor or providing unpaid caregiving or unpaid time off work.
- Intangible burdens: Burdens on patients, families, or caregivers and economic outcomes that cannot be monetized or are difficult to monetize, such as financial toxicity (i.e., distress, stress, and anxiety directly caused by out-of-pocket costs, financial debt, or lost income), delaying treatments due to costs, or hindered career advancement.

Additionally, reviewers noted when articles provided one or more population-specific justifications for their PCBEO research and categorized these population justifications into five categories: predisposing factors, enabling factors, need factors, research or data gaps, and policy factors. We based these categories on Andersen’s behavioral model, which provides a theoretical structure to understand healthcare access and use (29). Predisposing factors included justifications related to social structures of the population, such as age, education, gender/sex, marital status, and employment status. Enabling factors were justifications related to the availability of resources to the population, such as income, medical insurance, rurality and distance to the nearest medical institution, or social support or help received from relatives. Need factors were justifications related to the health needs of the group, like chronic illnesses, perceived general health status, and disability status (30). We also recorded justifications citing gaps in the data or a lack of existing evidence in the current literature on PCBEOs in the population, as well as policy-driven justifications for population-specific PCBEO research.

### 2.4 Analysis

After abstraction, we synthesized findings by summarizing the characteristics of the included studies in narrative and tabular formats. Because this is a scoping review, we did not conduct risk-of-bias assessments on included studies, quantitatively synthesize findings, or conduct strength-of-evidence assessments (31).

## 3 Results

### 3.1 Study selection and characteristics

**Figure 2:**
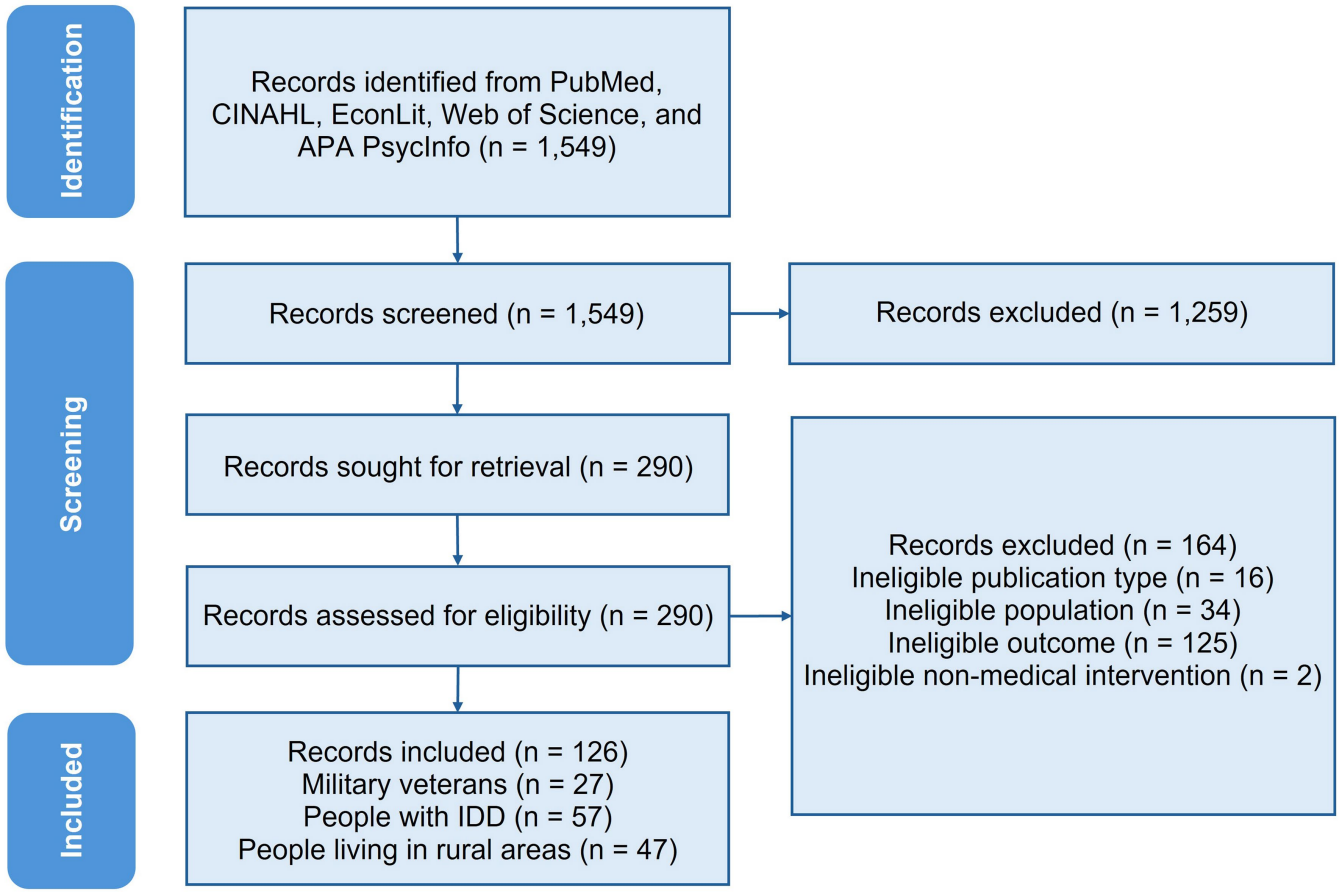
PRISMA flow diagram detailing the identification, screening, and inclusion of studies. CINAHL, Cumulative Index to Nursing and Allied Health Literature; IDD, intellectual and developmental disabilities. Because some studies include more than one population, the sum of studies by population exceeds the total.

The database search resulted in 1,549 titles and abstracts; after the two stages of screening, we included a total of 126 studies (Figure 2).

Of the 126 studies included, 27 studies focused on veterans, 57 studies focused on people with IDD, and 47 studies included people living in rural areas (because some studies included more than one population, the sum of studies by population exceeds the total). Most of the studies used quantitative data like medical records, electronic health records, claims, or survey data (*n* = 112), whereas 18 studies used qualitative data like focus groups or interviews, and 4 studies used a mix of both. Over half of studies collected data at the national level (*n* = 64) (see Supplementary material D). The most common condition that appeared in our review was autism spectrum disorder (ASD) (*n* = 36, all of these were studies of people with IDD, and one study also included people living in rural areas). This likely reflects that ASD is one of the most commonly identified developmental disabilities in the United States, and ASD diagnosis rates have increased substantially in recent decades (32, 33). Some of the ASD studies also included people with attention-deficit/hyperactivity disorder, non-ASD IDD, and other types of disabilities. Cancers were the second most common medical condition (*n* = 13, including 12 studies of rural populations and 1 study of veterans), followed by traumatic brain injury (*n* = 6, all of these were studies on veterans) (see Supplementary material C for frequency of medical conditions in each population). Detailed information on each included study is provided in Supplementary material E.

### 3.2 Synthesized findings

#### 3.2.1 RQ1: what PCBEOs are reported in PCBEO studies that include veterans, people with IDD, and people living in rural areas?

**Figure 3:**
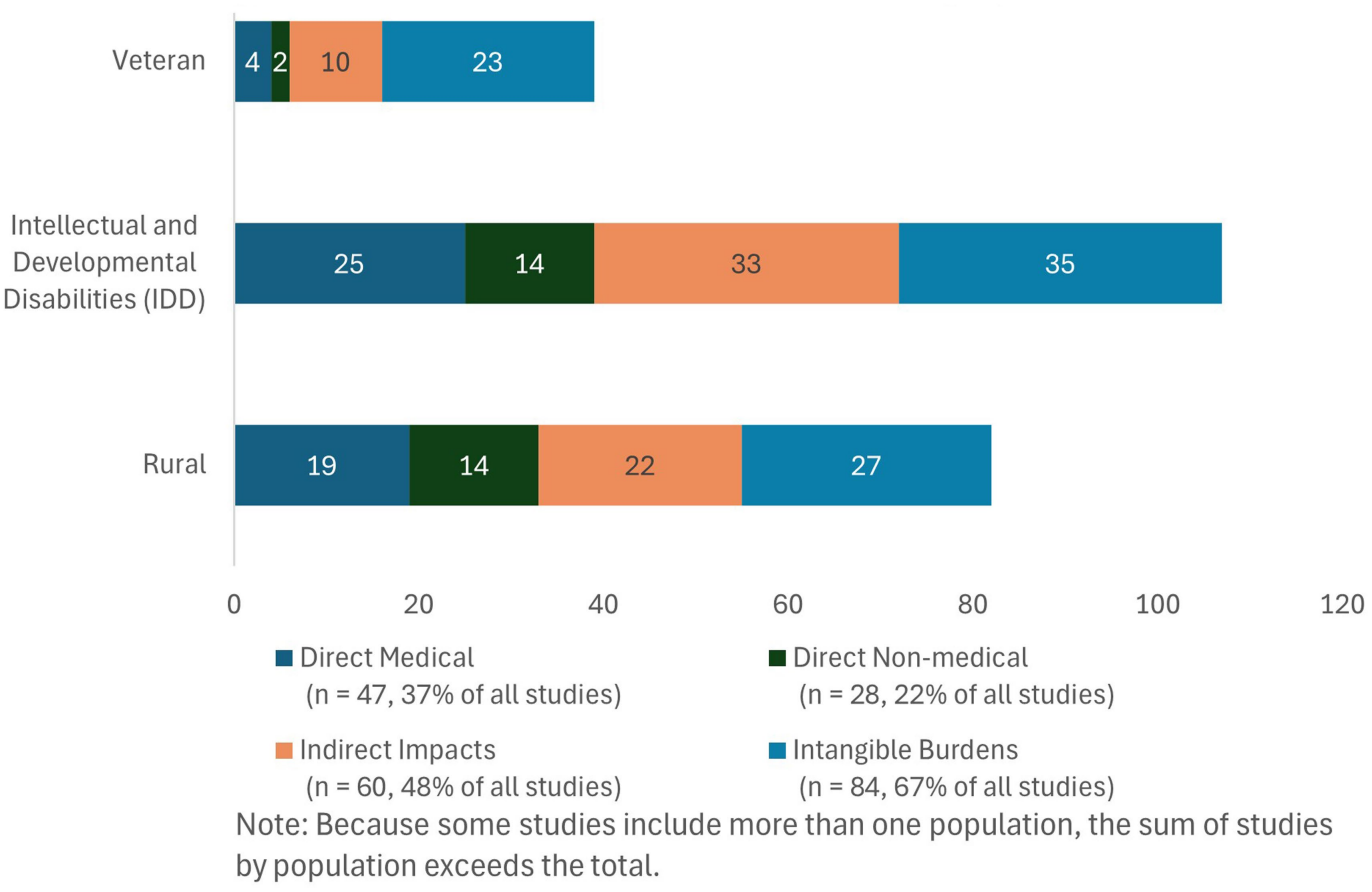
Distribution of PCBEOs across populations. Because some studies include more than one population, the sum of studies by population exceeds the total.

Our review highlights the distribution of types of PCBEOs researched across the three populations of interest. We found that the distribution of types of PCBEOs researched was consistent across populations: Intangible burdens were the most frequently investigated (*n* = 84, 67% of all studies reported intangible costs), followed by indirect costs (*n* = 60, 48%) and direct medical costs (*n* = 47, 37%); direct non-medical costs were the least commonly reported PCBEOs (*n* = 30, 24%) (see Figure 3).

Table 1 outlines the different types of direct medical costs investigated across veterans, people with IDD, and people living in rural areas. Studies on people with IDD reported direct medical costs most frequently (*n* = 25, 44% of all IDD studies), followed by studies on people living in rural areas (*n* = 19, 40% of all rural studies), whereas studies on veterans reported direct medical costs less often (*n* = 4, 15% of all veteran studies). Across all populations, copayments, coinsurance, deductibles, and premiums were the most common type of direct medical cost reported (*n* = 25, 20%). Certain types of direct medical costs were notably absent from studies on veterans. No studies on veterans investigated costs of diagnosis/treatment/management (beyond cost sharing), medical transportation, medication, or personal medical equipment or consumables. Studies on people with IDD similarly did not discuss costs of personal medical equipment or consumables but also did not investigate medical financial debt. Meanwhile, studies on people living in rural areas lacked investigations into medical transportation costs.

**Table 1:** Count of studies reporting on direct medical costs by population (*n* = 47, 37% of all studies).

Direct out-of-pocket (OOP) medical costs (*n*, % of all studies)	Veteran		Intellectual and developmental disabilities (IDD)		Rural	
	(*n* = 4, 15% of veteran studies)	Relevant citations	(*n* = 25, 44% of IDD studies)	Relevant citations	(*n* = 19, 40% of rural studies)	Relevant citations
Copayments, coinsurance, deductibles, premiums (*n* = 25, 20%)	3 (11%)	(13, 70, 71)	14 (25%)	(15, 49, 72–83)	9 (19%)	(53–55, 59, 77, 84–87)
Diagnosis/treatment/management costs (beyond cost sharing) (*n* = 11, 9%)	0 (0%)		4 (7%)	(15, 49, 66, 74)	7 (15%)	(53–56, 59, 88, 89)
Medical transportation costs (*n* = 1, 1%)	0 (0%)		1 (2%)	(80)	0 (0%)	
Medication costs (*n* = 12, 10%)	0 (0%)		5 (9%)	(49, 79–81, 90)	7 (15%)	(53–55, 84, 85, 91, 92)
Personal medical equipment or consumables costs (*n* = 4, 3%)	0 (0%)		0 (0%)		4 (9%)	(53, 56, 59, 92)
Medical financial debt (*n* = 2, 2%)	1 (4%)	(71)	0 (0%)		1 (2%)	(93)
Other/general direct medical costs (*n* = 12, 10%)	1 (4%)	(34)	9 (16%)	(47, 51, 94–100)	2 (4%)	(10, 84, 101)

Cost of using an ambulance is an example of medical transportation cost. Examples of costs of personal medical equipment or consumables include costs of continuous positive airway pressure (CPAP) machines, syringes, gauze and bandages, glucometers, blood glucose test strips, and insulin pumps. Other/general direct medical costs included general financial burden, composite OOP costs, spending per year on healthcare, and spending on medical care for children with ASD. Because some studies include more than one population, the sum of studies by population exceeds the total number of studies reporting direct medical costs. Within-population percentages may not sum to 100% because not all studies included a direct medical cost and 16 studies examined multiple direct medical costs. PCBEOs are not differentiated by whether they were experienced by patients or caregivers in this table and studies presented here may reflect outcomes reported for either group.

Table 2 details the types of direct non-medical costs that each of the three populations reported. Direct non-medical costs were discussed in 14 (30%) studies on people living in rural areas and 14 (25%) studies on people with IDD. Relatively few studies on veterans reported on direct non-medical costs (*n* = 2, 7%). The most frequently reported direct non-medical costs were for transportation, meals, and hoteling (*n* = 20, 16%). Although only one study on veterans reported on this cost, studies on people with IDD (*n* = 9, 16%) and people living in rural areas (*n* = 12, 26%) more commonly assessed travel costs in the direct non-medical cost category. All other direct non-medical costs were less frequently reported, including child/dependent care costs (*n* = 6, 5%) and other direct non-medical costs (*n* = 1, 1%). No studies examined costs of car or home modifications.

**Table 2:** Count of studies reporting on direct non-medical costs by population (n = 28, 22% of all studies).

Direct non-medical costs (*n*, % of all studies)	Veteran		Intellectual and developmental disabilities (IDD)		Rural	
	(*n* = 2, 7% of veteran studies)	Relevant citations	(*n* = 14, 25% of IDD studies)	Relevant citations	(*n* = 14, 30% of rural studies)	Relevant citations
Car or home modification costs (*n* = 0, 0%)	0 (0%)		0 (0%)		0 (0%)	
Child/dependent care costs (*n* = 6, 5%)	1 (4%)	(41)	4 (7%)	(65, 77, 102, 103)	2 (4%)	(77, 104)
Transportation, meals, hoteling costs (*n* = 20, 16%)	1 (4%)	(70)	9 (16%)	(72, 73, 75, 77–79, 102, 105, 106)	12 (26%)	(9, 53, 58, 59, 62, 77, 86, 105, 107–110)
Other/general direct non-medical costs (*n* = 1, 1%)	0 (0%)		1 (2%)	(94)	0 (0%)	

Other/general direct non-medical costs include moving costs to be closer to medical care. Because some studies include more than one population, the sum of studies by population exceeds the total number of studies. Within-population percentages may not sum to 100% because not all studies included a direct non-medical cost and two studies examined multiple types of direct non-medical costs. PCBEOs are not differentiated by whether they were experienced by patients or caregivers in this table and studies presented here may reflect outcomes reported for either group.

As demonstrated in Table 3, studies commonly reported indirect costs for each of the three populations (*n* = 60, 48%). Studies of people with IDD most frequently reported these costs (*n* = 33, 58%), followed by studies of people living in rural areas (*n* = 22, 47%) and veterans (*n* = 10, 37%). Most frequently reported types of indirect costs were unpaid caregiving time (*n* = 28, 22%), unpaid time off work and early retirement (*n* = 25, 20%), and travel time to or between medical appointments (*n* = 18, 14%). Two types of indirect impacts—increased time required for daily tasks and time spent navigating insurance or billing—were examined exclusively in studies of people with IDD, each investigated in only one study.

**Table 3:** Count of studies reporting on indirect impacts by population (*n* = 60, 48% of all studies).

Indirect impacts (*n*, % of all studies)	Veteran		Intellectual and developmental disabilities (IDD)		Rural	
	(*n* = 10, 37% of veteran studies)	Relevant citations	(*n* = 33, 58% of IDD studies)	Relevant citations	(*n* = 22, 47% of rural studies)	Relevant citations
Increased time required for daily tasks (*n* = 1, 1%)	0 (0%)		1 (2%)	(111)	0 (0%)	
Time to attend medical appointments (*n* = 7, 6%)	1 (4%)	(70)	3 (5%)	(75, 78, 106)	3 (6%)	(8, 9, 86)
Time spent navigating insurance or billing (*n* = 1, 1%)	0 (0%)		1 (2%)	(111)	0 (0%)	
Time spent providing unpaid caregiving (*n* = 28, 22%)	6 (22%)	(13, 34, 37, 112–114)	18 (32%)	(43, 44, 46–48, 50, 51, 65, 66, 79, 80, 96, 98, 100, 103, 115–117)	4 (9%)	(57, 60, 63, 118)
Time spent traveling to or between medical appointments (*n* = 18, 14%)	4 (15%)	(13, 70, 119, 120)	4 (7%)	(77, 105, 106, 111)	14 (30%)	(8, 62, 77, 85, 91, 104, 105, 107, 108, 110, 119–122)
Unpaid time off work, early retirement (*n* = 25, 20%)	2 (7%)	(13, 123)	18 (32%)	(48, 50, 51, 65, 66, 77, 79, 80, 90, 94, 98, 100, 102, 106, 117, 124–126)	6 (13%)	(8, 9, 62, 77, 86, 104)
Other/general indirect impacts (*n* = 6, 5%)	0 (0%)		5 (9%)	(97, 106, 124, 127, 128)	2 (4%)	(127, 129)

Other/general indirect impacts include disruptions to daily life/schedule, absenteeism and presentism, time spent preparing for appointments, school absenteeism, and composite indirect costs. Because some studies include more than one population, the sum of studies by population exceeds the total number of studies. Within-population percentages may not sum to 100% because not all studies included an indirect impact and 21 studies examined multiple types of indirect impacts. PCBEOs are not differentiated by whether they were experienced by patients or caregivers in this table and studies presented here may reflect outcomes reported for either group.

Table 4 details the distribution of intangible burdens within and across populations of interest, which were the most frequently investigated type of PCBEO overall (*n* = 84, 67%) and within each population—appearing in 85% of studies on veterans (*n* = 23), 61% of studies on people with IDD (*n* = 35), and 57% of studies on people living in rural areas (*n* = 27). Across the 12 types of intangible burdens, composite measures of caregiving (which summarize non-monetary caregiving burden into an overall score) were the most common, appearing in 37% (*n* = 46) of all studies and examined more often among studies on veterans (*n* = 17, 63%) and people with IDD (*n* = 22, 39%), compared with studies on people living in rural areas (*n* = 8, 17%). In contrast, studies on people living in rural areas most frequently examined delayed or forgone medical treatment (*n* = 13, 28%). Across all populations, the second most common intangible burden was financial toxicity (*n* = 21, 17%) and the third most common was delaying or forgoing medical treatment (*n* = 19, 15%). Variation in scope of included burdens was also evident, with studies on veterans investigating the narrowest set of burdens (omitting 7 of the 12 types), studies on people living in rural areas omitting 4, and studies on people with IDD covering the broadest range (lacking only burden of food and housing insecurity).

**Table 4:** Count of studies reporting on intangible burdens by population (n = 84, 67% of all studies).

Intangible burdens (*n*, % of all studies)	Veteran		Intellectual and developmental disabilities (IDD)		Rural	
	(*n* = 23, 85% of veteran studies)	Relevant citations	(*n* = 35, 61% of IDD studies)	Relevant citations	(*n* = 27, 57% of rural studies)	Relevant citations
Delaying or forgoing medical treatments due to costs (*n* = 19, 15%)	2 (7%)	(40, 71)	5 (9%)	(7, 78, 127, 130, 131)	13 (28%)	(8, 55, 56, 59, 61, 84, 108, 110, 127, 132–135)
Diminished household wealth (*n* = 4, 3%)	0 (0%)		2 (4%)	(50, 65)	2 (4%)	(89, 108)
Family/social life impacts (*n* = 3, 2%)	1 (4%)	(112)	2 (4%)	(50, 94)	0 (0%)	
Fear and anxiety about future ability to afford medical treatments (*n* = 1, 1%)	0 (0%)		1 (2%)	(65)	0 (0%)	
Changes in employment status and/or insurance coverage (*n* = 6, 5%)	0 (0%)		3 (5%)	(96, 103, 127)	4 (9%)	(8, 63, 108, 127)
Food insecurity, housing insecurity (*n* = 1, 1%)	0 (0%)		0 (0%)		1 (2%)	(108)
Hindered career or education advancement (*n* = 7, 6%)	3 (11%)	(34, 113, 123)	4 (7%)	(65, 94, 117, 126)	0 (0%)	
Quality-of-life impacts (not QALYs or DALYs) due to medical costs (*n* = 1, 1%)	0 (0%)		1 (2%)	(65)	0 (0%)	
Reduced spending on basic goods, groceries, and leisure activities (*n* = 3, 2%)	0 (0%)		1 (2%)	(136)	2 (4%)	(104, 108)
Distress, stress, and anxiety directly caused by out-of-pocket costs, financial debt, or lost income (i.e., financial toxicity) (*n* = 21, 17%)	5 (19%)	(37, 112, 113, 137, 138)	6 (11%)	(48, 50, 65, 103, 115, 125)	10 (21%)	(8, 9, 57, 60, 89, 101, 108, 110, 118, 139)
General or composite measure of caregiver burden (*n* = 46, 37%)	17 (63%)	(13, 35–39, 112–114, 140–147)	22 (39%)	(42–48, 50, 66, 111, 116, 117, 125–127, 148–154)	8 (17%)	(52, 57, 104, 122, 127, 129, 155, 156)
Other/general intangible burdens (*n* = 9, 7%)	0 (0%)		8 (14%)	(42–44, 51, 102, 117, 126, 127)	2 (4%)	(8, 127)

DALYs, disability-adjusted life-years; QALYs, quality-adjusted life-years. Other/general intangible burdens include general financial burden/problems, emotional burden, reduced self-care, and avoiding changing jobs due to concerns about health insurance. Because some studies include more than one population, the sum of studies by population exceeds the total number of studies. Within-population percentages may not sum to 100% because not all studies included an intangible burden and 23 studies contained multiple types of intangible burdens. PCBEOs are not differentiated by whether they were experienced by patients or caregivers in this table and studies presented here may reflect outcomes reported for either group.

##### 3.2.1.1 Patterns in PCBEOs among populations of interest

Patterns in the four broad categories of PCBEOs varied across populations. Studies identified in our review about veterans tended to concentrate on a single type of outcome, with 18 of 27 (67%) reporting only one PCBEO category and 9 (33%) reporting multiple types. In contrast, studies on people living in rural areas and people with IDD were more likely to examine multiple outcomes, with 20 of 47 (57%) studies on rural populations and 24 of 57 (58%) of studies on people with IDD reporting more than one of the four broad PCBEO categories.

Although most studies on veterans reported intangible burdens (*n* = 23, 85%), more than half focused exclusively on this category (*n* = 15, 56%). Across all burden categories, more than half of studies on veterans (*n* = 17, 63%) assessed the intangible burdens for caregivers (using general or composite caregiver burden measures). Indirect impacts were less frequently investigated than intangible burdens but still common (*n* = 10, 37%), whereas direct medical costs (*n* = 4, 15%) and direct non-medical costs (*n* = 2, 7%) were reported less frequently in studies on veterans.

Among studies focusing on people with IDD (*n* = 57), intangible burdens were also the most frequently reported (*n* = 35, 61%), followed closely by indirect costs (*n* = 33, 58%), direct medical costs (*n* = 25, 44%) and direct non-medical costs (*n* = 14, 25%). Similar to studies on veterans, general or composite measures of caregiver burden were the most frequently examined type of PCBEO in studies on people with IDD (*n* = 22, 39%), though they were less prevalent among IDD studies than veteran studies (*n* = 17, 63%). Other types of costs were reported at similar frequencies, including unpaid caregiving time (*n* = 18, 32%) and unpaid time off work or early retirement (*n* = 18, 32%).

For studies of people living in rural areas (*n* = 47), within each PCBEO category, the distribution of types of reported costs was more evenly spread than in studies of veterans or people with IDD, though still heavily focused on intangible burdens. Most studies on people living in rural areas reported intangible burdens (*n* = 27, 57%), followed by indirect costs (*n* = 22, 47%), direct medical costs (*n* = 19, 40%), and direct non-medical costs (*n* = 14, 30%). Unlike studies on veterans and people with IDD, the intangible burden of delaying or foregoing medical treatment due to cost (*n* = 13, 28%) in studies of people living in rural areas was investigated more frequently than composite measures of caregiver burden (*n* = 8, 17%).

#### 3.2.2 RQ2: among the PCBEO studies identified in RQ1, what are the population-specific justifications or reasons that the studies reported for the need to research PCBEOs for people living in rural areas, veterans, and people with IDD?

**Figure 4:**
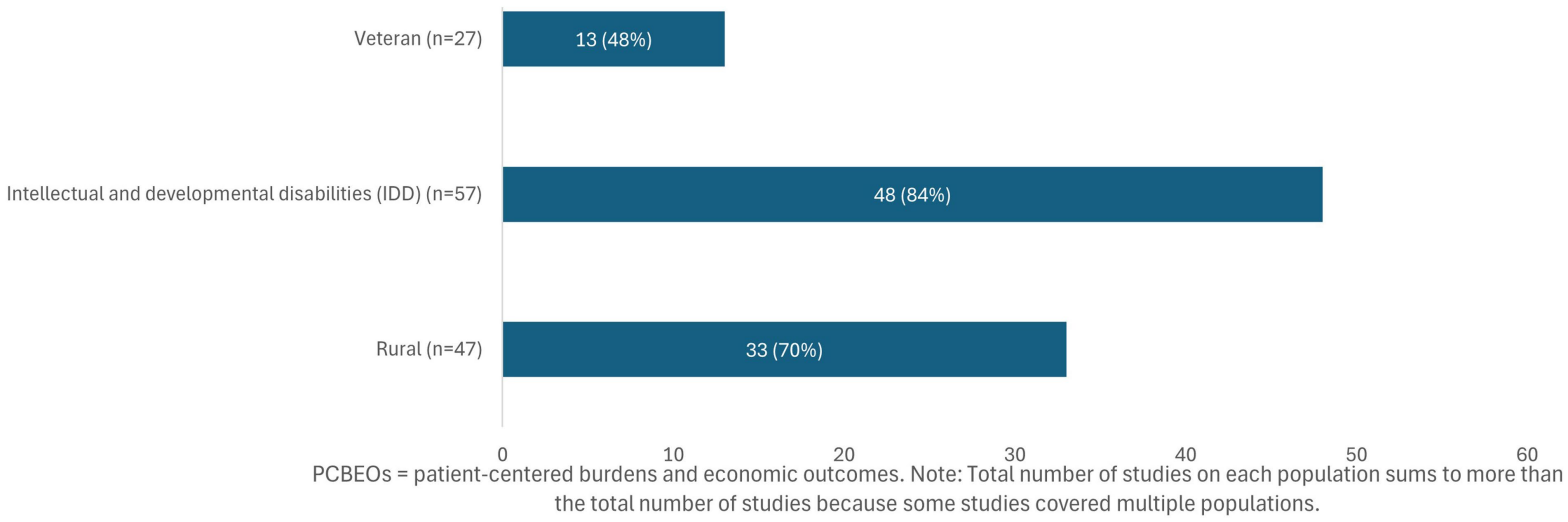
Frequency of studies with population-specific justifications for researching PCBEOs (*N* = 96). PCBEOs, patient-centered burdens and economic outcomes. Total number of studies on each population sums to more than the total number of studies because some studies covered multiple populations.

Figure 4 summarizes how often studies provided population-specific justifications for researching PCBEOs. Out of the 126 studies identified in RQ1, 96 (76%) included at least one justification tailored to the study population. The frequency of including justifications varied across the three populations of interest: Fewer than half of the studies on veterans (*n* = 13, 48%) described population-specific reasons, compared with 84% (*n* = 48) of studies on people with IDD and 70% (*n* = 33) of studies on people living in rural areas.

Table 5 summarizes types of justifications for conducting PCBEO research among the 96 studies that provided a population-specific rationale. Overall, the most common type of justifications across all groups cited research or data gaps (*n* = 58, 60% of all justifications), followed by need factors (*n* = 51, 53%) and enabling factors (*n* = 43, 45%). Additional information on the types of PCBEOs researched in the 96 studies that provided population-specific justifications is presented in Supplementary material F.

**Table 5:** Types of justifications by population (*n* = 96 articles justified).

Justification (*n*, % of justifications)	Veteran		Intellectual and developmental disabilities (IDD)		Rural	
	(*n* justified = 13, % of veteran-specific justifications)	Relevant citations	(*n* justified = 50, % of IDD-specific justifications)	Relevant citations	(*n* justified = 35, % of rural-specific justifications)	Relevant citations
Predisposing factors (*n* = 10, 10%)	2 (15%)	(38, 40)	3 (6%)	(94, 131, 136)	5 (15%)	(8, 52, 53, 89, 104)
Enabling factors (*n* = 43, 45%)	3 (23%)	(13, 143, 144)	15 (31%)	(7, 15, 42, 45, 47, 50, 75, 78, 80, 90, 94, 96, 106, 136, 149)	25 (76%)	(8–10, 52–63, 85, 89, 91, 93, 101, 107, 108, 118, 135, 155)
Need factors (*n* = 51, 53%)	10 (77%)	(34–41, 112, 144)	33 (69%)	(7, 15, 42–51, 65, 66, 73, 74, 80, 82, 96, 100, 102, 103, 105, 106, 111, 115, 117, 126, 128, 131, 149, 152, 153)	9 (27%)	(8–10, 54, 56, 59, 91, 105, 121)
Research/data gaps (*n* = 58, 60%)	8 (62%)	(13, 34–37, 112, 141, 143)	30 (63%)	(42–46, 48, 51, 65, 72–75, 78, 79, 81, 83, 90, 95, 100, 102, 115, 117, 124, 127, 148–151, 153, 154)	21 (64%)	(9, 52, 53, 56, 58, 60, 63, 86, 89, 91–93, 104, 107, 109, 121, 127, 129, 133, 135, 139)
Policy (*n* = 29, 30%)	5 (38%)	(13, 34, 41, 112, 141)	16 (33%)	(7, 15, 49, 66, 75, 76, 78, 83, 95, 100, 105, 111, 117, 127, 149, 154)	10 (30%)	(9, 10, 56, 57, 85, 105, 118, 127, 129, 133)

Total number of studies on each population sums to more than the total number of studies because some studies covered multiple populations. Within-population percentages may not sum to 100% because some studies contained multiple justifications.

Patterns of justifications varied notably across populations. Among veterans, the strongest emphasis was on need factors (*n* = 10, 77% of veteran-specific justifications), reflecting higher rates of chronic illnesses, disability status, and general health status related to serving in the military, such as traumatic brain injury (34–37), serious injury (38), mental health (39), and substance use disorders (39, 40). These conditions create substantial ongoing care needs and costs, often leading to a heavy reliance on unpaid caregivers, most frequently family members (38, 39). Veteran women in particular were noted as a population with distinct needs, especially regarding access to childcare and gender-specific services (40, 41).

For people with IDD, justifications were more frequently provided overall. Need factors (*n* = 33, 69% of IDD-specific justifications) and research/data gaps (*n* = 30, 63%) were most prominent. Articles emphasized the lifelong and intensive nature of caregiving because of patient need factors, particularly for people with ASD, which creates substantial physical, emotional, and financial strain for families (42–49). Many caregivers are “compound caregivers,” simultaneously providing care for adult children with disabilities and aging parents or other relatives, which intensifies demands (43–46). Families also face significant financial challenges from health needs specific to people with IDD, including poorer health outcomes and higher comorbidity prevalence among people with IDD (7), as well as the need for IDD-related therapies (49–51), specialized education (51), and residential care (50).

For people living in rural areas, the most common justification involved enabling factors (*n* = 25, 76% of rural-specific justifications), reflecting persistent barriers to healthcare access. These include geographic distance or isolation from providers (8–10, 52–57), transportation barriers (8, 54, 55, 58–61), and a limited or inadequate healthcare workforce in rural areas (8, 10, 53, 57, 62). Limited broadband infrastructure may also restrict access to virtual care (54, 57, 63). Although need factors (*n* = 9, 27%) were cited less often than enabling factors as population-specific justifications, people living in rural areas were reported to experience heightened health risks from environmental exposures, such as hearing loss from exposures to farm, factory, and construction equipment (56, 59).

### 3.3 Assessment of risk of Bias

We did not conduct a formal quality assessment of the included studies, as this was beyond the scope of the current review. As a result, we cannot make conclusions about the overall strength of the evidence. However, future systematic reviews or meta-analyses focused on more specific topics—such as the direct medical costs that veterans incur—may help address questions related to the consistency and robustness of the evidence.

## 4 Discussion

### 4.1 Summary of main findings

Research on PCBEOs that veterans, people with IDD, and people living in rural areas experienced has addressed a range of factors, including direct medical costs, direct non-medical costs, indirect impacts, and intangible burdens. A strength of this review is that it identifies gaps in the literature on PCBEOs for each of these populations, including the lack of focus on direct medical and non-medical costs. The literature on under-represented populations is too extensive for a single review, so we intentionally selected these groups to reflect a range of barriers, spanning clinical complexity and service needs to challenges associated with geographic isolation. Examining this spectrum of heterogenous groups enables us to compare PCBEOs across distinct structural and clinical contexts and identify shared drivers of burden that may not be apparent when each group is analyzed independently. This approach makes it possible to identify shared patterns that may inform understanding of similar burden types in other populations, while also recognizing context-specific differences that limit direct generalization.

From the included studies, intangible burdens were the most common PCBEOs investigated across the three groups, followed by indirect impacts, direct medical costs, and direct non-medical costs. For people living in rural areas, the distribution of studies focusing on each of the PCBEO categories was more evenly dispersed than for the other two populations examined, but with over half of the studies investigating intangible burdens. For veterans, the review revealed that most studies examining PCBEOs focused on intangible burdens and indirect impacts, whereas only five unique studies examined direct medical costs and direct non-medical costs for this population. Less than 40% of all veterans have VA coverage, and among all veterans, less than 30% used VA care for a healthcare visit in the past 12 months (64), highlighting two research needs for this population: (1) comprehensive accounting of out-of-pocket costs for veterans, and (2) a need for additional approaches for recruiting veterans to participate in research when investigating PCBEOs. For people with IDD, most of the research examined intangible burdens and indirect impacts with less literature focusing on the monetary costs (direct medical and direct non-medical) incurred by people with IDD. These findings identify a need for more research on direct medical costs and direct non-medical costs for each of these populations.

We also observed that studies on veterans were more likely to focus on a single type of PCBEO (namely, intangible burdens), in contrast with studies about other groups that more frequently reported on multiple categories of PCBEOs. This focus may reflect the distinct nature of caregiving within veteran populations, where care likely involves managing consequences of service-related injuries and psychological trauma. Thus, the literature may tend to emphasize the emotional and mental impacts of caregiving rather than a full accounting of economic costs. In contrast, studies on people with IDD and people living in rural areas appear to be more likely to apply multidimensional frameworks that assess a broader range of costs.

Furthermore, across all burden categories, over half of the studies that focused on veterans examined general or composite measures of caregiver burden. Although studies on people with IDD, and people living in rural areas also commonly explored composite caregiver burden measures, they did so at lower rates compared with studies on veterans. This pattern underscores a more frequent research emphasis on composite caregiver burden among veterans.

We found that most studies included at least one justification for researching PCBEOs tailored to the study population. However, the frequency of including a justification varied across the populations of interest, ranging from 48% of the studies on veterans to 84% of the studies on people with IDD. This suggests that IDD-related research more consistently framed PCBEOs as a distinct and important area of inquiry, whereas research on veterans and people living in rural areas provided less frequent justifications. Of the types of justifications provided, research or data gaps was the most common across all groups followed by need factors and enabling factors. Specifically, for veterans and people with IDD, justifications for reporting PCBEOs centered on need factors, such as high prevalence of chronic conditions, injuries, and mental health issues for veterans and high prevalence of comorbidities for people with IDD. High need factors for these populations often require significant caregiving demands, leading to additional burdens and impacts on caregivers. For example, caregivers of veterans reported experiencing higher depression, financial strain, and burden compared with their civilian counterparts (39). For caregivers of people with IDD, intense caregiving demands were shown to limit their employment opportunities, earnings, and time for self-care, with many caregivers reporting missed work, stalled promotions, or leaving the workforce altogether (50, 51, 65, 66). For people living in rural areas, enabling factors dominated the justifications, reflecting barriers such as geographic isolation, transportation challenges, provider shortages, and limited broadband access. Rural residents face disproportionately high transportation costs to access healthcare due to long travel distances, limited availability of specialists, and shortages in local healthcare infrastructure (53, 58, 59). Although less frequently cited, health needs specific to people living in rural areas may intersect with enabling factors and compound the challenges faced by this population.

### 4.2 Limitations

The current scoping review has some limitations. Because of the large number of PCBEOs, we did not include specific search terms for each PCBEO subcategory in our search strategy. For example, we did not use specific terms for costs of car or home modifications, which may have led to the omission of some relevant studies addressing these types of PCBEOs. However, the inclusion of broader search terms for general out-of-pocket costs should have captured studies addressing these specific categories, thereby reducing the likelihood of significant gaps. Additionally, to enhance the relevance of the findings, we restricted the review to U.S.-based studies published in English, but this may have excluded insights from international research or non-English publications. Future reviews could expand inclusion criteria to encompass studies conducted in other languages and countries.

Additionally, although this review identified substantial evidence on PCBEOs among people with IDD, it did not differentiate between pediatric and adult populations. This lack of distinction limits the ability to understand how caregiver and patient burdens vary across the lifespan of people with IDD. Children and adults with IDD often face different medical, educational, and social service needs, which can result in distinct economic and caregiving challenges. Without age-specific analysis, important nuances in PCBEOs may be overlooked. However, an aim of this review was to identify existing evidence on PCBEOs for all people with IDD from which we have now uncovered an additional area of future research.

Last, although we documented whether each study included patients or caregivers, we did not distinguish whether PCBEOs were experienced by patients versus caregivers. As a result, we cannot fully disentangle role-specific effects, which may affect how the breadth of evidence appears across populations. This limitation is particularly relevant in the IDD literature, where caregivers are often used as proxy respondents to provide information on behalf of people with IDD (67, 68). Conversely, some studies may report caregiver burden based on patient perceptions rather than direct caregiver reports, which may also influence the interpretation of these experiences. The implications for burdens in these three populations, and their generalizability to other groups, should be interpreted with due consideration of the data limitations. Despite the limits on clarity and specificity, including both patients and caregivers in this analysis remains necessary because patients and caregivers are mutually impacted by one another’s burdens. We provide more detailed information on study populations and assessed PCBEOs for each study in Supplementary material E.

### 4.3 Implications for future research

The findings of this review highlight existing gaps in literature related to the two RQs and offer a foundation for future research and policy considerations. First, the review of the literature reveals a need for more systematic data collection on PCBEOs within specific populations. Gathering more comprehensive quantitative and qualitative data will improve understanding of how these burdens differ across groups. Additionally, expanding the limited research on direct medical and non-medical costs would strengthen the evidence base needed to inform and support targeted programs and policies aimed at assisting patients and caregivers facing PCBEOs. For example, the VA Program of Comprehensive Assistance for Family Caregivers provides financial support, training, and respite care to family members caring for seriously injured post-9/11 veterans. However, eligibility and support levels vary, and some populations (e.g., rural caregivers, those caring for veterans with non-visible injuries) may be underserved (69). Additional research on the direct costs, indirect impacts, and intangible burdens experienced by caregivers of veterans could justify broadening the criteria, support increased stipends/financial resources based on burden severity, or help targeted outreach to underserved groups.

This review focused on PCBEOs among three specific populations: veterans, people with IDD, and people living in rural areas. Although this review provides valuable insight into the unique burdens these groups face, PCBEOs are often specific to population characteristics and medical conditions. Therefore, future research should include reviews of additional populations who may experience distinct patient and caregiver burdens. One extension of this work could be to focus on female veterans or those without VA coverage. Continuing to delve into the existing research on specific populations would help ensure a more comprehensive understanding of how PCBEOs vary across different contexts.

Finally, future work should include policy-oriented evaluations to assess how programs and system-level supports influence PCBEOs and to guide the design of structures that better support patients and caregivers, particularly in contexts where eligibility criteria or resource distribution may leave certain groups underserved.

## 5 Conclusion

This scoping review sheds light on the complex landscape of PCBEOs that veterans, people with IDD, and people living in rural areas face. The findings suggest that although significant strides have been made in understanding the challenges these populations face, considerable gaps still exist, particularly regarding direct medical and non-medical costs.

The evidence reveals that intangible burdens are the most frequently reported types of PCBEOs, emphasizing the emotional and psychological strains that caregivers and patients endure. Importantly, the variability in research focus among the different populations indicates a critical need for more targeted studies that capture the full range of PCBEOs that each group experienced. Understanding these differences will help develop effective strategies that address these populations’ unique needs. By enhancing our knowledge of PCBEOs, we can better inform interventions and policies aimed at improving health outcomes and overall well-being for these individuals and their caregivers.

## Data Availability

The original contributions presented in the study are included in the article/Supplementary material, further inquiries can be directed to the corresponding author.
