# Supplementary material for: Patient-centered burdens and economic outcomes among patients who are veterans, people with intellectual and developmental disabilities, and people living in rural areas and their caregivers: a scoping review
Source: Front Public Health. 2026 Apr 21;14:1749239. doi: 10.3389/fpubh.2026.1749239 (PMC13139145; doi:10.3389/fpubh.2026.1749239)
Supplement: Supplementary file 1 [file Data_Sheet_1.DOCX]

Supplementary Material

# Supplement A: Search Strategy

**Date Retrieved:** Access to Caregiving and Economic Impacts of Caregiving

**Timeframe:** January 1, 2015 – December 31, 2025

**Language:** English

**Geographic Area:** United States

**Publication Types:** Peer-reviewed (excluding literature reviews, book sections, comments, letters, and editorials)

**Databases:** PubMed, CINAHL, EconLit, Web of Science, APA PsycInfo

PubMed

#1 ("adult*"[ti] OR "caregiv*"[ti] OR "famil*"[ti] OR "children"[ti] OR "pediatric*"[ti] OR "patient*"[ti] OR "adult*"[ot] OR "caregiv*"[ot] OR "famil*"[ot] OR "children"[ot] OR "pediatric*"[ot] OR "patient*"[ot] OR "Caregivers"[Mesh] OR "Family"[Mesh:NoExp] OR "Patients"[Mesh:NoExp])

**AND**

("Rural Population"[Mesh] OR “rural*”[tiab] OR “remote area*”[tiab] OR "remote population*"[tiab] OR "remote communit*"[tiab] OR "hard-to-reach"[tiab] OR "hard to reach"[tiab] OR "frontier area"[tiab]

**OR**

"Veterans"[Mesh] OR “veteran*”[tiab] OR "military"[tiab] OR “soldier*”[tiab] OR "armed forces"[tiab] OR “troop*”[tiab] OR "defense"[tiab] OR “warfighter*”[tiab] OR “service member*”[tiab]

**OR**

"Developmental Disabilities"[Mesh] OR "Intellectual Disability"[Mesh] OR "developmental disorder*"[tiab] OR "Learning Disabilities"[Mesh] OR "Child Development Disorders, Pervasive"[Mesh] OR "mental retardation"[tiab] OR "mental subnormalit*"[tiab] OR "mental insufficienc*"[tiab] OR "intellectual impairment*"[tiab] OR "mental deficienc*"[tiab] OR "neurodevelopmental disorder*"[tiab] OR "neurological disorder*"[tiab] OR neurodiver*[tiab] OR ((developmental*[tiab] OR intellectual*[tiab]) AND (disab*[tiab] OR disorder*[tiab] OR delay*[tiab])) OR (learn*[tiab] AND (disab*[tiab] OR disorder*[tiab])) OR "Autistic Disorder"[Mesh] OR autis*[tiab] OR "Autism Spectrum Disorder"[Mesh] OR "Asperger Syndrome"[Mesh] OR asperger*[tiab] OR "Fetal Alcohol Spectrum Disorders"[Mesh] OR "multiple malformation syndrome"[tiab] OR "metabolic encephalopathy"[tiab] OR "congenital hypothyroidism"[tiab] OR "Down Syndrome"[Mesh] OR "Attention Deficit Disorder with Hyperactivity"[Mesh] OR "ADHD"[tiab] OR "Attention Deficit Hyperactivity Disorder"[Mesh] OR "Cerebral Palsy"[Mesh] OR "Hearing Loss"[Mesh] OR "Deafness"[Mesh] OR "Fragile X Syndrome"[Mesh] OR "Tourette Syndrome"[Mesh] OR "Kernicterus"[Mesh] OR "neonatal jaundice"[tiab]) AND ("2015/01/01"[Date - Publication] : "3000"[Date - Publication]) Filters: English 105,430

#2 ("copay*"[tiab] OR "co-pay*"[tiab] OR "deductible*"[tiab] OR "coinsurance"[tiab] OR "medical cost*"[tiab] OR "medical expend*"[tiab] OR "medication cost*"[tiab] OR "medication expend*"[tiab] OR "medications cost*"[tiab] OR "medications expend*"[tiab] OR "health cost*"[tiab] OR "health expend*"[tiab] OR "healthcare cost*"[tiab] OR "healthcare expend*"[tiab] OR "health care cost*"[tiab] OR "health care expend*"[tiab] OR "Drug Costs"[Mesh:NoExp] OR "Health Care Costs"[Mesh:NoExp] OR "Direct Service Costs"[Mesh:NoExp] OR "Health Expenditures"[Mesh:NoExp] OR "Economics, Medical"[Mesh:NoExp] OR (("transportation"[tiab] OR "parking"[tiab] OR "Food"[Mesh] OR "accommodation*"[tiab] OR "hotel*"[tiab] OR "Child Care"[Mesh] OR "Nonprescription Drugs" [Mesh]) AND (cost*[tiab] OR expens*[tiab] OR expend*[tiab] OR spend*[tiab])) OR "missed work"[tiab] OR "paid leave"[tiab] OR "paid time off"[tiab] OR "time cost*"[tiab] OR "unpaid leave"[tiab] OR "unpaid time off"[tiab] OR "work loss"[tiab] OR "missed work"[tiab] OR "out-of-pocket"[tiab] OR "Absenteeism"[Mesh] OR "Presenteeism"[Mesh] OR "lost wages"[tiab] OR "indirect cost*"[tiab] OR (("intangible*"[tiab] OR "spillover effect*"[tiab] OR "psychological impact*"[tiab] OR "emotional impact*"[tiab] OR "quality of life"[tiab] OR "leisure"[tiab] OR "distress*"[tiab] OR "anxiet*"[tiab] OR "mental health"[tiab] OR "wellbeing"[tiab] OR "well-being"[tiab] OR "productivity"[tiab] OR "stress"[tiab] OR "worry"[tiab] OR "coping"[tiab] OR "Quality of Life"[Mesh] OR "Psychological Distress"[Mesh:NoExp] OR "Anxiety"[Mesh:NoExp] OR "Mental Health"[Mesh] OR "Psychological Well-Being"[Mesh] OR "Stress, Psychological"[Mesh:NoExp] OR "Adaptation, Psychological"[Mesh:NoExp]) AND ("economic burden*"[ti] OR "financial toxicity"[ti] OR "financial hardship*"[ti] OR "financial burden*"[ti] OR "financial effect*"[ti] OR "financial stress*"[ti] OR "economic stress*"[ti] OR "financial constraint*"[ti] OR "financial distress*"[ti] OR "financial problem*"[ti] OR "financial pressure*"[ti] OR "financial crisis"[ti] OR "financial trouble*"[ti] OR "financial challenge*"[ti] OR "financial difficult*"[ti] OR "economic hardship*"[ti] OR "economic difficult*"[ti] OR "financial pressure*"[ti] OR "financial strain*"[ti] OR "financial repercussion*"[ti] OR "financial consequence*"[ti] OR "medical debt"[ti] OR "bankruptcy"[ti] OR "material hardship*"[ti] OR "Economics, Medical"[Mesh:NoExp] OR "Financial Stress"[Mesh])) OR "burden*"[ti] OR "caregiver burden*"[ti] OR "caregiving burden*"[ti] OR "economic impact*"[ti] OR "financial cost*"[ti] OR "out-of-pocket"[ti] OR "patient burden*"[ti] OR "burden*"[ot] OR "caregiver burden*"[ot] OR "caregiving burden*"[ot] OR "economic impact*"[ot] OR "financial cost*"[ot] OR "out-of-pocket"[ot] OR "patient burden*"[ot] OR "financial cost*"[ot] OR "Caregiver Burden"[Mesh]) AND ("2015/01/01"[Date - Publication] : "3000"[Date - Publication]) Filters: English 134,894

#3 ("United States"[Mesh] OR "United States"[tiab] OR "USA"[tiab] OR "U.S.A."[tiab] OR "U.S."[tiab] OR "United States"[Affiliation] OR "USA"[Affiliation] OR "U.S.A."[Affiliation] OR "U.S."[Affiliation] OR "US"[Affiliation]) AND ("2015/01/01"[Date - Publication] : "3000"[Date - Publication]) Filters: English 3,026,909

#4 (("Animals"[Mesh] NOT "Humans"[Mesh]) OR pmcbook OR pmcbooktitle OR pmcbookchapter OR "Comment"[Publication Type] OR "Letter"[Publication Type] OR "Editorial"[Publication Type] OR "adverse childhood experience*"[ti] OR "air pollution"[ti] OR "child abuse"[ti] OR "child neglect"[ti] OR "climate change"[ti] OR "elder abuse"[ti] OR "homicide*"[ti] OR "rat"[ti] OR "rats"[ti] OR "sexual abuse"[ti] OR "violence"[ti] OR "Australia*"[tiab] OR "Canada"[tiab] OR "Canadian*"[tiab] OR "China"[tiab] OR "German*"[tiab] OR global*[ti] OR "low- and middle-income countr*"[tiab] OR "Sub-Saharan Africa"[tiab] OR "United Kingdom"[tiab]) AND ("2015/01/01"[Date - Publication] : "3000"[Date - Publication]) Filters: English 2,767,767

#5 ((#1 AND #2 AND #3) NOT #4) AND ("2015/01/01"[Date - Publication] : "3000"[Date - Publication]) Filters: English

CINAHL

#1 (TI "adult*" OR TI "caregiv*" OR TI "famil*" OR TI "children" OR TI "pediatric*" OR TI "patient*" OR MH "Caregivers" OR MH "Family" OR MH "Patients")

**AND**

(MH "Rural Population" OR MH "Veterans+" OR MH "Developmental Disabilities" OR MH "Intellectual Disability+" OR MH "Learning Disorders+" OR MH "Child Development Disorders, Pervasive+" OR MH "Autism Spectrum Disorder" OR MH "Asperger Syndrome" OR MH "Fetal Alcohol Syndrome" OR MH "Down Syndrome" OR MH "Attention Deficit Hyperactivity Disorder" OR MH "Cerebral Palsy" OR MH "Hearing Disorders+" OR MH "Deafness+" OR MH "Fragile X Syndrome" OR MH "Tourette Syndrome" OR MH "Kernicterus"

**OR**

XB “rural*” OR XB “remote area*” OR XB "remote population*" OR XB "remote communit*" OR XB "hard-to-reach" OR XB "hard to reach" OR XB "frontier area"

**OR**

XB “veteran*” OR XB "military" OR XB “soldier*” OR XB "armed forces" OR XB “troop*” OR XB "defense" OR XB “warfighter*” OR XB “service member*”

**OR**

XB "developmental disorder*" OR XB "mental retardation" OR XB "mental subnormalit*" OR XB "mental insufficienc*" OR XB "intellectual impairment*" OR XB "mental deficienc*" OR XB "neurodevelopmental disorder*" OR XB "neurological disorder*" OR XB neurodiver* OR ((XB developmental* OR XB intellectual*) AND (XB disab* OR XB disorder* OR XB delay*)) OR (XB learn* AND (XB disab* OR XB disorder*)) OR XB autis* OR XB asperger* OR XB "multiple malformation syndrome" OR XB "metabolic encephalopathy" OR XB "congenital hypothyroidism" OR XB "ADHD" OR XB "neonatal jaundice") Limiters - Publication Date: 20150101-20251231; English Language; Peer Reviewed; Exclude MEDLINE records 33,655

#2 (XB "copay*" OR XB "co-pay*" OR XB "deductible*" OR XB "coinsurance" OR XB "medical cost*" OR XB "medical expend*" OR XB "medication cost*" OR XB "medication expend*" OR XB "medications cost*" OR XB "medications expend*" OR XB "health cost*" OR XB "health expend*" OR XB "healthcare cost*" OR XB "healthcare expend*" OR XB "health care cost*" OR XB "health care expend*" OR ((XB "transportation" OR XB "parking" OR XB "accommodation*" OR XB "hotel*" OR MH "Food+" OR MH "Child Care+" OR MH "Drugs, Non-Prescription") AND (XB cost* OR XB expens* OR XB expend* OR XB spend*)) OR XB "missed work" OR XB "paid leave" OR XB "paid time off" OR XB "time cost*" OR XB "unpaid leave" OR XB "unpaid time off" OR XB "work loss" OR XB "missed work" OR XB "out-of-pocket" OR XB "lost wages" OR XB "indirect cost*" OR ((XB "intangible*" OR XB "spillover effect*" OR XB "psychological impact*" OR XB "emotional impact*" OR XB "quality of life" OR XB "leisure" OR XB "distress*" OR XB "anxiet*" OR XB "mental health" OR XB "wellbeing" OR XB "well-being" OR XB "productivity" OR XB "stress" OR XB "worry" OR XB "coping" OR MH "Quality of Life+" OR MH "Psychological Distress" OR MH "Anxiety" OR MH "Mental Health" OR MH "Psychological Well-Being" OR MH "Stress, Psychological" OR MH "Adaptation, Psychological") AND (TI "economic burden*" OR TI "financial toxicity" OR TI "financial hardship*" OR TI "financial burden*" OR TI "financial effect*" OR TI "financial stress*" OR TI "economic stress*" OR TI "financial constraint*" OR TI "financial distress*" OR TI "financial problem*" OR TI "financial pressure*" OR TI "financial crisis" OR TI "financial trouble*" OR TI "financial challenge*" OR TI "financial difficult*" OR TI "economic hardship*" OR TI "economic difficult*" OR TI "financial pressure*" OR TI "financial strain*" OR TI "financial repercussion*" OR TI "financial consequence*" OR TI "medical debt" OR TI "bankruptcy" OR TI "material hardship*" OR MH "Financial Stress")) OR TI "burden*" OR TI "caregiver burden*" OR TI "caregiving burden*" OR TI "economic impact*" OR TI "financial cost*" OR TI "out-of-pocket" OR TI "patient burden*" OR MH "Health Care Costs" OR MH "Health Expenditures" OR MH "Absenteeism" OR MH "Presenteeism" OR MH "Caregiver Burden") Limiters - Publication Date: 20150101-20251231; English Language; Peer Reviewed; Exclude MEDLINE records 46,191

#3 ZZ "usa" OR MH "United States+" OR ZS "usa" OR AF "United States" OR AF "USA" OR AF "U.S.A." OR XB "United States" OR XB "USA" OR XB "U.S.A." Limiters - Publication Date: 20150101-20251231; English Language; Peer Reviewed; Exclude MEDLINE records 1,406,024

#4 ((MH "Animals+" NOT MH "Human") OR MH "Animal Studies" OR ZT "book" OR ZT "book chapter" OR ZT "commentary" OR ZT "editorial" OR ZT "letter" OR ZT "letter to the editor" OR TI "adverse childhood experience*" OR TI "air pollution" OR TI "child abuse" OR TI "child neglect" OR TI "climate change" OR TI "elder abuse" OR TI "homicide*" OR TI "rat" OR TI "rats" OR TI "sexual abuse" OR TI "violence" OR TI global* OR XB "Australia*" OR XB "Canada" OR XB "Canadian*" OR XB "China" OR XB "German*" OR XB "low- and middle-income countr*" OR XB "Sub-Saharan Africa" OR XB "United Kingdom") Limiters - Publication Date: 20150101-20251231; English Language; Peer Reviewed; Exclude MEDLINE records 266,509

#5 ((#1 AND #2 AND #3) NOT #4) Limiters - Publication Date: 20150101-20251231; English Language; Peer Reviewed; Exclude MEDLINE records

EconLit

#1 (TI "adult*" OR TI "caregiv*" OR TI "famil*" OR TI "children" OR TI "pediatric*" OR TI "patient*" OR SU "Caregivers" OR SU "Family" OR SU "Patients")

**AND**

(SU "Rural Population" OR “rural*” OR “remote area*” OR "remote population*" OR "remote communit*" OR "hard-to-reach" OR "hard to reach" OR "frontier area"

**OR**

SU "Veterans" OR “veteran*” OR "military" OR “soldier*” OR "armed forces" OR “troop*” OR "defense" OR “warfighter*” OR “service member*”

**OR**

SU "Developmental Disabilities" OR SU "Intellectual Disability" OR "developmental disorder*" OR SU "Learning Disabilities" OR SU "Child Development Disorders, Pervasive" OR "mental retardation" OR "mental subnormalit*" OR "mental insufficienc*" OR "intellectual impairment*" OR "mental deficienc*" OR "neurodevelopmental disorder*" OR "neurological disorder*" OR neurodiver* OR ((developmental* OR intellectual*) AND (disab* OR disorder* OR delay*)) OR (learn* AND (disab* OR disorder*)) OR SU "Autistic Disorder" OR autis* OR SU "Autism Spectrum Disorder" OR SU "Asperger Syndrome" OR asperger* OR SU "Fetal Alcohol Spectrum Disorders" OR "multiple malformation syndrome" OR "metabolic encephalopathy" OR "congenital hypothyroidism" OR SU "Down Syndrome" OR SU "Attention Deficit Disorder with Hyperactivity" OR "ADHD" OR SU "Attention Deficit Hyperactivity Disorder" OR SU "Cerebral Palsy" OR SU "Hearing Loss" OR SU "Deafness" OR SU "Fragile X Syndrome" OR SU "Tourette Syndrome" OR SU "Kernicterus" OR "neonatal jaundice") AND ZL "english" Limiters - Publication Date: 20150101-20251231 3,052

#2 ("copay*" OR "co-pay*" OR "deductible*" OR "coinsurance" OR "medical cost*" OR "medical expend*" OR "medication cost*" OR "medication expend*" OR "medications cost*" OR "medications expend*" OR "health cost*" OR "health expend*" OR "healthcare cost*" OR "healthcare expend*" OR "health care cost*" OR "health care expend*" OR SU "Drug Costs" OR SU "Health Care Costs" OR SU "Direct Service Costs" OR SU "Health Expenditures" OR SU "Economics, Medical" OR (("transportation" OR "parking" OR SU "Food" OR "accommodation*" OR "hotel*" OR SU "Child Care" OR SU "Nonprescription Drugs") AND (cost* OR expens* OR expend* OR spend*)) OR "missed work" OR "paid leave" OR "paid time off" OR "time cost*" OR "unpaid leave" OR "unpaid time off" OR "work loss" OR "missed work" OR "out-of-pocket" OR SU "Absenteeism" OR SU "Presenteeism" OR "lost wages" OR "indirect cost*" OR (("intangible*" OR "spillover effect*" OR "psychological impact*" OR "emotional impact*" OR "quality of life" OR "leisure" OR "distress*" OR "anxiet*" OR "mental health" OR "wellbeing" OR "well-being" OR "productivity" OR "stress" OR "worry" OR "coping" OR SU "Quality of Life" OR SU "Psychological Distress" OR SU "Anxiety" OR SU "Mental Health" OR SU "Psychological Well-Being" OR SU "Stress, Psychological" OR SU "Adaptation, Psychological") AND (TI "economic burden*" OR TI "financial toxicity" OR TI "financial hardship*" OR TI "financial burden*" OR TI "financial effect*" OR TI "financial stress*" OR TI "economic stress*" OR TI "financial constraint*" OR TI "financial distress*" OR TI "financial problem*" OR TI "financial pressure*" OR TI "financial crisis" OR TI "financial trouble*" OR TI "financial challenge*" OR TI "financial difficult*" OR TI "economic hardship*" OR TI "economic difficult*" OR TI "financial pressure*" OR TI "financial strain*" OR TI "financial repercussion*" OR TI "financial consequence*" OR TI "medical debt" OR TI "bankruptcy" OR TI "material hardship*" OR SU "Economics, Medical" OR SU "Financial Stress")) OR TI "burden*" OR TI "caregiver burden*" OR TI "caregiving burden*" OR TI "economic impact*" OR TI "financial cost*" OR TI "out-of-pocket" OR TI "patient burden*" OR SU "Caregiver Burden") AND ZL "english" Limiters - Publication Date: 20150101-20251231 28,639

#3 (SU "United States" OR TI "United States" OR TI "USA" OR TI "U.S.A." OR TI "U.S." OR AB "United States" OR AB "USA" OR AB "U.S.A." OR AB "U.S." OR ZG "u.s." OR ZW "united states") AND ZL "english" Limiters - Publication Date: 20150101-20251231 94,419

#4 ((SU "Animals" NOT SU "Humans") OR ZT "book" OR ZT "book review" OR ZT "dissertation" OR ZT "working paper" OR TI "adverse childhood experience*" OR TI "air pollution" OR TI "child abuse" OR TI "child neglect" OR TI "climate change" OR TI "elder abuse" OR TI "homicide*" OR TI "rat" OR TI "rats" OR TI "sexual abuse" OR TI "violence" OR TI global* OR TI "Australia*" OR TI "Canada" OR TI "Canadian*" OR TI "China" OR TI "German*" OR TI "low- and middle-income countr*" OR TI "Sub-Saharan Africa" OR TI "United Kingdom" OR AB "Australia*" OR AB "Canada" OR AB "Canadian*" OR AB "China" OR AB "German*" OR AB "low- and middle-income countr*" OR AB "Sub-Saharan Africa" OR AB "United Kingdom") AND ZL "english" Limiters - Publication Date: 20150101-20251231 150,947

#5 ((#1 AND #2 AND #3) NOT #4) AND ZL "english" Limiters - Publication Date: 20150101-20251231

Web of Science

#1 (TI=("adult*" OR "caregiv*" OR "famil*" OR "children" OR "pediatric*" OR "patient*") OR AK=("adult*" OR "caregiv*" OR "famil*" OR "children" OR "pediatric*" OR "patient*"))

**AND**

(AK=("Rural Population" OR "Veterans" OR "Developmental Disabilities" OR "Intellectual Disability" OR "Learning Disabilities" OR "Child Development Disorders, Pervasive" OR "Autistic Disorder" OR "Autism Spectrum Disorder" OR "Asperger Syndrome" OR "Fetal Alcohol Spectrum Disorders" OR "Down Syndrome" OR "Attention Deficit Disorder with Hyperactivity" OR "Attention Deficit Hyperactivity Disorder" OR "Cerebral Palsy" OR "Hearing Loss" OR "Deafness" OR "Fragile X Syndrome" OR "Tourette Syndrome" OR "Kernicterus")

**OR**

TI=(“rural*” OR “remote area*” OR "remote population*" OR "remote communit*" OR "hard-to-reach" OR "hard to reach" OR "frontier area" OR “veteran*” OR "military" OR “soldier*” OR "armed forces" OR “troop*” OR "defense" OR “warfighter*” OR “service member*” OR "developmental disorder*" OR "mental retardation" OR "mental subnormalit*" OR "mental insufficienc*" OR "intellectual impairment*" OR "mental deficienc*" OR "neurodevelopmental disorder*" OR "neurological disorder*" OR neurodiver* OR autis* OR asperger* OR "multiple malformation syndrome" OR "metabolic encephalopathy" OR "congenital hypothyroidism" OR "ADHD" OR "neonatal jaundice")

**OR**

AB=(“rural*” OR “remote area*” OR "remote population*" OR "remote communit*" OR "hard-to-reach" OR "hard to reach" OR "frontier area" OR “veteran*” OR "military" OR “soldier*” OR "armed forces" OR “troop*” OR "defense" OR “warfighter*” OR “service member*” OR "developmental disorder*" OR "mental retardation" OR "mental subnormalit*" OR "mental insufficienc*" OR "intellectual impairment*" OR "mental deficienc*" OR "neurodevelopmental disorder*" OR "neurological disorder*" OR neurodiver* OR autis* OR asperger* OR "multiple malformation syndrome" OR "metabolic encephalopathy" OR "congenital hypothyroidism" OR "ADHD" OR "neonatal jaundice")

**OR**

TS=((developmental* OR intellectual*) AND (disab* OR disorder* OR delay*)) OR TS=((learn* AND (disab* OR disorder*)))) Timespan: 2015-01-01 to 2025-12-31 (Publication Date) 130,187

#2 TI=("copay*" OR "co-pay*" OR "deductible*" OR "coinsurance" OR "medical cost*" OR "medical expend*" OR "medication cost*" OR "medication expend*" OR "medications cost*" OR "medications expend*" OR "health cost*" OR "health expend*" OR "healthcare cost*" OR "healthcare expend*" OR "health care cost*" OR "health care expend*" OR "missed work" OR "paid leave" OR "paid time off" OR "time cost*" OR "unpaid leave" OR "unpaid time off" OR "work loss" OR "missed work" OR "out-of-pocket" OR "lost wages" OR "indirect cost*" OR "burden*" OR "caregiver burden*" OR "caregiving burden*" OR "economic impact*" OR "financial cost*" OR "out-of-pocket" OR "patient burden*") OR AB=("copay*" OR "co-pay*" OR "deductible*" OR "coinsurance" OR "medical cost*" OR "medical expend*" OR "medication cost*" OR "medication expend*" OR "medications cost*" OR "medications expend*" OR "health cost*" OR "health expend*" OR "healthcare cost*" OR "healthcare expend*" OR "health care cost*" OR "health care expend*" OR "missed work" OR "paid leave" OR "paid time off" OR "time cost*" OR "unpaid leave" OR "unpaid time off" OR "work loss" OR "missed work" OR "out-of-pocket" OR "lost wages" OR "indirect cost*") OR AK=("burden*" OR "caregiver burden*" OR "caregiving burden*" OR "economic impact*" OR "financial cost*" OR "out-of-pocket" OR "patient burden*" OR "financial cost*" OR "Drug Costs" OR "Health Care Costs" OR "Direct Service Costs" OR "Health Expenditures" OR "Economics, Medical" OR "Absenteeism" OR "Presenteeism") OR TS=(("transportation" OR "parking" OR "Food" OR "accommodation*" OR "hotel*" OR "Child Care" OR "Nonprescription Drugs") AND (cost* OR expens* OR expend* OR spend*)) OR (TS=("intangible*" OR "spillover effect*" OR "psychological impact*" OR "emotional impact*" OR "quality of life" OR "leisure" OR "distress*" OR "anxiet*" OR "mental health" OR "wellbeing" OR "well-being" OR "productivity" OR "stress" OR "worry" OR "coping" OR "Quality of Life" OR "Psychological Distress" OR "Anxiety" OR "Mental Health" OR "Psychological Well-Being" OR "Stress, Psychological" OR "Adaptation, Psychological") AND (TI=("economic burden*" OR "financial toxicity" OR "financial hardship*" OR "financial burden*" OR "financial effect*" OR "financial stress*" OR "economic stress*" OR "financial constraint*" OR "financial distress*" OR "financial problem*" OR "financial pressure*" OR "financial crisis" OR "financial trouble*" OR "financial challenge*" OR "financial difficult*" OR "economic hardship*" OR "economic difficult*" OR "financial pressure*" OR "financial strain*" OR "financial repercussion*" OR "financial consequence*" OR "medical debt" OR "bankruptcy" OR "material hardship*") OR AK=("Economics, Medical" OR "Financial Stress"))) Timespan: 2015-01-01 to 2025-12-31 (Publication Date) 251,712

#3 AK=("Animals" NOT "Humans") OR TI=("adverse childhood experience*" OR "air pollution" OR "child abuse" OR "child neglect" OR "climate change" OR "elder abuse" OR "homicide*" OR "rat" OR "rats" OR "sexual abuse" OR "violence" OR global*) OR TS=("Australia*" OR "Canada" OR "Canadian*" OR "China" OR "German*" OR "low- and middle-income countr*" OR "Sub-Saharan Africa" OR "United Kingdom") Timespan: 2015-01-01 to 2025-12-31 (Publication Date) 2,005,906

#4 (#1 AND #2) NOT #3 and English (Languages) and USA (Countries/Regions) and non-USA (Exclude – Countries/Regions) and Letter or Editorial Material or Meeting Abstract or Proceeding Paper (Exclude – Document Types) Timespan: 2015-01-01 to 2025-12-31 (Publication Date)

APA PsycInfo

#1 (TI "adult*" OR TI "caregiv*" OR TI "famil*" OR TI "children" OR TI "pediatric*" OR TI "patient*" OR DE "Caregivers" OR DE "Family" OR DE "Patients")

**AND**

(DE "Rural Environments" OR DE "Military Veterans" OR DE "Developmental Disabilities" OR DE "Intellectual Development Disorder" OR DE "Learning Disorders" OR DE "Learning Disabilities" OR DE "Reading Disabilities" OR DE "Autism Spectrum Disorders" OR DE "Fetal Alcohol Syndrome" OR DE "Down's Syndrome" OR DE "Attention Deficit Disorder" OR DE "Attention Deficit Disorder with Hyperactivity" OR DE "Cerebral Palsy" OR DE "Hearing Disorders" OR DE "Hearing Loss" OR DE "Deafness" OR DE "Fragile X Syndrome" OR DE "Tourette Syndrome"

**OR**

XB “rural*” OR XB “remote area*” OR XB "remote population*" OR XB "remote communit*" OR XB "hard-to-reach" OR XB "hard to reach" OR XB "frontier area" OR XB “veteran*” OR XB "military" OR XB “soldier*” OR XB "armed forces" OR XB “troop*” OR XB "defense" OR XB “warfighter*” OR XB “service member*” OR XB "developmental disorder*" OR XB "mental retardation" OR XB "mental subnormalit*" OR XB "mental insufficienc*" OR XB "intellectual impairment*" OR XB "mental deficienc*" OR XB "neurodevelopmental disorder*" OR XB "neurological disorder*" OR XB neurodiver* OR ((XB developmental* OR XB intellectual*) AND (XB disab* OR XB disorder* OR XB delay*)) OR (XB learn* AND (XB disab* OR XB disorder*)) OR XB autis* OR XB asperger* OR XB "multiple malformation syndrome" OR XB "metabolic encephalopathy" OR XB "congenital hypothyroidism" OR XB "ADHD" OR XB "neonatal jaundice") Limiters - Publication Year: 2015-2025; Peer Reviewed; English language 37,799

#2 (XB "copay*" OR XB "co-pay*" OR XB "deductible*" OR XB "coinsurance" OR XB "medical cost*" OR XB "medical expend*" OR XB "medication cost*" OR XB "medication expend*" OR XB "medications cost*" OR XB "medications expend*" OR XB "health cost*" OR XB "health expend*" OR XB "healthcare cost*" OR XB "healthcare expend*" OR XB "health care cost*" OR XB "health care expend*" OR ((XB "transportation" OR XB "parking" OR XB "accommodation*" OR XB "hotel*" OR DE "Food" OR DE "Child Care" OR DE "Nonprescription Drugs") AND (XB cost* OR XB expens* OR XB expend* OR XB spend*)) OR XB "missed work" OR XB "paid leave" OR XB "paid time off" OR XB "time cost*" OR XB "unpaid leave" OR XB "unpaid time off" OR XB "work loss" OR XB "missed work" OR XB "out-of-pocket" OR XB "lost wages" OR XB "indirect cost*" OR ((XB "intangible*" OR XB "spillover effect*" OR XB "psychological impact*" OR XB "emotional impact*" OR XB "quality of life" OR XB "leisure" OR XB "distress*" OR XB "anxiet*" OR XB "mental health" OR XB "wellbeing" OR XB "well-being" OR XB "productivity" OR XB "stress" OR XB "worry" OR XB "coping" OR DE "Quality of Life" OR DE "Health Related Quality of Life" OR DE "Quality of Work Life" OR DE "Distress" OR DE "Anxiety" OR DE "Mental Health" OR DE "Emotional Well Being" OR DE "Psychological Stress" OR DE "Emotional Adjustment") AND (TI "economic burden*" OR TI "financial toxicity" OR TI "financial hardship*" OR TI "financial burden*" OR TI "financial effect*" OR TI "financial stress*" OR TI "economic stress*" OR TI "financial constraint*" OR TI "financial distress*" OR TI "financial problem*" OR TI "financial pressure*" OR TI "financial crisis" OR TI "financial trouble*" OR TI "financial challenge*" OR TI "financial difficult*" OR TI "economic hardship*" OR TI "economic difficult*" OR TI "financial pressure*" OR TI "financial strain*" OR TI "financial repercussion*" OR TI "financial consequence*" OR TI "medical debt" OR TI "bankruptcy" OR TI "material hardship*" OR DE "Financial Strain")) OR TI "burden*" OR TI "caregiver burden*" OR TI "caregiving burden*" OR TI "economic impact*" OR TI "financial cost*" OR TI "out-of-pocket" OR TI "patient burden*" OR DE "Health Care Costs" OR DE "Employee Absenteeism" OR DE "Caregiver Burden") Limiters - Publication Year: 2015-2025; Peer Reviewed; English language 16,843

#3 PL "us" OR ZY "us" OR ZU "united states" OR ZF "u.s." OR ZF "us" OR ZF "usa*" OR ZF "u.s.a.*" OR ZF "united states*" OR XB "United States" OR XB "USA" OR XB "U.S.A." OR XB "U.S." Limiters - Publication Year: 2015-2025; Peer Reviewed; English language 328,218

#4 ((ZP "animal" NOT ZP "human") OR ZZ "book" OR ZZ "chapter" OR ZZ "column/opinion" OR ZZ "comment/reply" OR ZZ "editorial" OR ZZ "letter" OR ZT "authored book" OR ZT "book" OR ZT "dissertation abstract" OR ZT "edited book" OR ZT "encyclopedia" OR TI "adverse childhood experience*" OR TI "air pollution" OR TI "child abuse" OR TI "child neglect" OR TI "climate change" OR TI "elder abuse" OR TI "homicide*" OR TI "rat" OR TI "rats" OR TI "sexual abuse" OR TI "violence" OR TI global* OR XB "Australia*" OR XB "Canada" OR XB "Canadian*" OR XB "China" OR XB "German*" OR XB "low- and middle-income countr*" OR XB "Sub-Saharan Africa" OR XB "United Kingdom") Limiters - Publication Year: 2015-2025; Peer Reviewed; English language 276,344

#5 ((#1 AND #2 AND #3) NOT #4) Limiters - Publication Year: 2015-2025; Peer Reviewed; English language

# Supplement B: Selection Criteria

|  | Inclusion | Exclusion |
| --- | --- | --- |
| Population | Patients and/or their families/caregivers experiencing physical, emotional, financial, or time-related burdens caused by a medical condition  Must include a population of interest (i.e., rural communities, veterans, people with IDD). | Studies focusing on populations other than patients and/or their families/caregivers (e.g., providers, payers, health systems)  Studies conducted outside the United States. |
| Intervention | Medical conditions causing physical, emotional, financial, or time-related burdens to patients and/or families/caregivers of patients who are in a population of interest | Burdens not caused by medical condition (e.g., economic disruptions from the COVID-19 pandemic, natural disasters, job loss unrelated to health conditions)  Burdens unrelated to patients or their families/caregivers (e.g., providers or health systems-level challenges) |
| Outcomes | RQ1  Research on types of PCBEOs that are particular to a group in one of the populations of interest  Patient and/or family/caregiver burdens (listed above) stratified by characteristics of the population of interest or focusing on a specific population of interest  RQ2  Impact of inclusion of the populations of interest in PCBEO research  Justifications or reasons discussed for measuring PCBEOs among the three populations of interest | Outcomes unrelated to physical, emotional, financial, or time-related burdens from medical conditions affecting patients or families/caregivers  Outcomes that do not focus on economic factors or PCBEOs |
| Setting/Publication Type | Studies conducted in the United States  Peer-reviewed articles published in 2015 or later  Publications available in English | Non-peer-reviewed publications such as book sections, letters, editorials, or commentaries  Dissertations, working papers, news articles, trade publications, or literature reviews  Study protocols without results or findings  Publications in languages other than English  Published before 2015 |

# Supplement C: Medical conditions by population (N=126)

| Medical conditions (n, % of all studies) | Veteran | | Intellectual and developmental disabilities (IDD) | | Rural | |
| --- | --- | --- | --- | --- | --- | --- |
|  | (n=27, % of veteran studies) | Relevant citations | (n=57, % of IDD studies) | Relevant citations | (n=47, % of rural studies) | Relevant citations |
| Alzheimer's disease (n=1, 1%) | 1 (4%) | (112) | 0 (0%) |  | 0 (0%) |  |
| Atrial fibrillation (n=1, 1%) | 0 (0%) |  | 0 (0%) |  | 1 (2%) | (89) |
| Cancer (n=14, 10%) | 1 (4%) | (135) | 0 (0%) |  | 13 (28%) | (53, 54, 62, 84, 90, 91, 99, 106, 107, 119, 127, 137, 154) |
| Bladder cancer (n=1, 1%) | 0 (0%) |  | 0 (0%) |  | 1 (2%) | (62) |
| Brain cancer (n=1, 1%) | 1 (4%) | (135) | 0 (0%) |  | 0 (0%) |  |
| Breast cancer (n=2, 2%) | 0 (0%) |  | 0 (0%) |  | 2 (4%) | (119, 137) |
| Colorectal cancer (n=1, 1%) | 0 (0%) |  | 0 (0%) |  | 1 (2%) | (119) |
| Lung cancer (n=1, 1%) | 0 (0%) |  | 0 (0%) |  | 1 (2%) | (119) |
| Spine metastases (n=0, 0%) | 1 (4%) | (135) | 0 (0%) |  | 0 (0%) |  |
| Cardiovascular disease (n=2, 2%) | 0 (0%) |  | 0 (0%) |  | 2 (4%) | (83, 131) |
| Heart failure (n=4, 3%) | 0 (0%) |  | 0 (0%) |  | 4 (9%) | (50, 105, 131, 153) |
| Dementia (n=6, 5%) | 1 (4%) | (143) | 0 (0%) |  | 5 (11%) | (50, 52, 87, 102, 120) |
| Depression (n=1, 1%) | 0 (0%) |  | 0 (0%) |  | 1 (2%) | (55) |
| Diabetes (n=2, 2%) | 1 (4%) | (117) | 0 (0%) |  | 2 (4%) | (117, 133) |
| End-of-life patients (n=2, 2%) | 0 (0%) |  | 0 (0%) |  | 2 (4%) | (51, 63) |
| Epilepsy (n=1, 1%) | 1 (4%) | (140) | 0 (0%) |  | 0 (0%) |  |
| IDD (n=58, 46%) | 1 (4%) | (117) | 57 (100%) | (10, 11, 40-49, 65, 66, 70-81, 88, 92-98, 100, 101, 103, 104, 109, 113-115, 122-126, 128, 129, 134, 146-152) | 4 (9%) | (75, 103, 117, 125) |
| ADHD (n=7, 6%) | 0 (0%) |  | 7 (12%) | (46, 72, 78, 88, 95, 122, 124) | 0 (0%) |  |
| ASD (n=36, 29%) | 0 (0%) |  | 36 (63%) | (40-49, 65, 66, 70, 71, 73, 74, 76, 77, 79-81, 93, 96-98, 104, 109, 113, 115, 125, 126, 134, 146, 148, 151, 152) | 1 (2%) | (125) |
| Cerebral palsy (n=4, 3%) | 0 (0%) |  | 4 (7%) | (94, 100, 114, 123) | 0 (0%) |  |
| Down syndrome (n=1, 1%) | 0 (0%) |  | 1 (2%) | (92) | 0 (0%) |  |
| Fragile X syndrome (n=1, 1%) | 0 (0%) |  | 1 (2%) | (101) | 0 (0%) |  |
| Prader–Willi syndrome (n=1, 1%) | 0 (0%) |  | 1 (2%) | (150) | 0 (0%) |  |
| Other IDD (n=5, 4%) | 0 (0%) |  | 5 (9%) | (66, 75, 81, 103, 128) | 2 (4%) | (75, 103) |
| Medical treatments (n=4, 3%) | 0 (0%) |  | 0 (0%) |  | 4 (9%) | (58, 86, 130, 132) |
| Old age (n=9, 7%) | 2 (7%) | (136, 142) | 0 (0%) |  | 7 (15%) | (12, 52, 57, 60, 102, 116, 130) |
| Parkinson’s disease (n=1, 1%) | 1 (4%) | (144) | 0 (0%) |  | 0 (0%) |  |
| Physical disabilities (n=1, 1%) | 0 (0%) |  | 0 (0%) |  | 1 (2%) | (116) |
| SUD or OUD (n=2, 2%) | 1 (4%) | (38) | 0 (0%) |  | 1 (2%) | (55) |
| TBI (n=7, 6%) | 7 (26%) | (32-35, 111, 140, 145) | 0 (0%) |  | 0 (0%) |  |
| Medical condition not specified (n=8, 6%) | 6 (22%) | (7, 39, 68, 110, 118, 121) | 0 (0%) |  | 3 (6%) | (57, 61, 118) |
| Other (n=23, 18%) | 6 (22%) | (36, 37, 69, 138, 139, 141) | 6 (11%) | (11, 72, 76, 94, 114, 124) | 11 (23%) | (12, 56, 59, 82, 85, 86, 108, 116, 130, 133, 155) |

ADHD = attention-deficit/hyperactivity disorder; ASD = autism spectrum disorder; OUD = opioid use disorder; PTSD = posttraumatic stress disorder; SUD = substance use disorder; TBI = traumatic brain injury.

Note. Because some studies include more than one population, the sum of studies by population exceeds the total. Other IDD conditions include other intellectual disabilities, non-ASD developmental disorders, neurodevelopmental disorders, chronic neurologic disorders, and children with special healthcare needs. Other medical conditions include systemic lupus erythematosus, vaccination, surgical correction of hip instability or scoliosis, retinal blastoma, PTSD, Papanicolaou tests and mammograms, other special healthcare needs, chronic illnesses, disability, neurologic conditions, mental health conditions, hypertension, hearing loss, glaucoma, colonoscopy, hematuria, cirrhosis, annual out-of-pocket expenses for the child’s health care, serious chronic conditions, and injury.

# Supplement D: Geography of data collection by population (N=126)

| Data source geography (n, % of all studies) | Veteran | | Intellectual and developmental disorders (IDD) | | Rural | |
| --- | --- | --- | --- | --- | --- | --- |
|  | (n=27, % of veteran studies) | Relevant citations | (n=57, % of IDD studies) | Relevant citations | (n=47, % of rural studies) | Relevant citations |
| National (n=64, 51%) | 13 (48%) | (7, 34, 35, 38, 39, 110, 111, 117, 118, 121, 136, 141, 145) | 42 (74%) | (10, 11, 40-49, 65, 66, 71-74, 76-81, 88, 93, 94, 96-98, 101, 113, 115, 124, 126, 128, 129, 146, 148, 150-152) | 11 (23%) | (52, 54, 55, 57, 60, 82, 99, 102, 117-119) |
| Single institution/site (n=23, 18%) | 8 (30%) | (32, 68, 112, 135, 138, 139, 143, 144) | 4 (7%) | (100, 103, 114, 123) | 12 (26%) | (50, 53, 58, 83, 86, 90, 103, 105, 106, 137, 153, 154) |
| Multiple sites in a single state (n=16, 13%) | 0 (0%) |  | 5 (9%) | (70, 75, 95, 125, 134) | 13 (28%) | (12, 56, 59, 61, 75, 85, 89, 91, 108, 120, 125, 130, 132) |
| Multiple sites across multiple states (n=17, 13%) | 2 (7%) | (36, 69) | 4 (7%) | (92, 109, 122, 147) | 11 (23%) | (51, 62, 63, 84, 87, 107, 116, 127, 131, 133, 155) |
| Not stated/unclear (n=6, 5%) | 4 (15%) | (33, 37, 140, 142) | 2 (4%) | (104, 149) | 0 (0%) |  |

Note. Because some studies include more than one population, the sum of studies by population exceeds the total.

# Supplement E: Abstraction Tables

| **Yoon, J., Chow, A., Jiang, H., Wong, E., & Chang, E. T. (2025). Comparing quality, costs, and outcomes of VA and community primary care for patients with diabetes. *Journal of General Internal Medicine*, *40*(3), 647–653.** | | |
| --- | --- | --- |
| Study Characteristics | Population(s) of Interest | Rural, veteran |
|  | Study Population and Sample Size | 656,298 patients |
|  | Medical Condition(s) | Diabetes, intellectual and developmental disabilities |
|  | Study Purpose | To compare quality, costs, and outcomes of community and Veterans’ Affairs–provided primary care for patients with diabetes over a 12-month episode. |
|  | Population(s) of Interest Are Focus of the Research | Yes |
|  | PCBEOs Are a Focus of the Research | No |
|  | Data Collection Geography | National |
|  | Data Source(s) | Medical records/electronic health records, medical claims |
| Findings | PCBEO(s) Studied |  |
|  | Indirect impacts | Time spent traveling to or between medical appointments |
|  | Population-Specific Justification Included | No |

| **Samuel, P. S., Marsack-Topolewski, C. N., & Chan, K. T. (2025). Quality of life of family caregivers of adults with autism: Role of caregiver burden, health, and social support of compound and noncompound caregivers. *Families in Society: Journal of Contemporary Social Services*.** | | |
| --- | --- | --- |
| Study Characteristics | Population(s) of Interest | Intellectual and developmental disabilities |
|  | Study Population and Sample Size | 249 caregivers |
|  | Medical Condition(s) | Autism spectrum disorder |
|  | Study Purpose | To examine the influence of perceived caregiver burden and social support while controlling for caregivers’ personal characteristics (self-reported health, annual family income) on quality of life for these three types of caregivers. |
|  | Population(s) of Interest Are Focus of the Research | Yes |
|  | PCBEOs Are a Focus of the Research | Yes |
|  | Data Collection Geography | National |
|  | Data Source(s) | Primary survey data |
| Findings | PCBEO(s) Studied |  |
|  | Indirect impacts | Time spent providing unpaid caregiving |
|  | Intangible burdens | Distress, stress, and anxiety directly caused by OOP costs, financial debt, or lost income (i.e., financial toxicity) |
|  | Population-Specific Justification Included | Yes |
|  | Justification(s) | Need factor(s): Disability status; gaps in the literature |

| **Owens, J. A., Bush, A., Campbell, P., Ward, R. J., & Lilly, F. R. W. (2025). Community-engaged insights: Understanding the housing needs of rural caregivers during hospital treatment. *Public Health Nursing*, *42*(2), 981–985.** | | |
| --- | --- | --- |
| Study Characteristics | Population(s) of Interest | Rural |
|  | Study Population and Sample Size | 5 caregivers |
|  | Medical Condition(s) | Medical treatments |
|  | Study Purpose | To explore the experiences of rural caregivers who require temporary housing during hospital treatment when healthcare is not available in their home communities. |
|  | Population(s) of Interest Are Focus of the Research | Yes |
|  | PCBEOs Are a Focus of the Research | Yes |
|  | Data Collection Geography | Single institution/site |
|  | Data Source(s) | Interviews |
| Findings | PCBEO(s) Studied |  |
|  | Direct non-medical costs | Transportation, meals, hoteling |
|  | Population-Specific Justification Included | Yes |
|  | Justification(s) | Enabling factor(s): Rurality, distance to the nearest medical institution; gaps in the literature |

| **Marsack-Topolewski, C. N., Wang, F., & Samuel, P. S. (2025). Characteristics of adult children with autism and caregiver burden. *Family Relations*, *74*(2), 931–950.** | | | |
| --- | --- | --- | --- |
| Study Characteristics | Population(s) of Interest | Intellectual and developmental disabilities | |
|  | Study Population and Sample Size | 320 caregivers | |
|  | Medical Condition(s) | Autism spectrum disorder (ASD) | |
|  | Study Purpose | To determine whether parental perceptions of their adult children’s communication, behavior, and socialization skills were significantly associated with caregiver burden after controlling for income and age of the child with an ASD. | |
|  | Population(s) of Interest Are Focus of the Research | Yes | |
|  | PCBEOs Are a Focus of the Research | Yes | |
|  | Data Collection Geography | National | |
|  | Data Source(s) | Primary survey data | |
| Findings | PCBEO(s) Studied |  | |
|  | Indirect impacts | Time spent providing unpaid caregiving, unpaid time off work, early retirement | |
|  | Intangible burdens | Diminished household wealth; family/social life impacts; distress, stress, and anxiety directly caused by OOP costs, financial debt, or lost income (i.e., financial toxicity); general or composite measure of caregiver burden | |
|  | Population-Specific Justification Included | Yes | |
|  | Justification(s) | Enabling factor(s): Income; need factor(s): Disability status | |
| **Grosvenor, L. P., Cohen, R. J., Gordon, N. P., Massolo, M. L., Cerros, H. J., Yoshida, C. K., Ames, J. L., & Croen, L. A. (2025). Barriers to healthcare for Latinx autistic children and adolescents. *Journal of Autism and Developmental Disorders*, *55*(2), 605–619.** | | |  |
| Study Characteristics | Population(s) of Interest | Intellectual and developmental disorders |  |
|  | Study Population and Sample Size | 417 caregivers |  |
|  | Medical Condition(s) | Autism spectrum disorder |  |
|  | Study Purpose | To understand the ways in which autistic Latinx children experience disparities in diagnosis, healthcare, and receipt of specialty services. |  |
|  | Population(s) of Interest Are Focus of the Research | Yes |  |
|  | PCBEOs Are a Focus of the Research | No |  |
|  | Data Collection Geography | Multiple sites in a single state |  |
|  | Data Source(s) | Primary survey data |  |
| Findings | PCBEO(s) Studied |  |  |
|  | Direct medical costs | Copayments, coinsurance, deductibles, premiums |  |
|  | Direct non-medical costs | Transportation, meals, hoteling |  |
|  | Population-Specific Justification Included | Yes |  |
|  | Justification(s) | Gaps in the literature |  |

| **Eberly, L., Tennison, A., Morgan, L., Smith, M., Gray, L., Kearney, M., Feliciano, B., Lindsey, E., Manche, J., Detsoi-Smiley, P., Shin, S., & Merino, M. (2025). Centering Diné (Navajo) voices: Barriers, facilitators, and perceptions of cardiac care among patients with heart failure in rural Navajo Nation. *Circulation*, *152*(2), 101–112.** | | |
| --- | --- | --- |
| Study Characteristics | Population(s) of Interest | Rural |
|  | Study Population and Sample Size | 30 patients |
|  | Medical Condition(s) | Heart failure (HF) |
|  | Study Purpose | To characterize the barriers, facilitators, and perceptions of cardiac care among Diné (Navajo) patients with HF receiving care through the Indian Health Service, as well as to determine patient-designed solutions to improve access to quality cardiovascular care. |
|  | Population(s) of Interest Are Focus of the Research | Yes |
|  | PCBEOs Are a Focus of the Research | Yes |
|  | Data Collection Geography | Single institution/site |
|  | Data Source(s) | Interviews |
| Findings | PCBEO(s) Studied |  |
|  | Direct non-medical costs | Transportation, meals, hoteling |
|  | Indirect impacts | Time spent traveling to or between medical appointments |
|  | Population-Specific Justification Included | Yes |
|  | Justification(s) | Enabling factor(s): Rurality; gaps in the literature |

| **Chow, K., Rezvan, P. H., Kazerooni, L., Nguyen, L., Boyd, N. K., Vogel, B. N., Lucas, M. C., Brown, R., Quinn, E. A., Jafarpour, S., & Santoro, J. D. (2025). Caregiver burden and familial impact in Down syndrome regression disorder. *Orphanet Journal of Rare Diseases*, *20*(1), 126.** | | |
| --- | --- | --- |
| Study Characteristics | Population(s) of Interest | Intellectual and developmental disabilities |
|  | Study Population and Sample Size | 365 caregivers |
|  | Medical Condition(s) | Down syndrome |
|  | Study Purpose | To elucidate the multifaceted impacts of Down syndrome regression disorder on caregivers and families. |
|  | Population(s) of Interest Are Focus of the Research | Yes |
|  | PCBEOs Are a Focus of the Research | Yes |
|  | Data Collection Geography | Multiple sites across multiple states |
|  | Data Source(s) | Primary survey data |
| Findings | PCBEO(s) Studied |  |
|  | Direct medical costs | General financial burden |
|  | Direct non-medical costs | Moving costs to be closer to medical care |
|  | Indirect impacts | Unpaid time off work, early retirement |
|  | Intangible burdens | Family/social life impacts, hindered career or education advancement |
|  | Population-Specific Justification Included | Yes |
|  | Justification(s) | Predisposing factors; enabling factor(s): Societal stigma; need factor(s): Disability status |

| **Benedict, C., Bloomer, K., Billman, E., Smith, M., Boynton, H., Schapira, L., & Smith, S. M. (2025). Identifying the financial toxicity experiences of childhood cancer survivors through partnership with a community organization serving rural and minoritized families. *Psychooncology*, *34*(3), e70120.** | | |
| --- | --- | --- |
| Study Characteristics | Population(s) of Interest | Rural |
|  | Study Population and Sample Size | 12 patients, 11 caregivers |
|  | Medical Condition(s) | Cancer |
|  | Study Purpose | To explore the financial toxicity (FT) experiences of adolescent/young adult survivors of childhood cancer and their parents and the perspective of staff members of a community‐based cancer support organization in a rural region with majority Hispanic/Latino residents and high rates of non‐English language preference. |
|  | Population(s) of Interest Are Focus of the Research | Yes |
|  | PCBEOs Are a Focus of the Research | Yes |
|  | Data Collection Geography | Single institution/site |
|  | Data Source(s) | Interviews |
| Findings | PCBEO(s) Studied |  |
|  | Direct non-medical costs | Transportation, meals, hoteling |
|  | Indirect impacts | Time spent traveling to or between medical appointments |
|  | Intangible burdens | Delaying or forgoing medical treatments due to costs; diminished household wealth; changes in employment status and/or insurance coverage; food insecurity; housing insecurity; reduced spending on basic goods, groceries, and leisure activities; distress, stress, and anxiety directly caused by OOP costs, financial debt, or lost income (i.e., FT) |
|  | Population-specific Justification Included | Yes |
|  | Justification(s) | Enabling factor(s): Rurality, partner and social support or total help received from relatives |

| **Bellnier, L., Mahairas, A., Oberman, M. K., Board, R., Pattabiraman, M., Heltsley, C., Ranseen, E., Shinn, J., & Bush, M. L. (2025). Prioritizing hearing: Patient and provider perspectives on rural hearing health care and patient navigation. *Journal of Speech, Language, and Hearing Research*, *68*(4), 2127–2143.** | | |
| --- | --- | --- |
| Study Characteristics | Population(s) of Interest | Rural |
|  | Study Population and Sample Size | 21 patients |
|  | Medical Condition(s) | Hearing loss |
|  | Study Purpose | To explore experiences of hearing loss, identify factors that impact access, and assess the potential acceptability of a patient navigation program. |
|  | Population(s) of Interest Are Focus of the Research | Yes |
|  | PCBEOs Are a Focus of the Research | No |
|  | Data Collection Geography | Multiple sites in a single state |
|  | Data Source(s) | Interviews |
| Findings | PCBEO(s) Studied |  |
|  | Direct medical costs | Copayments, coinsurance, deductibles, premiums, diagnosis/treatment/management costs (beyond cost sharing), personal medical equipment or consumables costs |
|  | Direct non-medical costs | Transportation, meals, hoteling |
|  | Intangible burdens | Delaying or forgoing medical treatments due to costs |
|  | Population-Specific Justification Included | Yes |
|  | Justification(s) | Enabling factor(s): Rurality; need factor(s): Higher incidence of hearing loss in rural populations |

| **Wagle, S., Yang, S., Osei, E. A., Katare, B., & Lalani, N. (2024). Caregiving intensity, duration, and subjective financial well-being among rural informal caregivers of older adults with chronic illnesses or disabilities. *Healthcare (Basel)*, *12*(22).** | | |
| --- | --- | --- |
| Study Characteristics | Population(s) of Interest | Rural |
|  | Study Population and Sample Size | 196 caregivers |
|  | Medical Condition(s) | Old age, physical disabilities, chronic illnesses or disabilities in older adults |
|  | Study Purpose | To understand how caregiving demands, intensity, and duration impact the subjective financial well-being of rural caregivers of older adults. |
|  | Population(s) of Interest Are Focus of the Research | Yes |
|  | PCBEOs Are a Focus of the Research | Yes |
|  | Data Collection Geography | Multiple sites across multiple states |
|  | Data Source(s) | Primary survey data |
| Findings | PCBEO(s) Studied |  |
|  | Indirect impacts | Time spent providing unpaid caregiving |
|  | Intangible burdens | Distress, stress, and anxiety directly caused by OOP costs, financial debt, or lost income (i.e., financial toxicity) |
|  | Population-Specific Justification Included | Yes |
|  | Justification(s) | Enabling factor(s): Rurality, financial insecurity; policy factors |

| **Shepherd-Banigan, M., Cannedy, S., Rodriguez, A., Burns, M., Woolson, S., Hamilton, A., Quiroz, I., Matthews, H., Garber-Cardwell, D., Byrd, K. G., Brown, A., & Goldstein, K. M. (2024). Veteran caretaker perspectives of the need for childcare assistance during health care appointments. *Women’s Health Issues*, *34*(1), 98–106.** | | |
| --- | --- | --- |
| Study Characteristics | Population(s) of Interest | Veteran |
|  | Study Population and Sample Size | 2,000 Caregivers |
|  | Medical Condition(s) | Medical condition not specified |
|  | Study Purpose | To describe the childcare needs of veteran caretakers of young children and explores the implications of inadequate childcare on health care engagement. |
|  | Population(s) of interest are focus of the research | Yes |
|  | PCBEOs are a focus of the research | No |
|  | Data Collection Geography | National |
|  | Data Source(s) | Primary survey data, Focus groups |
| Findings | PCBEO(s) Studied |  |
|  | Direct non-medical costs | Child/dependent care costs |
|  | Population-specific Justification Included | Yes |
|  | Justification(s) | Need factor(s): Chronic illnesses; Policy factors |

| **Scodari, B. T., Schaefer, A. P., Kapadia, N. S., O’Malley, A. J., Brooks, G. A., Tosteson, A. N. A., Onega, T., Wang, C., Wang, F., & Moen, E. L. (2024). Characterizing the traveling oncology workforce and its influence on patient travel burden: A claims-based approach. *JCO Oncology Practice*, *20*(6), 787–796.** | | |
| --- | --- | --- |
| Study Characteristics | Population(s) of Interest | Rural |
|  | Study Population and Sample Size | 61,704 Patients |
|  | Medical Condition(s) | Breast cancer, Colorectal cancer, Lung cancer |
|  | Study Purpose | To develop a claims-based approach for identifying traveling oncologists, characterize the at tributes of the traveling oncology workforce, and investigate the influence of oncology outreach on rural patient travel time to chemotherapy, radiotherapy, and surgery. |
|  | Population(s) of interest are focus of the research | Yes |
|  | PCBEOs are a focus of the research | Yes |
|  | Data Collection Geography | National |
|  | Data Source(s) | Medical claims |
| Findings | PCBEO(s) Studied |  |
|  | Indirect impacts | Time spent traveling to or between medical appointments |
|  | Population-specific Justification Included | Yes |
|  | Justification(s) | Need factors; Gaps in the literature |

| **Ramchand, R., Dalton, S., Dubowitz, T., Hyde, K., Malika, N., Morral, A. R., Ohana, E., Parks, V., Schell, T. L., Swabe, G., Trail, T. E., & Williams, K. M. (2024). America’s military and veteran caregivers: Hidden heroes emerging from the shadows. *RAND Health Quarterly*, *12*(1), 7.** | | |
| --- | --- | --- |
| Study Characteristics | Population(s) of Interest | Veteran |
|  | Study Population and Sample Size | 1,100 Patients, 1,818 Caregivers (1,613 Veteran) |
|  | Medical Condition(s) | Medical condition not specified |
|  | Study Purpose | To estimate the number of adults caregiving in the United States today; investigate how those caring for wounded, ill, and injured service members and veterans compare with those caring for civilians and with non-caregivers; and share insights on the potential consequences of caregiving on caregivers' health, their economic security, and their families' well-being. |
|  | Population(s) of interest are focus of the research | Yes |
|  | PCBEOs are a focus of the research | No |
|  | Data Collection Geography | National |
|  | Data Source(s) | Secondary survey data |
| Findings | PCBEO(s) Studied |  |
|  | Direct medical costs | Copayments, coinsurance, deductibles, premiums |
|  | Indirect impacts | Time spent providing unpaid caregiving, Time spent traveling to or between medical appointments, Unpaid time off work, early retirement |
|  | Intangible burdens | General or composite measure of caregiver burden |
|  | Population-specific Justification Included | Yes |
|  | Justification(s) | Enabling factor(s): Medical insurance; Gaps in the literature; Policy factors |

| **Piamjariyakul, U., Wang, K., Smith, M., Young, S., Shafique, S., Navia, R. O., & Williams, K. (2024). Family caregiving of patients with heart failure and vascular dementia in rural Appalachia: A mixed-methods study. *Western Journal of Nursing Research*, *46*(5), 344–355.** | | |
| --- | --- | --- |
| Study Characteristics | Population(s) of Interest | Rural |
|  | Study Population and Sample Size | 20 Caregivers |
|  | Medical Condition(s) | Dementia, HF |
|  | Study Purpose | To assess caregivers’ burden and their physical and mental health status, as well as explore their experiences and needs. |
|  | Population(s) of interest are focus of the research | Yes |
|  | PCBEOs are a focus of the research | Yes |
|  | Data Collection Geography | Single institution/site |
|  | Data Source(s) | Primary survey data, Focus groups, Interviews |
| Findings | PCBEO(s) Studied |  |
|  | Intangible burdens | General or composite measure of caregiver burden |
|  | Population-specific Justification Included | Yes |
|  | Justification(s) | Predisposing factor(s): Lack of trust; Enabling factor(s): Medical insurance, Rurality, Partner and social support or total help received from relatives; Gaps in the literature |

| **Piamjariyakul, U., Smothers, A., Wang, K., Shafique, S., Wen, S., Petitte, T., Young, S., Sokos, G., & Smith, C. E. (2024). Palliative care for patients with heart failure and family caregivers in rural Appalachia: A randomized controlled trial. *BMC Palliative Care*, *23*(1), 1–14.** | | |
| --- | --- | --- |
| Study Characteristics | Population(s) of Interest | Rural |
|  | Study Population and Sample Size | 39 Caregivers |
|  | Medical Condition(s) | HF |
|  | Study Purpose | To test whether the family HF palliative and end-of-life care intervention (FamPALcare) improved patient and caregiver outcomes at 3- and 6-month study endpoints. |
|  | Population(s) of interest are focus of the research | Yes |
|  | PCBEOs are a focus of the research | Yes |
|  | Data Collection Geography | Single institution/site |
|  | Data Source(s) | Primary survey data |
| Findings | PCBEO(s) Studied |  |
|  | Intangible burdens | General or composite measure of caregiver burden |
|  | Population-specific Justification Included | Yes |
|  | Justification(s) | Enabling factor(s): Rurality, Distance to the nearest medical institution |

| **Nah, S., Savla, J., & Roberto, K. A. (2024). Dementia care in rural Appalachia: Multilevel analysis of individual- and county-level factors. *Gerontologist*, *64*(7).** | | |
| --- | --- | --- |
| Study Characteristics | Population(s) of Interest | Rural |
|  | Study Population and Sample Size | 123 Caregivers |
|  | Medical Condition(s) | Dementia |
|  | Study Purpose | To examine individual- and county-level factors influencing home- and community-based services (HCBS) utilization for dementia care in rural Appalachia. |
|  | Population(s) of interest are focus of the research | Yes |
|  | PCBEOs are a focus of the research | No |
|  | Data Collection Geography | Multiple sites in a single state |
|  | Data Source(s) | Primary survey data |
| Findings | PCBEO(s) Studied |  |
|  | Indirect impacts | Time spent traveling to or between medical appointments |
|  | Intangible burdens | General or composite measure of caregiver burden |
|  | Population-specific Justification Included | No |

| **Miller, M. F., Olson, J. S., Doughtie, K., Zaleta, A. K., & Rogers, K. P. (2024). The interplay of financial toxicity, health care team communication, and psychosocial well-being among rural cancer patients and survivors. *Journal of Rural Health*, *40*(1), 128–137.** | | |
| --- | --- | --- |
| Study Characteristics | Population(s) of Interest | Rural |
|  | Study Population and Sample Size | 273 Patients |
|  | Medical Condition(s) | Cancer |
|  | Study Purpose | To examine associations between financial toxicity and psychosocial well-being among rural cancer patients, exploring variability in these linkages by health care team communication. |
|  | Population(s) of interest are focus of the research | Yes |
|  | PCBEOs are a focus of the research | Yes |
|  | Data Collection Geography | National |
|  | Data Source(s) | Primary survey data |
| Findings | PCBEO(s) Studied |  |
|  | Direct medical costs | Composite OOP costs |
|  | Intangible burdens | Distress, stress, and anxiety directly caused by OOP costs, financial debt, or lost income (i.e., financial toxicity) |
|  | Population-specific Justification Included | Yes |
|  | Justification(s) | Enabling factor(s): Rurality |

| **Mastrogiannis, A. M., Steinway, C., Santos, T. C., Chen, J., Berens, J., Davis, T., Cornacchia, M., Woodward, J., Riddle, I., Spicer, B., Wright, C., Lindquist, L. A., & Jan, S. (2024). Medicaid long-term services and supports and caregiving needs of caregivers of individuals with intellectual and developmental disabilities. *Journal of Applied Research in Intellectual Disabilities*, *37*(5), e13289.** | | |
| --- | --- | --- |
| Study Characteristics | Population(s) of Interest | IDD |
|  | Study Population and Sample Size | 405 Caregivers |
|  | Medical Condition(s) | ASD |
|  | Study Purpose | To identify the different caregiving challenges and stressors among caregivers of those with intellectual disabilities through direct report by caregivers (Aim 1). Additionally, we will examine the associations between these challenging aspects of caregiving among different subgroups of caregivers (Aim 2) and the presence of Medicaid waivers (Aim 3). |
|  | Population(s) of interest are focus of the research | Yes |
|  | PCBEOs are a focus of the research | Yes |
|  | Data Collection Geography | Multiple sites across multiple states |
|  | Data Source(s) | Primary survey data |
| Findings | PCBEO(s) Studied |  |
|  | Indirect impacts | Increased time required for daily tasks, Time spent navigating insurance or billing, Time spent traveling to or between medical appointments |
|  | Intangible burdens | General or composite measure of caregiver burden |
|  | Population-specific Justification Included | Yes |
|  | Justification(s) | Need factors; Policy factors |

| **Marsack-Topolewski, C. N., & McGinley, J. M. (2024). Comparing caregiving outcomes among male and female parents of autistic adults. *Families in Society: The Journal of Contemporary Social Services*, *106*(3), 911–925.** | | |
| --- | --- | --- |
| Study Characteristics | Population(s) of Interest | IDD |
|  | Study Population and Sample Size | 114 Caregivers |
|  | Medical Condition(s) | ASD |
|  | Study Purpose | To compare aging male and female caregivers of autistic adults to determine if self-reported caregiver health, QOL, caregiver burden, informal social support, and formal social support differed by gender. |
|  | Population(s) of interest are focus of the research | Yes |
|  | PCBEOs are a focus of the research | Yes |
|  | Data Collection Geography | National |
|  | Data Source(s) | Primary survey data |
| Findings | PCBEO(s) Studied |  |
|  | Intangible burdens | General or composite measure of caregiver burden, General financial burden |
|  | Population-specific Justification Included | Yes |
|  | Justification(s) | Enabling factor(s): general service landscape; Need factor(s): Disability status; Gaps in the literature |

| **Marsack-Topolewski, C. N. (2024). Comparison of caregiver burden and quality of life between compound caregivers and those with one adult child with autism. *International Journal of Disability Development and Education*, *72*(4), 646–660.** | | |
| --- | --- | --- |
| Study Characteristics | Population(s) of Interest | IDD |
|  | Study Population and Sample Size | 320 Caregivers |
|  | Medical Condition(s) | ASD |
|  | Study Purpose | To examine differences in caregiver burden and quality of life between compound caregivers (those who are providing care for more than one person, often with different disabilities) and noncompound caregivers (those caring for a single adult with ASD). |
|  | Population(s) of interest are focus of the research | Yes |
|  | PCBEOs are a focus of the research | Yes |
|  | Data Collection Geography | National |
|  | Data Source(s) | Primary survey data |
| Findings | PCBEO(s) Studied |  |
|  | Indirect impacts | Time spent providing unpaid caregiving |
|  | Intangible burdens | General or composite measure of caregiver burden, General financial burden |
|  | Population-specific Justification Included | Yes |
|  | Justification(s) | Need factor(s): Disability status; Gaps in the literature |

| **Lalani, N., Hamash, K., & Wang, Y. (2024). Palliative care needs and preferences of older adults with advanced or serious chronic illnesses and their families in rural communities of Indiana, USA. *Journal of Rural Health*, *40*(2), 368–375.** | | |
| --- | --- | --- |
| Study Characteristics | Population(s) of Interest | Rural |
|  | Study Population and Sample Size | 15 Caregivers |
|  | Medical Condition(s) | Old age, Advanced chronic conditions |
|  | Study Purpose | To explore the palliative care needs and preferences of older adults with advanced or serious chronic illnesses and their families. Also, to propose strategies to promote supportive palliative care in the rural communities of Indiana, USA. |
|  | Population(s) of interest are focus of the research | Yes |
|  | PCBEOs are a focus of the research | Yes |
|  | Data Collection Geography | Multiple sites in a single state |
|  | Data Source(s) | Interviews |
| Findings | PCBEO(s) Studied |  |
|  | Indirect impacts | Time to attend medical appointments, Time spent traveling to or between medical appointments, Unpaid time off work, Early retirement |
|  | Intangible burdens | Delaying or forgoing medical treatments due to costs, Changes in employment status and/or insurance coverage, Distress, stress, and anxiety directly caused by OOP costs, financial debt, or lost income (i.e., financial toxicity), Reduced self-care |
|  | Population-specific Justification Included | Yes |
|  | Justification(s) | Predisposing factors; Enabling factor(s): Income, Rurality, Inadequate healthcare workforce, discrimination; Need factor(s): Chronic Illnesses |

| **Kazeem, A. O., Hasken, W., Sims, T., Culp, S. H., Krupski, T. L., & Lobo, J. M. (2024). Patient satisfaction with a novel tele-cystoscopy model: Expanding access to bladder cancer surveillance for rural patients. *Telemedicine Reports*, *5*(1), 229–236.** | | |
| --- | --- | --- |
| Study Characteristics | Population(s) of Interest | Rural |
|  | Study Population and Sample Size | 48 Patients |
|  | Medical Condition(s) | Bladder cancer |
|  | Study Purpose | To expand the availability of cystoscopy to underserved rural areas. |
|  | Population(s) of interest are focus of the research | Yes |
|  | PCBEOs are a focus of the research | No |
|  | Data Collection Geography | Multiple sites across multiple states |
|  | Data Source(s) | Primary survey data |
| Findings | PCBEO(s) Studied |  |
|  | Direct non-medical costs | Transportation, meals, hoteling |
|  | Indirect impacts | Time spent traveling to or between medical appointments, Unpaid time off work, early retirement |
|  | Population-specific Justification Included | Yes |
|  | Justification(s) | Enabling factor(s): Rurality |

| **Kaufman, B. G., Zhang, W., Shibeika, S., Huang, R. W., Xu, T., Ingram, C., Gustavson, A. M., Holland, D. E., Vanderboom, C., Van Houtven, C. H., & Griffin, J. M. (2024). Economic value of unpaid family caregiver time following hospital discharge and at end of life. *Journal of Pain and Symptom Management*, *68*(6), 632–640.** | | |
| --- | --- | --- |
| Study Characteristics | Population(s) of Interest | Rural |
|  | Study Population and Sample Size | 282 Caregivers |
|  | Medical Condition(s) | End-of-life patients |
|  | Study Purpose | To quantify the economic value of unpaid caregiving by rural FCG using two commonly used valuation methods as well as a combination approach. |
|  | Population(s) of interest are focus of the research | Yes |
|  | PCBEOs are a focus of the research | Yes |
|  | Data Collection Geography | Multiple sites across multiple states |
|  | Data Source(s) | Primary survey data |
| Findings | PCBEO(s) Studied |  |
|  | Indirect impacts | Time spent providing unpaid caregiving |
|  | Intangible burdens | Changes in employment status and/or insurance coverage |
|  | Population-specific Justification Included | Yes |
|  | Justification(s) | Enabling factor(s): Rurality, Distance to the nearest medical institution; Gaps in the literature |

| **Kaufman, B. G., Huang, R. W., Holland, D. E., Vanderboom, C. E., Ingram, C., Wild, E. M., Dose, A. M., Stiles, C., Gustavson, A. M., Mandrekar, J., Van Houtven, C. H., & Griffin, J. M. (2024). Healthcare use and out-of-pocket costs for rural family caregivers and care recipients in a randomized controlled trial. *Journal of the American Geriatrics Society*, *72*(8), 2523–2531.** | | |
| --- | --- | --- |
| Study Characteristics | Population(s) of Interest | Rural |
|  | Study Population and Sample Size | 282 Patients, 282 Caregivers |
|  | Medical Condition(s) | End-of-life patients |
|  | Study Purpose | To evaluate the TPC's effect on healthcare use and OOP spending for both Rural family caregivers (FCGs) and care recipients (CRs). |
|  | Population(s) of interest are focus of the research | Yes |
|  | PCBEOs are a focus of the research | Yes |
|  | Data Collection Geography | Multiple sites across multiple states |
|  | Data Source(s) | Primary survey data |
| Findings | PCBEO(s) Studied |  |
|  | Direct medical costs | Copayments, coinsurance, deductibles, premiums, Diagnosis/treatment/management costs (beyond cost sharing), Medication costs, Personal medical equipment or consumables costs |
|  | Direct non-medical costs | Transportation, meals, hoteling |
|  | Population-specific Justification Included | Yes |
|  | Justification(s) | Predisposing factor(s): cultural differences of rural caregivers; Enabling factor(s): Rurality, Distance to the nearest medical institution; Gaps in the literature |

| **Imanpour, S., & Ullah, R. (2024). Barriers and mediators for routine dental care visits from the experiences of immigrant parents of autistic children living in the United States. *Special Care in Dentistry*, *44*(4), 1253–1259.** | | |
| --- | --- | --- |
| Study Characteristics | Population(s) of Interest | IDD |
|  | Study Population and Sample Size | 19 Caregivers |
|  | Medical Condition(s) | ASD |
|  | Study Purpose | To describe the experiences of immigrant parents with autistic children and the barriers and mediators to routine dental care visits for their children in the United States. |
|  | Population(s) of interest are focus of the research | Yes |
|  | PCBEOs are a focus of the research | No |
|  | Data Collection Geography | National |
|  | Data Source(s) | Interviews |
| Findings | PCBEO(s) Studied |  |
|  | Direct medical costs | Copayments, coinsurance, deductibles, premiums |
|  | Direct non-medical costs | Transportation, meals, hoteling |
|  | Population-specific Justification Included | Yes |
|  | Justification(s) | Need factors; Gaps in the literature |

| **Hallinan, S., Sobering, J., Cardona-Martell, I., Girardin, M., Gohagon, K., O’Leary, D., & Stewart, G. (2024). Care recipient factors that predict caregiver burden in older adult dyads. *Home Health Care Management & Practice*, *36*(3), 191–196.** | | |
| --- | --- | --- |
| Study Characteristics | Population(s) of Interest | Veteran |
|  | Study Population and Sample Size | 201 Caregivers |
|  | Medical Condition(s) | Medical conditions requiring extensive follow-up that also made it difficult or impossible to attend in-person office visits |
|  | Study Purpose | To identify factors that impact caregiver burden within caregiver dyads enrolled in one of the VA's HBPC program teams to help facilitate effective support for the caregiver. |
|  | Population(s) of interest are focus of the research | Yes |
|  | PCBEOs are a focus of the research | Yes |
|  | Data Collection Geography | Single institution/site |
|  | Data Source(s) | Medical records/EHRs |
| Findings | PCBEO(s) Studied |  |
|  | Intangible burdens | General or composite measure of caregiver burden |
|  | Population-specific Justification Included | No |

| **Gutiérrez, Ã., Lopez-Anuarbe, M., Webster, N. J., & Mahmoudi, E. (2024). Rural-urban health care cost differences among Latinx adults with and without dementia in the United States. *Journal of Aging and Health*, *36*(9), 559–569.** | | |
| --- | --- | --- |
| Study Characteristics | Population(s) of Interest | Rural |
|  | Study Population and Sample Size | 16,213 Patients (1,946 Rural) |
|  | Medical Condition(s) | Dementia, Old age |
|  | Study Purpose | To compare rural–urban health care costs among Latinx adults ages 51+ and examine variations by dementia status. |
|  | Population(s) of interest are focus of the research | Yes |
|  | PCBEOs are a focus of the research | Yes |
|  | Data Collection Geography | National |
|  | Data Source(s) | Secondary survey data |
| Findings | PCBEO(s) Studied |  |
|  | Direct medical costs | Copayments, coinsurance, deductibles, premiums, Diagnosis/treatment/management costs (beyond cost sharing), Medication costs |
|  | Population-specific Justification Included | Yes |
|  | Justification(s) | Enabling factor(s): Rurality, Distance to the nearest medical institution; Need factor(s): Perceived general health status |

| **Fusco, K. N., Gonzalez Isoba, L. C., Alef, R., Roger, A., & Mayrovitz, H. N. (2024). Effects of demographic identities on psychosocial burdens of patients living in the United States with systemic lupus erythematosus. *Cureus*, *16*(12), e75043.** | | |
| --- | --- | --- |
| Study Characteristics | Population(s) of Interest | Rural |
|  | Study Population and Sample Size | 294 Patients |
|  | Medical Condition(s) | Systemic Lupus Erythematosus |
|  | Study Purpose | To examine how demographic factors such as age, race/ethnicity, and geographic location influence these burdens. |
|  | Population(s) of interest are focus of the research | Yes |
|  | PCBEOs are a focus of the research | Yes |
|  | Data Collection Geography | National |
|  | Data Source(s) | Primary survey data |
| Findings | PCBEO(s) Studied |  |
|  | Direct medical costs | Medication costs, Copayments, coinsurance, deductibles, premiums |
|  | Intangible burdens | Delaying or forgoing medical treatments due to costs |
|  | Population-specific Justification Included | No |

| **Fuller, H. R., Huseth-Zosel, A., Van Vleet, B., & Carson, P. J. (2024). Barriers to vaccination among older adults: Demographic variation and links to vaccine acceptance. *Aging and Health Research*, *4*(1), 7.** | | |
| --- | --- | --- |
| Study Characteristics | Population(s) of Interest | Rural |
|  | Study Population and Sample Size | 901 Patients (252 Rural) |
|  | Medical Condition(s) | Medical treatments, Old age, Vaccination |
|  | Study Purpose | To investigate possible barriers to acceptance of vaccines recommended for older adults and consider demographic variation and links to acceptance for distinct vaccines. |
|  | Population(s) of interest are focus of the research | No |
|  | PCBEOs are a focus of the research | No |
|  | Data Collection Geography | Multiple sites in a single state |
|  | Data Source(s) | Primary survey data |
| Findings | PCBEO(s) Studied |  |
|  | Intangible burdens | Delaying or forgoing medical treatments due to costs |
|  | Population-specific Justification Included | No |

| **Cohen, S. A., Ahmed, N. H., Ellis, K. A., Lindsey, H., Nash, C. C., & Greaney, M. L. (2024). Rural-urban and regional variations in aspects of caregiving, support services and caregiver health in the USA: Evidence from a national survey. *BMJ Open*, *14*(10), e081581.** | | |
| --- | --- | --- |
| Study Characteristics | Population(s) of Interest | Rural |
|  | Study Population and Sample Size | 3,551 Caregivers |
|  | Medical Condition(s) | Old age |
|  | Study Purpose | To determine whether rural-urban differences in caregiving intensity, caregiver burden, caregiver health and seeking caregiving support, vary by the US region after accounting for other social determinants of health. |
|  | Population(s) of interest are focus of the research | Yes |
|  | PCBEOs are a focus of the research | Yes |
|  | Data Collection Geography | National |
|  | Data Source(s) | Secondary survey data |
| Findings | PCBEO(s) Studied |  |
|  | Indirect impacts | Time spent providing unpaid caregiving |
|  | Intangible burdens | Distress, stress, and anxiety directly caused by OOP costs, financial debt, or lost income (i.e., financial toxicity) |
|  | Population-specific Justification Included | Yes |
|  | Justification(s) | Enabling factor(s): Rurality; Gaps in the literature |

| **Brunt, S., Nevill, R., & Mazurek, M. O. (2024). Associations among autism symptom domains and facets of caregiver strain. *Journal of Autism & Developmental Disorders*, *54*(4), 1507–1516.** | | |
| --- | --- | --- |
| Study Characteristics | Population(s) of Interest | IDD |
|  | Study Population and Sample Size | 450 Patients |
|  | Medical Condition(s) | ASD |
|  | Study Purpose | To investigate how differences in core autism symptomatology in children uniquely relate to caregiver strain. |
|  | Population(s) of interest are focus of the research | Yes |
|  | PCBEOs are a focus of the research | Yes |
|  | Data Collection Geography | National |
|  | Data Source(s) | Primary survey data |
| Findings | PCBEO(s) Studied |  |
|  | Intangible burdens | General or composite measure of caregiver burden |
|  | Population-specific Justification Included | Yes |
|  | Justification(s) | Gaps in the literature |

| **Vaitsiakhovich, N., & Landes, S. D. (2023). The association between the Patient Protection and Affordable Care Act and healthcare affordability among US adults with intellectual disability. *Journal of Intellectual Disability Research*, *67*(12), 1270–1290.** | | |
| --- | --- | --- |
| Study Characteristics | Population(s) of Interest | IDD |
|  | Study Population and Sample Size | 623 Patients |
|  | Medical Condition(s) | IDD |
|  | Study Purpose | This study aims to explore the association between healthcare affordability among persons with intellectual disability (ID) and the Patient Protection and Patient Protection and Affordable Care Act (ACA). |
|  | Population(s) of interest are focus of the research | Yes |
|  | PCBEOs are a focus of the research | Yes |
|  | Data Collection Geography | National |
|  | Data Source(s) | Secondary survey data |
| Findings | PCBEO(s) Studied |  |
|  | Intangible burdens | Delaying or forgoing medical treatments due to costs |
|  | Population-specific Justification Included | Yes |
|  | Justification(s) | Enabling factor(s): Income, Medical insurance; Need factor(s): Chronic illnesses, Disability status; Policy factors |

| **Shen, J., Shi, J., Gauthier, L., & Li, W. (2023). The economic burden of injuries in children with ADHD in the U.S. from 2011 to 2020. *Journal of Attention Disorders*, *27*(14), 1561–1570.** | | |
| --- | --- | --- |
| Study Characteristics | Population(s) of Interest | IDD |
|  | Study Population and Sample Size | 7,102 Patients |
|  | Medical Condition(s) | Injury, ADHD |
|  | Study Purpose | To examine the longitudinal trend of the overall injury-related economic burden and sociodemographic correlates of the economic burden of injuries at both family and societal levels among children with ADHD. |
|  | Population(s) of interest are focus of the research | Yes |
|  | PCBEOs are a focus of the research | Yes |
|  | Data Collection Geography | National |
|  | Data Source(s) | Secondary survey data |
| Findings | PCBEO(s) Studied |  |
|  | Direct medical costs | Copayments, coinsurance, deductibles, premiums, Diagnosis/treatment/management costs (beyond cost sharing) |
|  | Population-specific Justification Included | Yes |
|  | Justification(s) | Need factor(s): More injuries; Gaps in the literature |

| **Santos, T., Steinway, C., Mastrogiannis, A., Chen, J., Woodward, J., Riddle, I., Spicer, B., Berens, J., Davis, T., Cornacchia, M., Wright, C., Lindquist, L. A., & Jan, S. (2023). Caregiver support, burden, and long-term planning among caregivers of individuals with intellectual and developmental disabilities: A cross-sectional study. *Journal of Applied Research in Intellectual Disabilities*, *36*(6), 1229–1240.** | | |
| --- | --- | --- |
| Study Characteristics | Population(s) of Interest | IDD |
|  | Study Population and Sample Size | 405 Caregivers |
|  | Medical Condition(s) | IDD |
|  | Study Purpose | To investigate progression through LTCP, and associations between social support and: (1) LTCP and (2) burden among family caregivers. |
|  | Population(s) of interest are focus of the research | Yes |
|  | PCBEOs are a focus of the research | No |
|  | Data Collection Geography | Multiple sites across multiple states |
|  | Data Source(s) | Primary survey data |
| Findings | PCBEO(s) Studied |  |
|  | Intangible burdens | General or composite measure of caregiver burden |
|  | Population-specific Justification Included | Yes |
|  | Justification(s) | Enabling factors; Need factor(s): Disability status; Gaps in the literature; Policy factors |

| **Rattray, N. A., Flanagan, M., Mann, A., Danson, L., Do, A. N., Natividad, D., Spontak, K., & True, G. (2023). Conceptualizing care partners’ burden, stress, and support for reintegrating veterans: A mixed methods study. *Frontiers in Public Health*, *11*, 1295627.** | | |
| --- | --- | --- |
| Study Characteristics | Population(s) of Interest | Veteran |
|  | Study Population and Sample Size | 36 Caregivers |
|  | Medical Condition(s) | Mental health |
|  | Study Purpose | To identify key challenges faced by care partners of Veterans with invisible injuries. |
|  | Population(s) of interest are focus of the research | Yes |
|  | PCBEOs are a focus of the research | Yes |
|  | Data Collection Geography | Not stated/unclear |
|  | Data Source(s) | Interviews |
| Findings | PCBEO(s) Studied |  |
|  | Intangible burdens | General or composite measure of caregiver burden |
|  | Population-specific Justification Included | Yes |
|  | Justification(s) | Need factor(s): Higher burden in the population |

| **Ratnapradipa, K. L., Jadhav, S., Kabayundo, J., Wang, H., & Smith, L. C. (2023). Factors associated with delaying medical care: Cross-sectional study of Nebraska adults. *BMC Health Services Research*, *23*(1), 118.** | | |
| --- | --- | --- |
| Study Characteristics | Population(s) of Interest | Rural |
|  | Study Population and Sample Size | 1,101 Patients |
|  | Medical Condition(s) | Medical condition not specified |
|  | Study Purpose | To describe Nebraskans’ access to medical care and assess factors associated with ever delaying medical care, ever delaying specifically due to cost or transportation, and delaying medical care within the past 12 months of assessment. |
|  | Population(s) of interest are focus of the research | Yes |
|  | PCBEOs are a focus of the research | Yes |
|  | Data Collection Geography | Multiple sites in a single state |
|  | Data Source(s) | Primary survey data |
| Findings | PCBEO(s) Studied |  |
|  | Intangible burdens | Delaying or forgoing medical treatments due to costs |
|  | Population-specific Justification Included | Yes |
|  | Justification(s) | Enabling factor(s): Rurality |

| **Mazurek, M. O., Sadikova, E., Cheak-Zamora, N., Hardin, A., Sohl, K., & Malow, B. A. (2023). Health care needs, experiences, and perspectives of autistic adults. *Autism in Adulthood*, *5*(1), 51–62.** | | |
| --- | --- | --- |
| Study Characteristics | Population(s) of Interest | IDD |
|  | Study Population and Sample Size | 20 Patients |
|  | Medical Condition(s) | ASD |
|  | Study Purpose | To examine the perspectives of autistic adults regarding their health care experiences in primary care and other settings and their suggestions for improvement using both qualitative and quantitative methods. |
|  | Population(s) of interest are focus of the research | Yes |
|  | PCBEOs are a focus of the research | Yes |
|  | Data Collection Geography | National |
|  | Data Source(s) | Interviews |
| Findings | PCBEO(s) Studied |  |
|  | Direct medical costs | Copayments, coinsurance, deductibles, premiums |
|  | Direct non-medical costs | Transportation, meals, hoteling |
|  | Indirect impacts | Time to attend medical appointments |
|  | Population-specific Justification Included | Yes |
|  | Justification(s) | Enabling factor(s): Income, Difficulties with transportation; Gaps in the literature; Policy factors |

| **Mann, H. K., Streiff, M., Schultz, K. C., Halpern, D. V., Ferry, D., Johnson, A. E., & Magnani, J. W. (2023). Rurality and atrial fibrillation: Patient perceptions of barriers and facilitators to care. *Journal of the American Heart Association*, *12*(21), 9.** | | |
| --- | --- | --- |
| Study Characteristics | Population(s) of Interest | Rural |
|  | Study Population and Sample Size | 47 Patients |
|  | Medical Condition(s) | Atrial fibrillation |
|  | Study Purpose | To conduct focus groups of rural individuals with AF to better understand their experience of the condition and their perceptions of barriers and facilitators to their AF care. |
|  | Population(s) of interest are focus of the research | Yes |
|  | PCBEOs are a focus of the research | Yes |
|  | Data Collection Geography | Multiple sites in a single state |
|  | Data Source(s) | Focus groups |
| Findings | PCBEO(s) Studied |  |
|  | Direct medical costs | Medication costs |
|  | Indirect impacts | Time spent traveling to or between medical appointments |
|  | Population-specific Justification Included | Yes |
|  | Justification(s) | Enabling factor(s): Rurality; Need factor(s): Chronic illnesses; Gaps in the literature |

| **Kent, E. E., Lee, S., Asad, S., Dobbins, E. E., Aimone, E., & Park, E. M. (2023). “If I wasn’t in a rural area, I would definitely have more support”: Social needs identified by rural cancer caregivers and hospital staff. *Journal of Psychosocial Oncology*, *41*(4), 393–410.** | | |
| --- | --- | --- |
| Study Characteristics | Population(s) of Interest | Rural |
|  | Study Population and Sample Size | 24 Caregivers |
|  | Medical Condition(s) | Cancer |
|  | Study Purpose | To assess unmet social needs of family caregivers providing care to adults living with cancer in rural settings, from their own perspectives and that of clinicians and administrators working with them. |
|  | Population(s) of interest are focus of the research | Yes |
|  | PCBEOs are a focus of the research | Yes |
|  | Data Collection Geography | Single institution/site |
|  | Data Source(s) | Interviews |
| Findings | PCBEO(s) Studied |  |
|  | Direct non-medical costs | Transportation, meals, hoteling |
|  | Indirect impacts | Time to attend medical appointments, Unpaid time off work, early retirement |
|  | Intangible burdens | Distress, stress, and anxiety directly caused by OOP costs, financial debt, or lost income (i.e., financial toxicity) |
|  | Population-specific Justification Included | Yes |
|  | Justification(s) | Enabling factor(s): Rurality; Need factor(s): Higher cancer incidence; Gaps in the literature; Policy factors |

| **Ishler, K. J., Berg, K. A., Olgac, T., Obeid, R., & Biegel, D. E. (2023). Barriers to service and unmet need among autistic adolescents and young adults. *Autism: The International Journal of Research & Practice*, *27*(7), 1997–2010.** | | |
| --- | --- | --- |
| Study Characteristics | Population(s) of Interest | Rural, IDD |
|  | Study Population and Sample Size | 174 Caregivers |
|  | Medical Condition(s) | ASD |
|  | Study Purpose | To examine barriers experienced in accessing services for transition-age autistic youth and the degree to which those barriers are related to their unmet service needs. |
|  | Population(s) of interest are focus of the research | Yes |
|  | PCBEOs are a focus of the research | Yes |
|  | Data Collection Geography | Multiple sites in a single state |
|  | Data Source(s) | Primary survey data |
| Findings | PCBEO(s) Studied |  |
|  | Indirect impacts | Disruptions to daily life |
|  | Intangible burdens | Delaying or forgoing medical treatments due to costs, Changes in employment status and/or insurance coverage, Distress, stress, and anxiety directly caused by OOP costs, financial debt, or lost income (i.e., financial toxicity), General or composite measure of caregiver burden, General financial burden to due to patients needs or condition |
|  | Population-specific Justification Included | Yes |
|  | Justification(s) | Gaps in the literature; Policy factors |

| **Falk, D., Cubbin, C., Salsman, J. M., Winkfield, K. M., Foley, K. L., Noel, L., & Jones, B. (2023). Navigating financial barriers to Papanicolaou tests and mammograms for young adult women residing in rural and border areas of Texas. *Journal of Adolescent and Young Adult Oncology*, *12*(2), 159–167.** | | |
| --- | --- | --- |
| Study Characteristics | Population(s) of Interest | Rural |
|  | Study Population and Sample Size | 1,181 Patients |
|  | Medical Condition(s) | Medical treatments (Papanicolaou Tests and Mammograms) |
|  | Study Purpose | To (1) describe the sample of YA women participating in the program; (2) examine the prevalence of PN participation, Pap screening, and receipt of a mammogram; and (3) analyze financial barriers and other factors associated with receiving PN, Pap tests, and/or mammograms in this sample of rural and border Texan YA women. |
|  | Population(s) of interest are focus of the research | Yes |
|  | PCBEOs are a focus of the research | Yes |
|  | Data Collection Geography | Single institution/site |
|  | Data Source(s) | Primary survey data |
| Findings | PCBEO(s) Studied |  |
|  | Direct medical costs | Diagnosis/treatment/management costs (beyond cost sharing) |
|  | Population-specific Justification Included | No |

| **Dimachkie Nunnally, A., Factor, R. S., Sturm, A., Valluripalli Soorya, L., Wainer, A., Taylor, S., Ponzini, M., Abbeduto, L., & Gulsrud, A. C. (2023). Examining indicators of psychosocial risk and resilience in parents of autistic children. *Frontiers in Behavioral Neuroscience*, *17*.** | | |
| --- | --- | --- |
| Study Characteristics | Population(s) of Interest | IDD |
|  | Study Population and Sample Size | 106,577 Caregivers |
|  | Medical Condition(s) | ASD |
|  | Study Purpose | To examine the impact of child characteristics (restricted and repetitive behaviors, adaptive functioning and behavioral concerns) on parent mental health outcomes (caregiver strain, anxiety, depression and wellbeing). |
|  | Population(s) of interest are focus of the research | Yes |
|  | PCBEOs are a focus of the research | Yes |
|  | Data Collection Geography | National |
|  | Data Source(s) | Primary survey data |
| Findings | PCBEO(s) Studied |  |
|  | Intangible burdens | General or composite measure of caregiver burden |
|  | Population-specific Justification Included | Yes |
|  | Justification(s) | Gaps in the literature |

| **Balser, S., DiPierro, G., Chaikin, C. D., Obeid, R., Berg, K. A., & Ishler, K. J. (2023). “That he won’t experience life like he should”: Exploring worries of family caregivers of transition-age autistic youth. *Research in Autism Spectrum Disorders*, *109*, 14.** | | |
| --- | --- | --- |
| Study Characteristics | Population(s) of Interest | IDD |
|  | Study Population and Sample Size | 174 Caregivers |
|  | Medical Condition(s) | ASD |
|  | Study Purpose | To examine predictors of caregiver worry including caregiver characteristics, youth characteristics, and service characteristics. |
|  | Population(s) of interest are focus of the research | Yes |
|  | PCBEOs are a focus of the research | No |
|  | Data Collection Geography | Multiple sites in a single state |
|  | Data Source(s) | Primary survey data |
| Findings | PCBEO(s) Studied |  |
|  | Intangible burdens | Reduced spending on basic goods, groceries, and leisure activities |
|  | Population-specific Justification Included | Yes |
|  | Justification(s) | Predisposing factor(s): Age, SES; Enabling factor(s): Income |

| **Bachrach, R. L., & Quinn, D. A. (2023). The role of gender and veteran status in healthcare access among a national sample of U.S. adults with unhealthy alcohol use. *Substance Use & Misuse*, *58*(4), 491–499.** | | |
| --- | --- | --- |
| Study Characteristics | Population(s) of Interest | Veteran |
|  | Study Population and Sample Size | 58,816 Patients (6,902 Veteran) |
|  | Medical Condition(s) | SUD or OUD |
|  | Study Purpose | To examine differences in routine healthcare access, stratified by Veteran status and gender, among a national sample of adults endorsing unhealthy drinking. |
|  | Population(s) of interest are focus of the research | Yes |
|  | PCBEOs are a focus of the research | No |
|  | Data Collection Geography | National |
|  | Data Source(s) | Secondary survey data |
| Findings | PCBEO(s) Studied |  |
|  | Intangible burdens | Delaying or forgoing medical treatments due to costs |
|  | Population-specific Justification Included | Yes |
|  | Justification(s) | Predisposing factor(s): Gender/sex; Need factor(s): Perceived general health status |

| **Agbafe, V. C., Metzger, N., Garlick, B. R., Caverly, T., Saini, S., Kerr, E., Matloub, S., & Kullgren, J. T. (2023). Achieving greater value for veterans through full cost transparency in primary care. *Healthcare (Amsterdam)*, *11*(2), 100687.** | | |
| --- | --- | --- |
| Study Characteristics | Population(s) of Interest | Veteran |
|  | Study Population and Sample Size | 65 Patients |
|  | Medical Condition(s) | Medical condition not specified |
|  | Study Purpose | To 1) test the feasibility, acceptability, and preliminary effectiveness of Veterans receiving personalized information about their expected costs so they can obtain the most value from their primary care encounters and 2) create a scalable model to help patients optimize the value of their primary care encounters via transparent estimates of the healthcare-associated costs they could expect for different possible visit modalities. |
|  | Population(s) of interest are focus of the research | Yes |
|  | PCBEOs are a focus of the research | No |
|  | Data Collection Geography | Single institution/site |
|  | Data Source(s) | Secondary survey data |
| Findings | PCBEO(s) Studied |  |
|  | Direct medical costs | Copayments, coinsurance, deductibles, premiums |
|  | Direct non-medical costs | Transportation, meals, hoteling |
|  | Indirect impacts | Time to attend medical appointments, Time spent traveling to or between medical appointments |
|  | Population-specific Justification Included | No |

| **Williams, C. P., Davidoff, A., Halpern, M. T., Mollica, M., Castro, K., Allaire, B., & de Moor, J. S. (2022). Cost-Related medication nonadherence and patient cost responsibility for rural and urban cancer survivors. *JCO Oncology Practice*, *18*(8), e1234–e1246.** | | |
| --- | --- | --- |
| Study Characteristics | Population(s) of Interest | Rural |
|  | Study Population and Sample Size | 6,591 Patients (1,195 Rural) |
|  | Medical Condition(s) | Cancer |
|  | Study Purpose | To understand the relationship between OOP spending and cost-related medication nonadherence among older rural- and urban-dwelling cancer survivors. |
|  | Population(s) of interest are focus of the research | Yes |
|  | PCBEOs are a focus of the research | Yes |
|  | Data Collection Geography | National |
|  | Data Source(s) | Secondary survey data |
| Findings | PCBEO(s) Studied |  |
|  | Direct medical costs | Copayments, coinsurance, deductibles, premiums, Diagnosis/treatment/management costs (beyond cost sharing), Medication costs |
|  | Intangible burdens | Delaying or forgoing medical treatments due to costs |
|  | Population-specific Justification Included | Yes |
|  | Justification(s) | Enabling factor(s): Rurality, Distance to the nearest medical institution |

| **Spalding, W., Farahbakhshian, S., Maculaitis, M. C., Peck, E. Y., & Goren, A. (2022). The association of oral stimulant medication adherence with work productivity among adults with ADHD. *Journal of Attention Disorders*, *26*(6), 831–842.** | | |
| --- | --- | --- |
| Study Characteristics | Population(s) of Interest | IDD |
|  | Study Population and Sample Size | 602 Patients |
|  | Medical Condition(s) | ADHD |
|  | Study Purpose | To examine associations between oral psychostimulant pharmacotherapy adherence, work productivity, and related indirect costs among US adults with ADHD. |
|  | Population(s) of interest are focus of the research | Yes |
|  | PCBEOs are a focus of the research | Yes |
|  | Data Collection Geography | Multiple sites across multiple states |
|  | Data Source(s) | Primary survey data |
| Findings | PCBEO(s) Studied |  |
|  | Indirect impacts | Unpaid time off work, early retirement, Absenteeism and presenteeism |
|  | Population-specific Justification Included | Yes |
|  | Justification(s) | Gaps in the literature |

| **Shah, C. H., & Onukwugha, E. (2022). Direct medical and indirect absenteeism costs among working adult ADHD patients in the United States. *Expert Review of Pharmacoeconomics & Outcomes Research*, *22*(6), 1013–1020.** | | |
| --- | --- | --- |
| Study Characteristics | Population(s) of Interest | IDD |
|  | Study Population and Sample Size | 32,222 Patients |
|  | Medical Condition(s) | ADHD |
|  | Study Purpose | To investigate the direct medical and indirect (i.e. absenteeism) costs among working adults diagnosed with Attention-Deficit/Hyperactivity Disorder (ADHD) in the United States. |
|  | Population(s) of interest are focus of the research | Yes |
|  | PCBEOs are a focus of the research | Yes |
|  | Data Collection Geography | National |
|  | Data Source(s) | Secondary survey data |
| Findings | PCBEO(s) Studied |  |
|  | Direct medical costs | Medication costs |
|  | Indirect impacts | Unpaid time off work, early retirement |
|  | Population-specific Justification Included | Yes |
|  | Justification(s) | Enabling factor(s): High healthcare expenditures among individuals with ADHD; Gaps in the literature |

| **McGrath, L., Taunton, M., Levy, S., Kovacs, A. H., Broberg, C., & Khan, A. (2022). Barriers to care in urban and rural dwelling adults with congenital heart disease. *Cardiology in the Young*, *32*(4), 612–617.** | | |
| --- | --- | --- |
| Study Characteristics | Population(s) of Interest | Rural |
|  | Study Population and Sample Size | 100 Patients (18 Rural) |
|  | Medical Condition(s) | Cardiovascular disease |
|  | Study Purpose | To investigate the challenges faced by adults with CHD in accessing outpatient cardiac care, with a specific focus on understanding differences between urban and rural-dwelling patients. |
|  | Population(s) of interest are focus of the research | Yes |
|  | PCBEOs are a focus of the research | Yes |
|  | Data Collection Geography | Single institution/site |
|  | Data Source(s) | Primary survey data |
| Findings | PCBEO(s) Studied |  |
|  | Direct medical costs | Copayments, coinsurance, deductibles, premiums, Medication costs |
|  | Indirect impacts | Time spent traveling to or between medical appointments |
|  | Population-specific Justification Included | Yes |
|  | Justification(s) | Enabling factor(s): Income, Rurality; Policy factors |

| **Marsack-Topolewski, C. N., & Wang, F. (2022). Dimensions of caregiver burden between compound and noncompound caregivers of adults with autism. *Journal of Gerontological Social Work*, *65*(4), 402–420.** | | |
| --- | --- | --- |
| Study Characteristics | Population(s) of Interest | IDD |
|  | Study Population and Sample Size | 320 Caregivers |
|  | Medical Condition(s) | ASD |
|  | Study Purpose | To compare differences between compound 1, compound 2, and noncompound caregivers on specific dimensions of caregiver burden associated with adult children with ASD. |
|  | Population(s) of interest are focus of the research | Yes |
|  | PCBEOs are a focus of the research | Yes |
|  | Data Collection Geography | National |
|  | Data Source(s) | Primary survey data |
| Findings | PCBEO(s) Studied |  |
|  | Indirect impacts | Time spent providing unpaid caregiving |
|  | Intangible burdens | General or composite measure of caregiver burden, General financial burden, emotional burden |
|  | Population-specific Justification Included | Yes |
|  | Justification(s) | Need factor(s): Disability status; Gaps in the literature |

| **Lin, C., Dracup, K., Pelter, M. M., Biddle, M. J., & Moser, D. K. (2022). Association of psychological distress with reasons for delay in seeking medical care in rural patients with worsening heart failure symptoms. *Journal of Rural Health*, *38*(4), 713–720.** | | |
| --- | --- | --- |
| Study Characteristics | Population(s) of Interest | Rural |
|  | Study Population and Sample Size | 611 Patients |
|  | Medical Condition(s) | Cardiovascular disease, HF |
|  | Study Purpose | To (1) describe rural patients’ reasons for delay in seeking care for HF, and (2) to determine whether depressive symptoms or anxiety was associated with patients’ reasons for delay in seeking medical care for worsening symptoms. |
|  | Population(s) of interest are focus of the research | Yes |
|  | PCBEOs are a focus of the research | No |
|  | Data Collection Geography | Multiple sites across multiple states |
|  | Data Source(s) | Secondary survey data |
| Findings | PCBEO(s) Studied |  |
|  | Intangible burdens | Delaying or forgoing medical treatments due to costs |
|  | Population-specific Justification Included | Yes |
|  | Justification(s) | Gaps in the literature; Policy factors |

| **DiFazio, R. L., Vessey, J. A., Miller, P. E., Snyder, B. D., & Shore, B. J. (2022). Health-related quality of life and caregiver burden after hip reconstruction and spinal fusion in children with spastic cerebral palsy. *Developmental Medicine & Child Neurology*, *64*(1), 80–87.** | | |
| --- | --- | --- |
| Study Characteristics | Population(s) of Interest | IDD |
|  | Study Population and Sample Size | 69 Caregivers |
|  | Medical Condition(s) | Hip instability or scoliosis, Cerebral Palsy |
|  | Study Purpose | To evaluate the effect of hip reconstruction or spinal fusion on health-related quality of life (HRQoL) in non-ambulatory children with spastic cerebral palsy (CP) and caregiver burden. |
|  | Population(s) of interest are focus of the research | Yes |
|  | PCBEOs are a focus of the research | Yes |
|  | Data Collection Geography | Single institution/site |
|  | Data Source(s) | Primary survey data |
| Findings | PCBEO(s) Studied |  |
|  | Direct non-medical costs | General financial burden of caregiving |
|  | Indirect impacts | Time spent providing unpaid caregiving |
|  | Intangible burdens | General or composite measure of caregiver burden |
|  | Population-specific Justification Included | No |

| **Crasta, D., Spears, A. P., Sullivan, S. R., Britton, P. C., & Goodman, M. (2022). Better off with you: Exploring congruity between caregivers’ and veterans’ experience of efforts to cope with suicide. *Military Psychology*, *34*(3), 326–334.** | | |
| --- | --- | --- |
| Study Characteristics | Population(s) of Interest | Veteran |
|  | Study Population and Sample Size | 43 Patients, 43 Caregivers |
|  | Medical Condition(s) | Suicidal ideation |
|  | Study Purpose | To examine the association between interpersonal perceptions related to suicide and social coping efforts. |
|  | Population(s) of interest are focus of the research | Yes |
|  | PCBEOs are a focus of the research | No |
|  | Data Collection Geography | Single institution/site |
|  | Data Source(s) | Primary survey data |
| Findings | PCBEO(s) Studied |  |
|  | Intangible burdens | General or composite measure of caregiver burden |
|  | Population-specific Justification Included | Yes |
|  | Justification(s) | Gaps in the literature; Policy factors |

| **Corkum, J., Zhu, V. C., Agbafe, V., Sun, S. X., Chu, C. R., Colen, J. S., Greenup, R., & Offodile, A. C. (2022). Area Deprivation Index and rurality in relation to financial toxicity among breast cancer surgical patients: Retrospective cross-sectional study of geospatial differences in risk profiles. *Journal of the American College of Surgeons*, *234*(5), 816–826.** | | |
| --- | --- | --- |
| Study Characteristics | Population(s) of Interest | Rural |
|  | Study Population and Sample Size | 568 Patients (64 Rural) |
|  | Medical Condition(s) | Breast cancer |
|  | Study Purpose | To examine the relationship between geospatial location, represented by rurality and Area Deprivation Index (ADI), and risk of FT |
|  | Population(s) of interest are focus of the research | Yes |
|  | PCBEOs are a focus of the research | Yes |
|  | Data Collection Geography | Single institution/site |
|  | Data Source(s) | Primary survey data |
| Findings | PCBEO(s) Studied |  |
|  | Intangible burdens | Distress, stress, and anxiety directly caused by OOP costs, financial debt, or lost income (i.e., financial toxicity) |
|  | Population-specific Justification Included | Yes |
|  | Justification(s) | Gaps in the literature |

| **Chen, Z., Roy, K., Khushalani, J. S., & Puddy, R. W. (2022). Trend in rural-urban disparities in access to outpatient mental health services among US adults aged 18-64 with employer-sponsored insurance: 2005-2018. *Journal of Rural Health*, *38*(4), 788–794.** | | |
| --- | --- | --- |
| Study Characteristics | Population(s) of Interest | Rural |
|  | Study Population and Sample Size | Unknown |
|  | Medical Condition(s) | Depression, SUD or OUD, Anxiety disorder |
|  | Study Purpose | To estimate the rural-urban differences in outpatient service utilization and expenditures for depression, anxiety disorder, and substance use disorder, and the evolving mental health provider mix for privately insured US adults aged 18–64 during 2005–2018. |
|  | Population(s) of interest are focus of the research | Yes |
|  | PCBEOs are a focus of the research | No |
|  | Data Collection Geography | National |
|  | Data Source(s) | Medical claims |
| Findings | PCBEO(s) Studied |  |
|  | Direct medical costs | general OOP costs |
|  | Population-specific Justification Included | Yes |
|  | Justification(s) | Enabling factor(s): Income, Rurality, Distance to the nearest medical institution, unemployment; Need factor(s): Chronic illnesses; Policy factors |

| **Calixte, R., Helzner, E. P., Islam, S., Camacho-Rivera, M., & Pati, S. (2022). Unmet medical needs and food insecurity in children with neurodevelopmental disorders: Findings from the 2019 National Health Interview Survey (NHIS). *Children*, *9*(12), 1798.** | | |
| --- | --- | --- |
| Study Characteristics | Population(s) of Interest | IDD |
|  | Study Population and Sample Size | 1,287 Patients |
|  | Medical Condition(s) | Neurodevelopmental disorders |
|  | Study Purpose | To identify sociodemographic characteristics of children with neurodevelopmental disorders (NDD) that are predictive of unmet medical needs and food insecurity. |
|  | Population(s) of interest are focus of the research | Yes |
|  | PCBEOs are a focus of the research | Yes |
|  | Data Collection Geography | National |
|  | Data Source(s) | Secondary survey data |
| Findings | PCBEO(s) Studied |  |
|  | Intangible burdens | Delaying or forgoing medical treatments due to costs |
|  | Population-specific Justification Included | No |

| **Burch, A. E. (2022). Factors responsible for healthcare avoidance among rural adults in the Eastern region of North Carolina. *Journal of Community Health*, *47*(5), 737–744.** | | |
| --- | --- | --- |
| Study Characteristics | Population(s) of Interest | Rural |
|  | Study Population and Sample Size | 946 Patients |
|  | Medical Condition(s) | Medical treatments |
|  | Study Purpose | To extend prior work by examining the antecedents to these barriers and determining the extent to which they con-tribute to healthcare avoidance in a rural population. |
|  | Population(s) of interest are focus of the research | Yes |
|  | PCBEOs are a focus of the research | Yes |
|  | Data Collection Geography | Multiple sites in a single state |
|  | Data Source(s) | Primary survey data |
| Findings | PCBEO(s) Studied |  |
|  | Intangible burdens | Delaying or forgoing medical treatments due to costs |
|  | Population-specific Justification Included | No |

| **Xu, H., Kadambi, S., Mohile, S. G., Yang, S., Kehoe, L. A., Wells, M., Culakova, E., Kamen, C., Obrecht, S., Mohamed, M., Gilmore, N. J., Magnuson, A., Grossman, V. A., Hopkins, J. O., Geer, J., Berenberg, J., Mustian, K., Cupertino, A., Mohile, N., & Loh, K. P. (2021). Caregiving burden of informal caregivers of older adults with advanced cancer: The effects of rurality and education. *Journal of Geriatric Oncology*, *12*(7), 1015–1021.** | | |
| --- | --- | --- |
| Study Characteristics | Population(s) of Interest | Rural |
|  | Study Population and Sample Size | 414 Caregivers (64 Rural) |
|  | Medical Condition(s) | Advanced solid tumor or lymphoma |
|  | Study Purpose | To 1) evaluate factors (including patient GA impairments) associated with caregiver mastery and caregiving burden among caregivers of older adults with advanced cancer; 2) examine rural-urban disparities in caregiver mastery and burden; and 3) explore whether rural-urban disparities in caregiver mastery and burden differed by caregiver education. |
|  | Population(s) of interest are focus of the research | Yes |
|  | PCBEOs are a focus of the research | Yes |
|  | Data Collection Geography | Multiple sites across multiple states |
|  | Data Source(s) | Primary survey data |
| Findings | PCBEO(s) Studied |  |
|  | Indirect impacts | Disrupted schedule |
|  | Intangible burdens | Distress, stress, and anxiety directly caused by OOP costs, financial debt, or lost income (i.e., financial toxicity), General or composite measure of caregiver burden |
|  | Population-specific Justification Included | Yes |
|  | Justification(s) | Gaps in the literature; Policy factors |

| **Solomon, D. L., Dirlikov, B., Shem, K. L., & Elliott, C. S. (2021). The time burden of specialty clinic visits in persons with neurologic disease: A case for universal telemedicine coverage. *Frontiers in Neurology*, *12*, 559024.** | | |
| --- | --- | --- |
| Study Characteristics | Population(s) of Interest | Rural, IDD |
|  | Study Population and Sample Size | 208 Patients |
|  | Medical Condition(s) | Chronic neurologic disorders |
|  | Study Purpose | To examine the burden of time associated with clinical visits for those with chronic neurologic disorders and their family/caregivers |
|  | Population(s) of interest are focus of the research | Yes |
|  | PCBEOs are a focus of the research | Yes |
|  | Data Collection Geography | Single institution/site |
|  | Data Source(s) | Primary survey data |
| Findings | PCBEO(s) Studied |  |
|  | Direct non-medical costs | Transportation, meals, hoteling |
|  | Indirect impacts | Time spent traveling to or between medical appointments |
|  | Population-specific Justification Included | Yes |
|  | Justification(s) | Need factor(s): Chronic illnesses, Disability status; Policy factors |

| **Singal, A. G., Tiro, J. A., Murphy, C. C., Blackwell, J. M., Kramer, J. R., Khan, A., Liu, Y., Zhang, S., Phillips, J. L., & Hernaez, R. (2021). Patient-reported barriers are associated with receipt of hepatocellular carcinoma surveillance in a multicenter cohort of patients with cirrhosis. *Clinical Gastroenterology and Hepatology*, *19*(5), 987.** | | |
| --- | --- | --- |
| Study Characteristics | Population(s) of Interest | Veteran |
|  | Study Population and Sample Size | 2,871 Patients |
|  | Medical Condition(s) | Cirrhosis |
|  | Study Purpose | To characterize patient knowledge, attitudes, and barriers of HCC surveillance and their association with surveillance receipt in a racially and socioeconomically diverse cohort of patients with cirrhosis followed in three different health systems. |
|  | Population(s) of interest are focus of the research | Yes |
|  | PCBEOs are a focus of the research | No |
|  | Data Collection Geography | Multiple sites across multiple states |
|  | Data Source(s) | Primary survey data, Medical records/EHRs |
| Findings | PCBEO(s) Studied |  |
|  | Direct medical costs | Copayments, coinsurance, deductibles, premiums, Medical financial debt |
|  | Intangible burdens | Delaying or forgoing medical treatments due to costs, Distress, stress, and anxiety directly caused by OOP costs, financial debt, or lost income (i.e., financial toxicity) |
|  | Population-specific Justification Included | No |

| **Pattison, A., Torres, E., Wieters, L., & Waldschmidt, J. G. (2021). A comparison of caregiver burden between long-term care and developmental disability family caregivers. *Journal of Research in Nursing*, *26*(8), 781–793.** | | |
| --- | --- | --- |
| Study Characteristics | Population(s) of Interest | IDD |
|  | Study Population and Sample Size | 72 Caregivers |
|  | Medical Condition(s) | IDD |
|  | Study Purpose | To examine the burden that family caregivers experience so they can seek out additional training and services to maintain their own well-being. The researchers examined caregiver burden from a perspective of developmentally disabled and long-term care. |
|  | Population(s) of interest are focus of the research | Yes |
|  | PCBEOs are a focus of the research | Yes |
|  | Data Collection Geography | Not stated/unclear |
|  | Data Source(s) | Primary survey data |
| Findings | PCBEO(s) Studied |  |
|  | Intangible burdens | General or composite measure of caregiver burden |
|  | Population-specific Justification Included | Yes |
|  | Justification(s) | Gaps in the literature |

| **Marsack-Topolewski, C. N., Samuel, P. S., & Tarraf, W. (2021). Empirical evaluation of the association between daily living skills of adults with autism and parental caregiver burden. *PLoS One*, 16(1), e0244844.** | | |
| --- | --- | --- |
| Study Characteristics | Population(s) of Interest | IDD |
|  | Study Population and Sample Size | 320 Caregivers |
|  | Medical Condition(s) | ASD |
|  | Study Purpose | To examine the relationship between the ability of adults with ASD to perform ADLs with parental perceptions of caregiver burden. |
|  | Population(s) of interest are focus of the research | Yes |
|  | PCBEOs are a focus of the research | Yes |
|  | Data Collection Geography | National |
|  | Data Source(s) | Primary survey data |
| Findings | PCBEO(s) Studied |  |
|  | Direct non-medical costs | Economic costs of access to age-appropriate health care, educational, and career opportunities |
|  | Indirect impacts | Time spent providing unpaid caregiving, Unpaid time off work, early retirement |
|  | Intangible burdens | Hindered career or education advancement, General or composite measure of caregiver burden |
|  | Population-specific Justification Included | Yes |
|  | Justification(s) | Need factor(s): Chronic illnesses, Disability status; Gaps in the literature; Policy factors |

| **Marsack-Topolewski, C. N. (2021). Mediating effects of social support on caregiver burden and quality of life for compound and noncompound caregivers. *Families in Society: The Journal of Contemporary Social Services*, *102*(2), 240–252.** | | |
| --- | --- | --- |
| Study Characteristics | Population(s) of Interest | IDD |
|  | Study Population and Sample Size | 320 Caregivers |
|  | Medical Condition(s) | ASD |
|  | Study Purpose | To explore the mediating effect of informal social support on the relationship between caregiver burden and quality of life among compound and noncompound caregivers |
|  | Population(s) of interest are focus of the research | Yes |
|  | PCBEOs are a focus of the research | No |
|  | Data Collection Geography | National |
|  | Data Source(s) | Primary survey data |
| Findings | PCBEO(s) Studied |  |
|  | Intangible burdens | General or composite measure of caregiver burden |
|  | Population-specific Justification Included | Yes |
|  | Justification(s) | Enabling factor(s): Partner and social support or total help received from relatives; Need factor(s): Disability status; Gaps in the literature |

| **Marsack-Topolewski, C. N. (2021). Relationship between caregiver burden and basic and instrumental activities of daily living among compound and noncompound caregivers. *Journal of Family Social Work*, *24*(4), 299–319.** | | |
| --- | --- | --- |
| Study Characteristics | Population(s) of Interest | IDD |
|  | Study Population and Sample Size | 320 Caregivers |
|  | Medical Condition(s) | ASD |
|  | Study Purpose | To investigate differences in relationships between caregiver burden and basic ADLs (BADLs) and instrumental ADLs (IADLs) between compound and noncompound caregivers. |
|  | Population(s) of interest are focus of the research | Yes |
|  | PCBEOs are a focus of the research | Yes |
|  | Data Collection Geography | National |
|  | Data Source(s) | Primary survey data |
| Findings | PCBEO(s) Studied |  |
|  | Indirect impacts | Time spent providing unpaid caregiving |
|  | Intangible burdens | General or composite measure of caregiver burden |
|  | Population-specific Justification Included | Yes |
|  | Justification(s) | Need factor(s): Disability status; Gaps in the literature |

| **Li, H., Parish, S. L., Magaña, S., & Morales, M. A. (2021). Racial and ethnic disparities in perceived barriers to health care among U.S. adults with intellectual and developmental disabilities. *Intellectual and Developmental Disabilities*, *59*(1), 84–94.** | | |
| --- | --- | --- |
| Study Characteristics | Population(s) of Interest | IDD |
|  | Study Population and Sample Size | 1,131 Patients |
|  | Medical Condition(s) | IDD |
|  | Study Purpose | To use nationally representative data to examine barriers to health care among community-dwelling U.S. adults with IDD across three racial and ethnic groups: Non-Latinx White, Non-Latinx Black, and Latinx adults |
|  | Population(s) of interest are focus of the research | Yes |
|  | PCBEOs are a focus of the research | Yes |
|  | Data Collection Geography | National |
|  | Data Source(s) | Secondary survey data |
| Findings | PCBEO(s) Studied |  |
|  | Intangible burdens | Delaying or forgoing medical treatments due to costs |
|  | Population-specific Justification Included | Yes |
|  | Justification(s) | Predisposing factor(s): Medical distrust; Need factor(s): Disability status |

| **Koenig, J. L., Sandhu, N., Sborov, K., Sabolch, A., Usoz, M., Li, G., Gephart, M. H., Chang, S., Hiniker, S., Soltys, S. G., & Pollom, E. L. (2021). Financial toxicity in patients with brain and spine metastases. *World Neurosurgery*, *151*, e630–e651.** | | |
| --- | --- | --- |
| Study Characteristics | Population(s) of Interest | Veteran |
|  | Study Population and Sample Size | 93 Patients (2 Veteran) |
|  | Medical Condition(s) | Brain cancer, Spine metastases |
|  | Study Purpose | To characterize the extent of financial toxicity among this population and identify factors associated with financial toxicity |
|  | Population(s) of interest are focus of the research | No |
|  | PCBEOs are a focus of the research | Yes |
|  | Data Collection Geography | Single institution/site |
|  | Data Source(s) | Primary survey data, Medical records/EHRs |
| Findings | PCBEO(s) Studied |  |
|  | Intangible burdens | Distress, stress, and anxiety directly caused by OOP costs, financial debt, or lost income (i.e., financial toxicity) |
|  | Population-specific Justification Included | No |

| **Jiang, C. Y., Strohbehn, G. W., Dedinsky, R. M., Raupp, S. M., Pannecouk, B. M., Yentz, S. E., & Ramnath, N. (2021). Teleoncology for veterans: High patient satisfaction coupled with positive financial and environmental impacts. *JCO Oncology Practice*, *17*(9), 580.** | | |
| --- | --- | --- |
| Study Characteristics | Population(s) of Interest | Rural, Veteran |
|  | Study Population and Sample Size | 560 Patients |
|  | Medical Condition(s) | Medical condition not specified |
|  | Study Purpose | To generate estimates of both private and social, financial, and environmental impacts of teleoncology to inform relevant policy tradeoffs in the future. |
|  | Population(s) of interest are focus of the research | Yes |
|  | PCBEOs are a focus of the research | No |
|  | Data Collection Geography | National |
|  | Data Source(s) | Medical records/EHRs |
| Findings | PCBEO(s) Studied |  |
|  | Indirect impacts | Time spent traveling to or between medical appointments |
|  | Population-specific Justification Included | No |

| **Jabbarinejad, R., Cohen-Zimerman, S., Wagner, A. K., & Grafman, J. (2021). Determinants of caregiver burden in male patients with epilepsy following penetrating traumatic brain injury. *Epilepsy & Behavior*, *116*, 107768.** | | |
| --- | --- | --- |
| Study Characteristics | Population(s) of Interest | Veteran |
|  | Study Population and Sample Size | 331 Caregivers |
|  | Medical Condition(s) | Epilepsy, TBI |
|  | Study Purpose | To determine burden of caring for patients with post-traumatic epilepsy (PTE) following penetrating traumatic brain injury (TBI) and identify factors predicting higher burden. |
|  | Population(s) of interest are focus of the research | Yes |
|  | PCBEOs are a focus of the research | Yes |
|  | Data Collection Geography | Not stated/unclear |
|  | Data Source(s) | Primary survey data |
| Findings | PCBEO(s) Studied |  |
|  | Intangible burdens | General or composite measure of caregiver burden |
|  | Population-specific Justification Included | No |

| **Fosse, C., Luo, H., Laniado, N., Okunseri, C., & Badner, V. (2021). Dental access and expenditures for adults with intellectual and other disabilities. *Journal of Public Health Dentistry*, *81*(4), 299–307.** | | |
| --- | --- | --- |
| Study Characteristics | Population(s) of Interest | IDD |
|  | Study Population and Sample Size | 17,946 Patients (736 IDD) |
|  | Medical Condition(s) | IDD |
|  | Study Purpose | To examine dental access, utilization, expenditures, and sources of payment between adults with intellectual disabilities (ID), other types of disabilities, and without disabilities. |
|  | Population(s) of interest are focus of the research | Yes |
|  | PCBEOs are a focus of the research | No |
|  | Data Collection Geography | National |
|  | Data Source(s) | Secondary survey data |
| Findings | PCBEO(s) Studied |  |
|  | Direct medical costs | Copayments, coinsurance, deductibles, premiums, Diagnosis/treatment/management costs (beyond cost sharing) |
|  | Population-specific Justification Included | Yes |
|  | Justification(s) | Enabling factor(s): Health care access; Need factor(s): Chronic illnesses, Disability status; Policy factors |

| **Shepherd-Banigan, M., Sherman, S. R., Lindquist, J. H., Miller, K. E. M., Tucker, M., Smith, V. A., & Van Houtven, C. H. (2020). Family caregivers of veterans experience high levels of burden, distress, and financial strain. *Journal of the American Geriatrics Society*, *68*(11), 2675–2683.** | | |
| --- | --- | --- |
| Study Characteristics | Population(s) of Interest | Veteran |
|  | Study Population and Sample Size | 1,509 Caregivers |
|  | Medical Condition(s) | Medical condition not specified |
|  | Study Purpose | To describe the caregiving experiences and physical and emotional needs of family members and friends who provide care to veterans with mental, physical, and cognitive comorbidities. |
|  | Population(s) of interest are focus of the research | Yes |
|  | PCBEOs are a focus of the research | Yes |
|  | Data Collection Geography | National |
|  | Data Source(s) | Primary survey data |
| Findings | PCBEO(s) Studied |  |
|  | Indirect impacts | Time spent providing unpaid caregiving |
|  | Intangible burdens | Family/social life impacts, Distress, stress, and anxiety directly caused by OOP costs, financial debt, or lost income (i.e., financial toxicity), General or composite measure of caregiver burden |
|  | Population-specific Justification Included | Yes |
|  | Justification(s) | Need factor(s): Chronic illnesses; Gaps in the literature; Policy factors |

| **Schwartz, A. J., & Jenkins, C. L. (2020). Barriers and facilitators to cancer treatment adherence for older rural African Americans: Understanding the experience from the view of patients and their caregivers. *Journal of Family Social Work*, *23*(1), 20–34.** | | |
| --- | --- | --- |
| Study Characteristics | Population(s) of Interest | Rural |
|  | Study Population and Sample Size | 9 Patients, 9 Caregivers |
|  | Medical Condition(s) | Cancer |
|  | Study Purpose | To understand the barriers that a particularly vulnerable sub-population of older adults experience in adhering to cancer treatments in rural eastern North Carolina. |
|  | Population(s) of interest are focus of the research | Yes |
|  | PCBEOs are a focus of the research | Yes |
|  | Data Collection Geography | Single institution/site |
|  | Data Source(s) | Interviews |
| Findings | PCBEO(s) Studied |  |
|  | Direct medical costs | Medication costs, Personal medical equipment or consumables costs |
|  | Population-specific Justification Included | Yes |
|  | Justification(s) | Gaps in the literature |

| **Rosenberg, M. W., & Eckstrom, E. (2020). Geographic differences in the unpaid caregiver experience from the National Study of Caregiving (NSOC). *Rural Remote Health*, 20(4), 6062.** | | |
| --- | --- | --- |
| Study Characteristics | Population(s) of Interest | Rural |
|  | Study Population and Sample Size | 2,278 Caregivers (429 Rural) |
|  | Medical Condition(s) | Dementia, Old age |
|  | Study Purpose | To identify unpaid caregiver differences in demographics, competing work responsibilities, support, health, caregiver burden, and interaction with healthcare professionals in US metropolitan and non-metropolitan settings. |
|  | Population(s) of interest are focus of the research | Yes |
|  | PCBEOs are a focus of the research | No |
|  | Data Collection Geography | National |
|  | Data Source(s) | Secondary survey data |
| Findings | PCBEO(s) Studied |  |
|  | Direct non-medical costs | Child/dependent care costs |
|  | Indirect impacts | Time spent traveling to or between medical appointments, Unpaid time off work, early retirement |
|  | Intangible burdens | Reduced spending on basic goods, groceries, and leisure activities, General or composite measure of caregiver burden |
|  | Population-specific Justification Included | Yes |
|  | Justification(s) | Predisposing factor(s): Cultural differences; Gaps in the literature |

| **Owen, J. E., Jaworski, B. K., Kuhn, E., Hoffman, J. E., Schievelbein, L., Chang, A., Ramsey, K., & Rosen, C. (2020). Development of a mobile app for family members of veterans with PTSD: Identifying needs and modifiable factors associated with burden, depression, and anxiety. *Journal of Family Studies*, *26*(2), 286–307.** | | |
| --- | --- | --- |
| Study Characteristics | Population(s) of Interest | Veteran |
|  | Study Population and Sample Size | 212 Caregivers |
|  | Medical Condition(s) | PTSD |
|  | Study Purpose | To characterize caregiver burden in those seeking a mobile app for self-management of stress symptoms and to develop a model to guide mobile interventions for family members. |
|  | Population(s) of interest are focus of the research | No |
|  | PCBEOs are a focus of the research | Yes |
|  | Data Collection Geography | National |
|  | Data Source(s) | Primary survey data |
| Findings | PCBEO(s) Studied |  |
|  | Intangible burdens | General or composite measure of caregiver burden |
|  | Population-specific Justification Included | Yes |
|  | Justification(s) | Enabling factor(s): Medical insurance; Gaps in the literature |

| **Meehan, K. R., Meehan, J. M., Hill, J. M., Caldon, K. L., Root, L. D., Labrie, B., Brighton, S., Hayes, C. A., & Lowrey, C. H. (2020). Caregivers’ out-of-pocket expenses and time commitment following hematopoietic stem cell transplantation at a rural cancer center. *Biology of Blood and Marrow Transplantation*, *26*(9), E227–E231.** | | |
| --- | --- | --- |
| Study Characteristics | Population(s) of Interest | Rural |
|  | Study Population and Sample Size | 49 Caregivers |
|  | Medical Condition(s) | Cancer |
|  | Study Purpose | To assess the out-of pocket expenses and time commitment of caregivers of hematopoietic stem cell transplantation recipients within the first 4 weeks after discharge from the hospital from a National Cancer Institute (NCI)-designated comprehensive cancer center. |
|  | Population(s) of interest are focus of the research | Yes |
|  | PCBEOs are a focus of the research | Yes |
|  | Data Collection Geography | Multiple sites across multiple states |
|  | Data Source(s) | Primary survey data |
| Findings | PCBEO(s) Studied |  |
|  | Direct medical costs | Copayments, coinsurance, deductibles, premiums, Medication costs |
|  | Direct non-medical costs | Transportation, meals, hoteling |
|  | Indirect impacts | Time to attend medical appointments, Unpaid time off work, early retirement |
|  | Population-specific Justification Included | Yes |
|  | Justification(s) | Gaps in the literature |

| **Hallgren, E., Hastert, T. A., Carnahan, L. R., Eberth, J. M., Mama, S. K., Watson, K. S., & Molina, Y. (2020). Cancer-related debt and mental-health-related quality of life among rural cancer survivors: Do family/friend informal caregiver networks moderate the relationship? *Journal of Health and Social Behavior*, *61*(1), 113–130.** | | |
| --- | --- | --- |
| Study Characteristics | Population(s) of Interest | Rural |
|  | Study Population and Sample Size | 135 Patients (83 Rural) |
|  | Medical Condition(s) | Cancer |
|  | Study Purpose | To examine the moderating role of cancer survivors’ family/ friend informal caregiver networks on the relationship between cancer-related debt and mental health-related quality of life (MHQOL) among rural cancer survivors. |
|  | Population(s) of interest are focus of the research | Yes |
|  | PCBEOs are a focus of the research | No |
|  | Data Collection Geography | Multiple sites in a single state |
|  | Data Source(s) | Secondary survey data |
| Findings | PCBEO(s) Studied |  |
|  | Direct medical costs | Medical financial debt |
|  | Population-specific Justification Included | Yes |
|  | Justification(s) | Enabling factor(s): Rurality; Gaps in the literature |

| **Brickell, T. A., French, L. M., Lippa, S. M., Wright, M. M., & Lange, R. T. (2020). Caring for a service member or veteran following traumatic brain injury influences caregiver mental health. *Military Psychology*, *32*(4), 341–351.** | | |
| --- | --- | --- |
| Study Characteristics | Population(s) of Interest | Veteran |
|  | Study Population and Sample Size | 201 Caregivers |
|  | Medical Condition(s) | TBI |
|  | Study Purpose | To examine factors related to poor mental health in caregivers assisting service members and Veterans (SMV) following traumatic brain injury (TBI). |
|  | Population(s) of interest are focus of the research | Yes |
|  | PCBEOs are a focus of the research | Yes |
|  | Data Collection Geography | National |
|  | Data Source(s) | Primary survey data |
| Findings | PCBEO(s) Studied |  |
|  | Indirect impacts | Time spent providing unpaid caregiving |
|  | Intangible burdens | Hindered career or education advancement, Distress, stress, and anxiety directly caused by OOP costs, financial debt, or lost income (i.e., financial toxicity), General or composite measure of caregiver burden |
|  | Population-specific Justification Included | No |

| **Bowen, M. E., Gaynor, B., Phillips, L. J., Orsega-Smith, E., Lavery, A., Mitchell, A. B., & Basehore, H. K. (2020). Factors associated with health service use among older adults in a mobile veterans program. *Journal of Gerontological Nursing*, *46*(5), 15–22.** | | |
| --- | --- | --- |
| Study Characteristics | Population(s) of Interest | Veteran |
|  | Study Population and Sample Size | 54 Caregivers |
|  | Medical Condition(s) | Old age |
|  | Study Purpose | To examine how demographic characteristics (age), available resources (e.g., a caregiver, the Mobile Veterans Program [MVP]) and health needs (e.g., cognitive and physical functioning, depressive symptoms) affect hospitalization and institutionalization outcomes among older adults using the MVP. |
|  | Population(s) of interest are focus of the research | Yes |
|  | PCBEOs are a focus of the research | No |
|  | Data Collection Geography | Not stated/unclear |
|  | Data Source(s) | Primary survey data |
| Findings | PCBEO(s) Studied |  |
|  | Intangible burdens | General or composite measure of caregiver burden |
|  | Population-specific Justification Included | Yes |
|  | Justification(s) | Enabling factors; Need factor(s): Chronic illnesses, Disability status |

| **Powell, W., Jacobs, J. A., Noble, W., Bush, M. L., & Snell-Rood, C. (2019). Rural adult perspectives on impact of hearing loss and barriers to care. *Journal of Community Health*, *44*(4), 668–674.** | | |
| --- | --- | --- |
| Study Characteristics | Population(s) of Interest | Rural |
|  | Study Population and Sample Size | 40 Patients |
|  | Medical Condition(s) | Hearing loss |
|  | Study Purpose | To characterize the impact of hearing loss and the barriers for hearing healthcare in rural adults. |
|  | Population(s) of interest are focus of the research | Yes |
|  | PCBEOs are a focus of the research | No |
|  | Data Collection Geography | Multiple sites in a single state |
|  | Data Source(s) | Interviews |
| Findings | PCBEO(s) Studied |  |
|  | Direct medical costs | Diagnosis/treatment/management costs (beyond cost sharing), Personal medical equipment or consumables costs |
|  | Intangible burdens | Delaying or forgoing medical treatments due to costs |
|  | Population-specific Justification Included | Yes |
|  | Justification(s) | Enabling factor(s): Rurality, Distance to the nearest medical institution, Environmental exposure; Need factor(s): Environmental exposure; Gaps in the literature; Policy factors |

| **Patel, B. (2019). A mixed methods inquiry of caregivers of U.S. veterans with sustained “invisible” injuries from Iraq/Afghanistan. *Journal of Human Behavior in the Social Environment*, *29*(8), 979–996.** | | |
| --- | --- | --- |
| Study Characteristics | Population(s) of Interest | Veteran |
|  | Study Population and Sample Size | 172 Caregivers |
|  | Medical Condition(s) | Serious injury |
|  | Study Purpose | To explore how specific factors, including demographic characteristics, whether or not the caregiver has children in the home, assigned stipend/tier levels within the Caregiver Support Program, and veteran diagnoses, impact levels of caregiver burden. |
|  | Population(s) of interest are focus of the research | Yes |
|  | PCBEOs are a focus of the research | Yes |
|  | Data Collection Geography | Multiple sites across multiple states |
|  | Data Source(s) | Medical records/EHRs, Interviews |
| Findings | PCBEO(s) Studied |  |
|  | Intangible burdens | General or composite measure of caregiver burden |
|  | Population-specific Justification Included | Yes |
|  | Justification(s) | Predisposing factor(s): Age; Need factor(s): Chronic illnesses |

| **Marsack-Topolewski, C. N., & Church, H. L. (2019). Impact of caregiver burden on quality of life for parents of adult children with autism spectrum disorder. *American Journal on Intellectual and Developmental Disabilities*, 124(2), 145–156.** | | |
| --- | --- | --- |
| Study Characteristics | Population(s) of Interest | IDD |
|  | Study Population and Sample Size | 320 Caregivers |
|  | Medical Condition(s) | ASD |
|  | Study Purpose | To examine the impact of time, developmental, emotional, and financial burdens on the quality of life (QOL) for parents (aged 50+) of an adult child with autism spectrum disorder (ASD). |
|  | Population(s) of interest are focus of the research | Yes |
|  | PCBEOs are a focus of the research | No |
|  | Data Collection Geography | National |
|  | Data Source(s) | Primary survey data, Interviews |
| Findings | PCBEO(s) Studied |  |
|  | Direct non-medical costs | Child/dependent care costs |
|  | Indirect impacts | Time spent providing unpaid caregiving, Unpaid time off work, early retirement |
|  | Intangible burdens | Diminished household wealth, Fear and anxiety about future ability to afford medical treatments, Hindered career or education advancement, Quality-of-life impacts (not QALYs or DALYs) due to medical costs, Distress, stress, and anxiety directly caused by OOP costs, financial debt, or lost income (i.e., financial toxicity) |
|  | Population-specific Justification Included | Yes |
|  | Justification(s) | Need factor(s): Disability status; Gaps in the literature |

| **Marsack, C. N., & Hopp, F. P. (2019). Informal support, health, and burden among parents of adult children with autism. *Gerontologist*, *59*(6), 1112–1121.** | | |
| --- | --- | --- |
| Study Characteristics | Population(s) of Interest | IDD |
|  | Study Population and Sample Size | 320 Caregivers |
|  | Medical Condition(s) | ASD |
|  | Study Purpose | To understand if caregiver health moderates the relationship between informal social support and caregiver burden. |
|  | Population(s) of interest are focus of the research | Yes |
|  | PCBEOs are a focus of the research | Yes |
|  | Data Collection Geography | National |
|  | Data Source(s) | Primary survey data |
| Findings | PCBEO(s) Studied |  |
|  | Direct medical costs | General financial burden |
|  | Indirect impacts | Time spent providing unpaid caregiving |
|  | Intangible burdens | General or composite measure of caregiver burden |
|  | Population-specific Justification Included | Yes |
|  | Justification(s) | Enabling factor(s): Partner and social support or total help received from relatives; Need factor(s): Disability status |

| **Jacobs, J. C., Van Houtven, C. H., Tanielian, T., & Ramchand, R. (2019). Economic spillover effects of intensive unpaid caregiving. *Pharmacoeconomics*, *37*(4), 553–562.** | | |
| --- | --- | --- |
| Study Characteristics | Population(s) of Interest | Veteran |
|  | Study Population and Sample Size | 3,876 Caregivers |
|  | Medical Condition(s) | Medical condition not specified |
|  | Study Purpose | To explore how intensive caregiving is associated with economic outcomes for caregivers aged 18 years and older. |
|  | Population(s) of interest are focus of the research | No |
|  | PCBEOs are a focus of the research | Yes |
|  | Data Collection Geography | National |
|  | Data Source(s) | Secondary survey data |
| Findings | PCBEO(s) Studied |  |
|  | Indirect impacts | Unpaid time off work, early retirement |
|  | Intangible burdens | Hindered career or education advancement |
|  | Population-specific Justification Included | No |

| **Haakenstad, A., Hawkins, S. S., Pace, L. E., & Cohen, J. (2019). Rural-urban disparities in colonoscopies after the elimination of patient cost-sharing by the Affordable Care Act. *Preventive Medicine*, *129*, 105877.** | | |
| --- | --- | --- |
| Study Characteristics | Population(s) of Interest | Rural |
|  | Study Population and Sample Size | 358,019 Patients (73,344 Rural) |
|  | Medical Condition(s) | Colonoscopy |
|  | Study Purpose | To assess whether the ACA was associated with changes in rural-urban disparities in OOP costs and colonoscopy rates in the state of Maine, the most rural state in the US. |
|  | Population(s) of interest are focus of the research | Yes |
|  | PCBEOs are a focus of the research | No |
|  | Data Collection Geography | Multiple sites in a single state |
|  | Data Source(s) | Medical claims |
| Findings | PCBEO(s) Studied |  |
|  | Direct medical costs | Copayments, coinsurance, deductibles, premiums |
|  | Population-specific Justification Included | No |

| **Gibson, A., Holmes, S. D., Fields, N. L., & Richardson, V. E. (2019). Providing care for persons with dementia in rural communities: Informal caregivers’ perceptions of supports and services. *Journal of Gerontological Social Work*, *62*(6), 630–648.** | | |
| --- | --- | --- |
| Study Characteristics | Population(s) of Interest | Rural |
|  | Study Population and Sample Size | 11 Caregivers |
|  | Medical Condition(s) | Dementia |
|  | Study Purpose | To study caregivers’ perceptions of knowledge, access, and intent related to caregiving services for rural dementia caregivers. |
|  | Population(s) of interest are focus of the research | Yes |
|  | PCBEOs are a focus of the research | No |
|  | Data Collection Geography | Multiple sites across multiple states |
|  | Data Source(s) | Interviews |
| Findings | PCBEO(s) Studied |  |
|  | Direct medical costs | Diagnosis/treatment/management costs (beyond cost sharing) |
|  | Intangible burdens | Diminished household wealth, Distress, stress, and anxiety directly caused by OOP costs, financial debt, or lost income (i.e., financial toxicity) |
|  | Population-specific Justification Included | Yes |
|  | Justification(s) | Predisposing factor(s): Religious identity; Enabling factor(s): Income, Partner and social support or total help received from relatives; Gaps in the literature |

| **Davis, M. L., Hendrickson, J., Wilson, N., Shrestha, S., Amspoker, A. B., & Kunik, M. (2019). Taking care of the dyad: Frequency of caregiver assessment among veterans with dementia. *Journal of the American Geriatrics Society*, *67*(8), 1604–1609.** | | |
| --- | --- | --- |
| Study Characteristics | Population(s) of Interest | Veteran |
|  | Study Population and Sample Size | 211 Caregivers |
|  | Medical Condition(s) | Dementia |
|  | Study Purpose | To explore the roles and needs of caregivers for veterans with dementia. |
|  | Population(s) of interest are focus of the research | Yes |
|  | PCBEOs are a focus of the research | No |
|  | Data Collection Geography | Single institution/site |
|  | Data Source(s) | Medical records/EHRs |
| Findings | PCBEO(s) Studied |  |
|  | Intangible burdens | General or composite measure of caregiver burden |
|  | Population-specific Justification Included | No |

| **Candon, M. K., Barry, C. L., Marcus, S. C., Epstein, A. J., Kennedy-Hendricks, A., Ming, X., & Mandell, D. S. (2019). Insurance mandates and out-of-pocket spending for children with autism spectrum disorder. *Pediatrics*, *143*(1), 1–7.** | | |
| --- | --- | --- |
| Study Characteristics | Population(s) of Interest | IDD |
|  | Study Population and Sample Size | 106,977 Patients |
|  | Medical Condition(s) | ASD |
|  | Study Purpose | To estimate the effects of state-level ASD insurance mandates on OOP spending (which includes copays, coinsurance, and deductibles), insurer spending, and the share of total spending paid out |
|  | Population(s) of interest are focus of the research | Yes |
|  | PCBEOs are a focus of the research | Yes |
|  | Data Collection Geography | National |
|  | Data Source(s) | Medical claims |
| Findings | PCBEO(s) Studied |  |
|  | Direct medical costs | Copayments, coinsurance, deductibles, premiums |
|  | Population-specific Justification Included | Yes |
|  | Justification(s) | Policy factors |

| **Brickell, T. A., French, L. M., Gartner, R. L., Driscoll, A. E., Wright, M. M., Lippa, S. M., & Lange, R. T. (2019). Factors related to perceived burden among caregivers of service members/veterans following TBI. *Rehabilitation Psychology*, *64*(3), 307–319.** | | |
| --- | --- | --- |
| Study Characteristics | Population(s) of Interest | Veteran |
|  | Study Population and Sample Size | 214 Caregivers |
|  | Medical Condition(s) | TBI |
|  | Study Purpose | To examine factors related to perceived caregiving burden in a sample of caregivers assisting service members and veterans (SMVs) across four areas: SMV injury and health status; caregiver life circumstances; caregiver duty and responsibilities; and caregiver needs. |
|  | Population(s) of interest are focus of the research | Yes |
|  | PCBEOs are a focus of the research | Yes |
|  | Data Collection Geography | Single institution/site |
|  | Data Source(s) | Primary survey data |
| Findings | PCBEO(s) Studied |  |
|  | Direct medical costs | OOP expenses |
|  | Indirect impacts | Time spent providing unpaid caregiving |
|  | Intangible burdens | Hindered career or education advancement |
|  | Population-specific Justification Included | Yes |
|  | Justification(s) | Need factor(s): Chronic illnesses; Gaps in the literature; Policy factors |

| **Benevides, T. W., Lee, J., Nwosu, N. A. O., & Franks, J. (2019). Understanding the family impact of autism spectrum disorder in a racially and ethnically diverse sample: Findings from the National Survey of Children with Special Health Care Needs. *Maternal and Child Health Journal*, *23*(7), 951–960.** | | |
| --- | --- | --- |
| Study Characteristics | Population(s) of Interest | IDD |
|  | Study Population and Sample Size | 5,115 Caregivers |
|  | Medical Condition(s) | ASD |
|  | Study Purpose | To examine differences in family impact variables among caregivers of ASD children from different racial/ethnic backgrounds. |
|  | Population(s) of interest are focus of the research | Yes |
|  | PCBEOs are a focus of the research | Yes |
|  | Data Collection Geography | National |
|  | Data Source(s) | Primary survey data |
| Findings | PCBEO(s) Studied |  |
|  | Direct medical costs | Spending on child's care |
|  | Indirect impacts | Time spent providing unpaid caregiving, Unpaid time off work, early retirement |
|  | Intangible burdens | Caregiver perceived financial problems due to child's condition |
|  | Population-specific Justification Included | Yes |
|  | Justification(s) | Need factor(s): Disability status; Gaps in the literature |

| **Barry, C. L., Kennedy-Hendricks, A., Mandell, D., Epstein, A. J., Candon, M., & Eisenberg, M. (2019). State mandate laws for autism coverage and high-deductible health plans. *Pediatrics*, *143*(6).** | | |
| --- | --- | --- |
| Study Characteristics | Population(s) of Interest | IDD |
|  | Study Population and Sample Size | 98,639 Patients |
|  | Medical Condition(s) | ASD |
|  | Study Purpose | To understand how mandates affect children with ASD in HDHPs relative to traditional, low deductible plans. |
|  | Population(s) of interest are focus of the research | Yes |
|  | PCBEOs are a focus of the research | Yes |
|  | Data Collection Geography | National |
|  | Data Source(s) | Medical claims |
| Findings | PCBEO(s) Studied |  |
|  | Direct medical costs | OOP costs |
|  | Population-specific Justification Included | Yes |
|  | Justification(s) | Gaps in the literature; Policy factors |

| **Schaible, B., Colquitt, G., Caciula, M. C., Carnes, A., Li, L., & Moreau, N. (2018). Comparing impact on the family and insurance coverage in children with cerebral palsy and children with another special healthcare need. *Child: Care, Health, and Devopment*, *44*(3), 370–377.** | | |
| --- | --- | --- |
| Study Characteristics | Population(s) of Interest | IDD |
|  | Study Population and Sample Size | 40,227 Caregivers (744 IDD) |
|  | Medical Condition(s) | Other special healthcare need, Cerebral Palsy |
|  | Study Purpose | To identify factors associated with differences in insurance coverage and impact on the family of children with cerebral palsy and other children with special healthcare needs. |
|  | Population(s) of interest are focus of the research | Yes |
|  | PCBEOs are a focus of the research | Yes |
|  | Data Collection Geography | National |
|  | Data Source(s) | Secondary survey data |
| Findings | PCBEO(s) Studied |  |
|  | Direct medical costs | Total OOP expenses for healthcare |
|  | Indirect impacts | Time spent providing unpaid caregiving |
|  | Intangible burdens | Changes in employment status and/or insurance coverage |
|  | Population-specific Justification Included | Yes |
|  | Justification(s) | Enabling factor(s): Income; Need factor(s): Disability status |

| **Poyner-Del Vento, P., Goy, E., Baddeley, J., & Libet, J. (2018). The caregivers’ attachment and relationship education class: A new and promising group therapy for caregivers of individuals with Parkinson’s disease. *Journal of Couple & Relationship Therapy*, *17*(2), 97–113.** | | |
| --- | --- | --- |
| Study Characteristics | Population(s) of Interest | Veteran |
|  | Study Population and Sample Size | 7 Caregivers |
|  | Medical Condition(s) | Parkinson's disease |
|  | Study Purpose | To examine whether a newly developed group therapy, based on principles of attachment theory and emotionally focused couples therapy, can potentially address the unique mental health and relationship difficulties in caregivers of individuals with Parkinson’s disease |
|  | Population(s) of interest are focus of the research | No |
|  | PCBEOs are a focus of the research | No |
|  | Data Collection Geography | Single institution/site |
|  | Data Source(s) | Primary survey data |
| Findings | PCBEO(s) Studied |  |
|  | Intangible burdens | General or composite measure of caregiver burden |
|  | Population-specific Justification Included | No |

| **Little, L. M., Wallisch, A., Pope, E., & Dunn, W. (2018). Acceptability and cost comparison of a telehealth intervention for families of children with autism. *Infants & Young Children: An Interdisciplinary Journal of Early Childhood Intervention*, *31*(4), 275–286.** | | |
| --- | --- | --- |
| Study Characteristics | Population(s) of Interest | IDD |
|  | Study Population and Sample Size | 18 Patients, 34 Caregivers |
|  | Medical Condition(s) | ASD |
|  | Study Purpose | To investigate the acceptability (n = 17) and cost-effectiveness (n = 18) of a 12-week telehealth intervention among families of young children with ASD. |
|  | Population(s) of interest are focus of the research | No |
|  | PCBEOs are a focus of the research | No |
|  | Data Collection Geography | Not stated/unclear |
|  | Data Source(s) | Primary survey data |
| Findings | PCBEO(s) Studied |  |
|  | Direct non-medical costs | Transportation, meals, hoteling |
|  | Indirect impacts | Time to attend medical appointments, Time spent traveling to or between medical appointments, Unpaid time off work, early retirement; Time spent preparing for appointments |
|  | Population-specific Justification Included | Yes |
|  | Justification(s) | Enabling factor(s): Rurality, Shortage of providers; Need factor(s): Disability status |

| **Kayadjanian, N., Schwartz, L., Farrar, E., Comtois, K. A., & Strong, T. V. (2018). High levels of caregiver burden in Prader-Willi syndrome. *PLoS One*, *13*(3), e0194655.** | | |
| --- | --- | --- |
| Study Characteristics | Population(s) of Interest | IDD |
|  | Study Population and Sample Size | 142 Caregivers |
|  | Medical Condition(s) | PWS |
|  | Study Purpose | To measure the level of burden in caregivers of individuals with PWS, to explore the impact of PWS on caregiver quality of life, and to assess ZBI as an indicator of that impact. |
|  | Population(s) of interest are focus of the research | Yes |
|  | PCBEOs are a focus of the research | Yes |
|  | Data Collection Geography | National |
|  | Data Source(s) | Primary survey data |
| Findings | PCBEO(s) Studied |  |
|  | Intangible burdens | General or composite measure of caregiver burden |
|  | Population-specific Justification Included | Yes |
|  | Justification(s) | Need factor(s): Chronic illnesses, Disability status |

| **Hooshmand, M., & Foronda, C. (2018). Comparison of telemedicine to traditional face-to-face care for children with special needs: A quasiexperimental study. *Telemedicine Journal and E-Health*, *24*(6), 433–441.** | | |
| --- | --- | --- |
| Study Characteristics | Population(s) of Interest | Rural, IDD |
|  | Study Population and Sample Size | 178 Caregivers |
|  | Medical Condition(s) | Children with special healthcare needs |
|  | Study Purpose | To examine cost, caring, and family-centered care in relationship to pediatric specialty services integrating telemedicine visits compared to traditional face-to-face visits only for (CSHCN) in rural, remote, and medically underserved areas. |
|  | Population(s) of interest are focus of the research | Yes |
|  | PCBEOs are a focus of the research | Yes |
|  | Data Collection Geography | Multiple sites in a single state |
|  | Data Source(s) | Primary survey data |
| Findings | PCBEO(s) Studied |  |
|  | Direct medical costs | Copayments, coinsurance, deductibles, premiums |
|  | Direct non-medical costs | Child/dependent care costs, Transportation, meals, hoteling |
|  | Indirect impacts | Time spent traveling to or between medical appointments, Unpaid time off work, early retirement |
|  | Population-specific Justification Included | No |

| **Hand, B. N., Lane, A. E., De Boeck, P., Basso, D. M., Nichols-Larsen, D. S., & Darragh, A. R. (2018). Caregiver burden varies by sensory subtypes and sensory dimension scores of children with autism. *Journal of Autism and Developmental Disorders*, *48*(4), 1133–1146.** | | |
| --- | --- | --- |
| Study Characteristics | Population(s) of Interest | IDD |
|  | Study Population and Sample Size | 367 Caregivers |
|  | Medical Condition(s) | ASD |
|  | Study Purpose | To explore the association between child sensory subtype, sensory dimension scores, and caregiver burden. |
|  | Population(s) of interest are focus of the research | Yes |
|  | PCBEOs are a focus of the research | Yes |
|  | Data Collection Geography | National |
|  | Data Source(s) | Primary survey data |
| Findings | PCBEO(s) Studied |  |
|  | Intangible burdens | General or composite measure of caregiver burden |
|  | Population-specific Justification Included | Yes |
|  | Justification(s) | Need factor(s): Chronic illnesses, Disability status; Gaps in the literature |

| **Dovgan, K. N., & Mazurek, M. O. (2018). Differential effects of child difficulties on family burdens across diagnostic groups. *Journal of Child & Family Studies*, *27*(3), 872–884.** | | |
| --- | --- | --- |
| Study Characteristics | Population(s) of Interest | IDD |
|  | Study Population and Sample Size | 10,649 Caregivers |
|  | Medical Condition(s) | ADHD, ASD |
|  | Study Purpose | To investigate the nature of family burdens by examining the effects of child characteristics, family characteristics, and child difficulties. |
|  | Population(s) of interest are focus of the research | Yes |
|  | PCBEOs are a focus of the research | Yes |
|  | Data Collection Geography | National |
|  | Data Source(s) | Secondary survey data |
| Findings | PCBEO(s) Studied |  |
|  | Indirect impacts | Time spent providing unpaid caregiving, Unpaid time off work, early retirement |
|  | Intangible burdens | Distress, stress, and anxiety directly caused by OOP costs, financial debt, or lost income (i.e., financial toxicity), General or composite measure of caregiver burden |
|  | Population-specific Justification Included | Yes |
|  | Justification(s) | Need factor(s): Disability status; Gaps in the literature |

| **Delgado, R. E., Peacock, K., Elizondo, B., Wells, M., Grafman, J. H., & Pugh, M. J. (2018). A family’s affair: Caring for veterans with penetrating traumatic brain injury. *Military Medicine*, *183*(3–4, Suppl), 379–385.** | | |
| --- | --- | --- |
| Study Characteristics | Population(s) of Interest | Veteran |
|  | Study Population and Sample Size | 65 Caregivers |
|  | Medical Condition(s) | TBI |
|  | Study Purpose | To assess the health-related outcomes of post 9/11 veterans with penetrating traumatic brain injury (pTBI) by examining the health impact of caregiving on caregivers. |
|  | Population(s) of interest are focus of the research | Yes |
|  | PCBEOs are a focus of the research | No |
|  | Data Collection Geography | Not stated/unclear |
|  | Data Source(s) | Primary survey data, Medical claims |
| Findings | PCBEO(s) Studied |  |
|  | Intangible burdens | General or composite measure of caregiver burden |
|  | Population-specific Justification Included | Yes |
|  | Justification(s) | Need factor(s): Chronic illnesses, Disability status; Gaps in the literature |

| **Choi, N. G., & DiNitto, D. M. (2018). Correlates of worry about health care costs among older adults. *Journal of Applied Gerontology*, *37*(6), 763–782.** | | |
| --- | --- | --- |
| Study Characteristics | Population(s) of Interest | Veteran |
|  | Study Population and Sample Size | 7,253 Patients (548 Veteran) |
|  | Medical Condition(s) | Old age |
|  | Study Purpose | To examine factors associated with older adults’ health care cost worries, defined as at least a moderate level of worry, aboutability to pay for normal health care and/or for health care due to a serious illness or accident. |
|  | Population(s) of interest are focus of the research | No |
|  | PCBEOs are a focus of the research | Yes |
|  | Data Collection Geography | National |
|  | Data Source(s) | Secondary survey data |
| Findings | PCBEO(s) Studied |  |
|  | Intangible burdens | Distress, stress, and anxiety directly caused by OOP costs, financial debt, or lost income (i.e., financial toxicity) |
|  | Population-specific Justification Included | No |

| **Black, L. I., & Zablotsky, B. (2018). Chronic school absenteeism among children with selected developmental disabilities: National Health Interview Survey, 2014-2016. *National Health Statistics Reports*, *118*, 1–7.** | | |
| --- | --- | --- |
| Study Characteristics | Population(s) of Interest | IDD |
|  | Study Population and Sample Size | 26,458 Patients |
|  | Medical Condition(s) | ASD |
|  | Study Purpose | To describe associations between chronic school absenteeism and selected developmental disabilities (DDs) among school-aged children. |
|  | Population(s) of interest are focus of the research | Yes |
|  | PCBEOs are a focus of the research | Yes |
|  | Data Collection Geography | National |
|  | Data Source(s) | Secondary survey data |
| Findings | PCBEO(s) Studied |  |
|  | Indirect impacts | School absenteeism |
|  | Population-specific Justification Included | Yes |
|  | Justification(s) | Need factor(s): Chronic illnesses, Disability status |

| **Vessey, J. A., DiFazio, R. L., Strout, T. D., & Snyder, B. D. (2017). Impact of non-medical out-of-pocket expenses on families of children with cerebral palsy following orthopaedic surgery. *Journal of Pediatric Nursing*, *37*, 101–107.** | | |
| --- | --- | --- |
| Study Characteristics | Population(s) of Interest | IDD |
|  | Study Population and Sample Size | 52 Caregivers |
|  | Medical Condition(s) | Cerebral Palsy |
|  | Study Purpose | To: 1) calculate the estimated total of non-medical OOP rxpenses (NOOPEs) incurred by families during the hospitalization of their child as measured by the Family Expense Diary, expressed within the context of the percent of the family's annual income, 2) assess the impact of the child's chronic condition on the family's finances through financial impact self-report measures, and 3) identify predictors that contribute to incurring high NOOPEs. |
|  | Population(s) of interest are focus of the research | Yes |
|  | PCBEOs are a focus of the research | Yes |
|  | Data Collection Geography | Single institution/site |
|  | Data Source(s) | Primary survey data |
| Findings | PCBEO(s) Studied |  |
|  | Direct non-medical costs | Child/dependent care costs, Transportation, meals, hoteling; Composite financial burden |
|  | Indirect impacts | Unpaid time off work, early retirement |
|  | Population-specific Justification Included | Yes |
|  | Justification(s) | Need factor(s): Disability status; Gaps in the literature |

| **Stuart, E. A., McGinty, E. E., Kalb, L., Huskamp, H. A., Busch, S. H., Gibson, T. B., Goldman, H., & Barry, C. L. (2017). Increased service use among children with autism spectrum disorder associated with mental health parity law. *Health Affairs*, *36*(2), 337–345.** | | |
| --- | --- | --- |
| Study Characteristics | Population(s) of Interest | IDD |
|  | Study Population and Sample Size | 48,164 Patients |
|  | Medical Condition(s) | ASD |
|  | Study Purpose | To examine whether the Mental Health Parity and Addiction Equity Act was associated with increased use of and spending on mental health care and functional services for children with ASD compared to the period prior to implementation of the law. |
|  | Population(s) of interest are focus of the research | Yes |
|  | PCBEOs are a focus of the research | No |
|  | Data Collection Geography | National |
|  | Data Source(s) | Medical claims |
| Findings | PCBEO(s) Studied |  |
|  | Direct medical costs | Copayments, coinsurance, deductibles, premiums, Diagnosis/treatment/management costs (beyond cost sharing), Medication costs |
|  | Population-specific Justification Included | Yes |
|  | Justification(s) | Need factor(s): Disability status; Policy factors |

| **Schehlein, E. M., Im, L. T., Robin, A. L., Onukwugha, E., & Saeedi, O. J. (2017). Nonmedical out-of-pocket patient and companion expenditures associated with glaucoma care. *Journal of Glaucoma*, *26*(4), 343–348.** | | |
| --- | --- | --- |
| Study Characteristics | Population(s) of Interest | Rural |
|  | Study Population and Sample Size | 300 Patients (24 Rural) |
|  | Medical Condition(s) | Glaucoma |
|  | Study Purpose | To evaluate potential key predictors of patient expenditures that are critical to assessing the cost-effectiveness of glaucoma health care delivery. |
|  | Population(s) of interest are focus of the research | No |
|  | PCBEOs are a focus of the research | Yes |
|  | Data Collection Geography | Multiple sites across multiple states |
|  | Data Source(s) | Primary survey data |
| Findings | PCBEO(s) Studied |  |
|  | Direct medical costs | Composite visit expenditure |
|  | Population-specific Justification Included | No |

| **Raymaker, D. M., McDonald, K. E., Ashkenazy, E., Gerrity, M., Baggs, A. M., Kripke, C., Hourston, S., & Nicolaidis, C. (2017). Barriers to healthcare: Instrument development and comparison between autistic adults and adults with and without other disabilities. *Autism: The International Journal of Research & Practice*, *21*(8), 972–984.** | | |
| --- | --- | --- |
| Study Characteristics | Population(s) of Interest | IDD |
|  | Study Population and Sample Size | 437 Patients (209 IDD) |
|  | Medical Condition(s) | General Disability, ASD |
|  | Study Purpose | To use a community-based participatory research approach to identify and compare barriers to healthcare experienced by autistic adults and adults with and without other disabilities. |
|  | Population(s) of interest are focus of the research | Yes |
|  | PCBEOs are a focus of the research | Yes |
|  | Data Collection Geography | National |
|  | Data Source(s) | Primary survey data |
| Findings | PCBEO(s) Studied |  |
|  | Direct medical costs | Copayments, coinsurance, deductibles, premiums |
|  | Direct non-medical costs | Transportation, meals, hoteling |
|  | Indirect impacts | Time to attend medical appointments |
|  | Intangible burdens | Delaying or forgoing medical treatments due to costs |
|  | Population-specific Justification Included | Yes |
|  | Justification(s) | Enabling factor(s): Barriers to healthcare; Gaps in the literature; Policy factors |

| **Hillier, A., Galizzi, M., & Ferrante, K. (2017). Healthcare experiences of young adults with autism spectrum disorder. *Advances in Autism*, *3*(4), 206–219.** | | |
| --- | --- | --- |
| Study Characteristics | Population(s) of Interest | IDD |
|  | Study Population and Sample Size | 16 Patients, 50 Caregivers |
|  | Medical Condition(s) | ASD |
|  | Study Purpose | To examine the healthcare experiences of young adults with ASD within the US healthcare system. |
|  | Population(s) of interest are focus of the research | Yes |
|  | PCBEOs are a focus of the research | No |
|  | Data Collection Geography | National |
|  | Data Source(s) | Primary survey data |
| Findings | PCBEO(s) Studied |  |
|  | Direct medical costs | Copayments, coinsurance, deductibles, premiums, Medication costs |
|  | Direct non-medical costs | Transportation, meals, hoteling |
|  | Indirect impacts | Time spent providing unpaid caregiving, Unpaid time off work, early retirement |
|  | Population-specific Justification Included | Yes |
|  | Justification(s) | Gaps in the literature |

| **Gupte-Singh, K., Singh, R. R., & Lawson, K. A. (2017). Economic burden of attention-deficit/hyperactivity disorder among pediatric patients in the United States. *Value in Health*, *20*(4), 602–609.** | | |
| --- | --- | --- |
| Study Characteristics | Population(s) of Interest | IDD |
|  | Study Population and Sample Size | 9,101 Patients (458 IDD) |
|  | Medical Condition(s) | ADHD |
|  | Study Purpose | To determine the adjusted incremental total costs (direct and indirect) for patients (aged 3–17 years) with attention-deficit/ hyperactivity disorder (ADHD) and the differences in the adjusted incremental direct expenditures with respect to age groups (preschoolers, 0–5 years; children, 6–11 years; and adolescents, 12–17 years). |
|  | Population(s) of interest are focus of the research | Yes |
|  | PCBEOs are a focus of the research | Yes |
|  | Data Collection Geography | National |
|  | Data Source(s) | Secondary survey data |
| Findings | PCBEO(s) Studied |  |
|  | Direct medical costs | Copayments, coinsurance, deductibles, premiums, Medical transportation costs (e.g., ambulance), Medication costs |
|  | Indirect impacts | Time spent providing unpaid caregiving, Unpaid time off work, early retirement |
|  | Population-specific Justification Included | Yes |
|  | Justification(s) | Enabling factors; Need factor(s): Disability status |

| **Griffin, J. M., Lee, M. K., Bangerter, L. R., Van Houtven, C. H., Friedemann-Sánchez, G., Phelan, S. M., Carlson, K. F., & Meis, L. A. (2017). Burden and mental health among caregivers of veterans with traumatic brain injury/polytrauma. *American Journal of Orthopsychiatry*, *87*(2), 139–148.** | | |
| --- | --- | --- |
| Study Characteristics | Population(s) of Interest | Veteran |
|  | Study Population and Sample Size | 564 Caregivers |
|  | Medical Condition(s) | TBI |
|  | Study Purpose | To 1) examine the relationship between caregiver stressors, namely veterans’ persistent neurobehavioral symptoms associated with their injuries and the intensity of care they require and caregiver well-being, measured by caregiver burden and mental health and 2) assess how intrapersonal, social, family, and financial resources—potential targets for intervention— explain or modify the relationship between caregiver stress and well-being. |
|  | Population(s) of interest are focus of the research | Yes |
|  | PCBEOs are a focus of the research | Yes |
|  | Data Collection Geography | National |
|  | Data Source(s) | Primary survey data |
| Findings | PCBEO(s) Studied |  |
|  | Intangible burdens | General or composite measure of caregiver burden |
|  | Population-specific Justification Included | Yes |
|  | Justification(s) | Need factor(s): Perceived general health status; Gaps in the literature |

| **dosReis, S., Park, A., Ng, X., Frosch, E., Reeves, G., Cunningham, C., Janssen, E. M., & Bridges, J. F. P. (2017). Caregiver treatment preferences for children with a new versus existing attention-deficit/hyperactivity disorder diagnosis. *Journal of Child and Adolescent Psychopharmacology*, *27*(3), 234–242.** | | |
| --- | --- | --- |
| Study Characteristics | Population(s) of Interest | IDD |
|  | Study Population and Sample Size | 184 Caregivers |
|  | Medical Condition(s) | ADHD |
|  | Study Purpose | To identify the ADHD management options caregivers most prefer and to determine if preferences differ by time since initial ADHD diagnosis. |
|  | Population(s) of interest are focus of the research | Yes |
|  | PCBEOs are a focus of the research | Yes |
|  | Data Collection Geography | Multiple sites in a single state |
|  | Data Source(s) | Primary survey data |
| Findings | PCBEO(s) Studied |  |
|  | Direct medical costs | Composite direct OOP costs |
|  | Indirect impacts | Composite OOP indirect costs |
|  | Population-specific Justification Included | No |

| **Doshi, P., Tilford, J. M., Ounpraseuth, S., Kuo, D. Z., & Payakachat, N. (2017). Do insurance mandates affect racial disparities in outcomes for children with autism? *Maternal and Child Health Journal*, *21*(2), 351–366.** | | |
| --- | --- | --- |
| Study Characteristics | Population(s) of Interest | IDD |
|  | Study Population and Sample Size | 5,178 Caregivers |
|  | Medical Condition(s) | ASD |
|  | Study Purpose | To investigate whether state mandates for private insurers to provide services for children with autism influence racial disparities in outcomes. |
|  | Population(s) of interest are focus of the research | Yes |
|  | PCBEOs are a focus of the research | Yes |
|  | Data Collection Geography | National |
|  | Data Source(s) | Secondary survey data |
| Findings | PCBEO(s) Studied |  |
|  | Direct medical costs | Composite OOP costs |
|  | Indirect impacts | Time spent providing unpaid caregiving, Unpaid time off work, early retirement |
|  | Intangible burdens | Distress, stress, and anxiety directly caused by OOP costs, financial debt, or lost income (i.e., financial toxicity) |
|  | Population-specific Justification Included | No |

| **Crouch, E., Probst, J., & Bennett, K. (2017). Rural-urban differences in unpaid caregivers of adults. *Rural Remote Health*, *17*(4), 4351.** | | |
| --- | --- | --- |
| Study Characteristics | Population(s) of Interest | Rural |
|  | Study Population and Sample Size | 1,248 Caregivers |
|  | Medical Condition(s) | Old age, Medical condition not specified |
|  | Study Purpose | To explore rural–urban differences in the characteristics of unpaid caregivers of adults in the USA. |
|  | Population(s) of interest are focus of the research | Yes |
|  | PCBEOs are a focus of the research | Yes |
|  | Data Collection Geography | National |
|  | Data Source(s) | Secondary survey data |
| Findings | PCBEO(s) Studied |  |
|  | Indirect impacts | Time spent providing unpaid caregiving |
|  | Intangible burdens | Distress, stress, and anxiety directly caused by OOP costs, financial debt, or lost income (i.e., financial toxicity), General or composite measure of caregiver burden |
|  | Population-specific Justification Included | Yes |
|  | Justification(s) | Enabling factor(s): Income, Rurality, Distance to the nearest medical institution, Partner and social support or total help received from relatives; Policy factors |

| **Zhang, W., & Baranek, G. (2016). The impact of insurance coverage types on access to and utilization of health services for U.S. children with autism. *Psychiatric Services*, *67*(8), 908–911.** | | |
| --- | --- | --- |
| Study Characteristics | Population(s) of Interest | IDD |
|  | Study Population and Sample Size | 2,041 Patients |
|  | Medical Condition(s) | ASD |
|  | Study Purpose | To examine the association of insurance type with access to and utilization of essential health services among children with autism spectrum disorder (ASD). |
|  | Population(s) of interest are focus of the research | Yes |
|  | PCBEOs are a focus of the research | No |
|  | Data Collection Geography | National |
|  | Data Source(s) | Secondary survey data |
| Findings | PCBEO(s) Studied |  |
|  | Direct medical costs | OOP costs |
|  | Population-specific Justification Included | No |

| **Wiener, R. C., Vohra, R., Sambamoorthi, U., & Madhavan, S. S. (2016). Caregiver burdens and preventive dental care for children with autism spectrum disorder, developmental disability and/or mental health conditions: National Survey of CSHCN, 2009-2010. *Maternal and Child Health Journal*, *20*(12), 2573–2580.** | | |
| --- | --- | --- |
| Study Characteristics | Population(s) of Interest | IDD |
|  | Study Population and Sample Size | 16,323 Caregivers |
|  | Medical Condition(s) | ASD |
|  | Study Purpose | To examine the burdens of caregivers on perception of the need and receipt of preventive dental care for a subset of children with special health care needs—children with Autism Spectrum disorder, developmental disability and/or mental health conditions (CASD/DD/MHC). |
|  | Population(s) of interest are focus of the research | Yes |
|  | PCBEOs are a focus of the research | Yes |
|  | Data Collection Geography | National |
|  | Data Source(s) | Secondary survey data |
| Findings | PCBEO(s) Studied |  |
|  | Direct medical costs | Dollars spent per year on healthcare |
|  | Indirect impacts | Time spent providing unpaid caregiving, Unpaid time off work, early retirement |
|  | Population-specific Justification Included | Yes |
|  | Justification(s) | Need factor(s): Disability status; Gaps in the literature; Policy factors |

| **Thomas, K. C., Williams, C. S., deJong, N., & Morrissey, J. P. (2016). Examination of parent insurance ratings, child expenditures, and financial burden among children with autism: A mismatch suggests new hypotheses to test. *Pediatrics*, 137 Suppl 2, S186–S195.** | | |
| --- | --- | --- |
| Study Characteristics | Population(s) of Interest | IDD |
|  | Study Population and Sample Size | 346 Unspecified participants |
|  | Medical Condition(s) | ASD |
|  | Study Purpose | To examine health insurance ratings from parents raising children with autism and child expenditures to explore how these measures align. |
|  | Population(s) of interest are focus of the research | Yes |
|  | PCBEOs are a focus of the research | No |
|  | Data Collection Geography | National |
|  | Data Source(s) | Secondary survey data |
| Findings | PCBEO(s) Studied |  |
|  | Direct medical costs | Copayments, coinsurance, deductibles, premiums, Medication costs |
|  | Population-specific Justification Included | Yes |
|  | Justification(s) | Gaps in the literature |

| **Smalley, K. B., Warren, J. C., & Barefoot, K. N. (2016). Connection between depression and inability to fill prescriptions in rural FQHC patients with chronic disease. *Journal of Rural Mental Health*, *40*(2), 113–123.** | | |
| --- | --- | --- |
| Study Characteristics | Population(s) of Interest | Rural |
|  | Study Population and Sample Size | 497 Patients |
|  | Medical Condition(s) | Diabetes, hypertension |
|  | Study Purpose | To (a) examine the rates of elevated depression symptoms among a sample of rural Federally Qualified Health Center (FQHC) patients with chronic disease, and (b) determine if an inability to afford general prescription medications within the past 12 months is a significant predictor of depression symptoms among these patients. |
|  | Population(s) of interest are focus of the research | Yes |
|  | PCBEOs are a focus of the research | No |
|  | Data Collection Geography | Multiple sites across multiple states |
|  | Data Source(s) | Secondary survey data |
| Findings | PCBEO(s) Studied |  |
|  | Intangible burdens | Delaying or forgoing medical treatments due to costs |
|  | Population-specific Justification Included | Yes |
|  | Justification(s) | Enabling factor(s): Income, Rurality; Gaps in the literature |

| **Saban, K. L., Griffin, J. M., Urban, A., Janusek, M. A., Pape, T. L. B., & Collins, E. (2016). Perceived health, caregiver burden, and quality of life in women partners providing care to veterans with traumatic brain injury. *Journal of Rehabilitation Research and Development*, *53*(6), 681–691.** | | |
| --- | --- | --- |
| Study Characteristics | Population(s) of Interest | Veteran |
|  | Study Population and Sample Size | 40 Caregivers |
|  | Medical Condition(s) | TBI |
|  | Study Purpose | To describe perceived health, somatic symptoms, caregiver burden, and perceived QOL and to identify the extent to which these variables are associated with QOL in female partners/spouses of Veterans with TBI. |
|  | Population(s) of interest are focus of the research | Yes |
|  | PCBEOs are a focus of the research | Yes |
|  | Data Collection Geography | National |
|  | Data Source(s) | Primary survey data |
| Findings | PCBEO(s) Studied |  |
|  | Indirect impacts | Time spent providing unpaid caregiving |
|  | Intangible burdens | Distress, stress, and anxiety directly caused by OOP costs, financial debt, or lost income (i.e., financial toxicity), General or composite measure of caregiver burden |
|  | Population-specific Justification Included | Yes |
|  | Justification(s) | Need factor(s): Chronic illnesses; Gaps in the literature |

| **Raspa, M., Sacco, P., Candrilli, S. D., Bishop, E., & Petrillo, J. (2016). Validity of a condition specific outcome measure for fragile X syndrome: The Aberrant Behaviour Checklist-utility index. *Journal of Intellectual Disability Research*, *60*(9), 844–855.** | | |
| --- | --- | --- |
| Study Characteristics | Population(s) of Interest | IDD |
|  | Study Population and Sample Size | 350 Caregivers |
|  | Medical Condition(s) | Fragile X Syndrome |
|  | Study Purpose | To assess the construct validity of the Aberrant Behaviour Checklist-utility index (ABC-UI) by examining the relationship between healthcare resource utilization by patients with fragile X syndrome (FXS) as well as burden experienced by their caregivers. |
|  | Population(s) of interest are focus of the research | Yes |
|  | PCBEOs are a focus of the research | No |
|  | Data Collection Geography | National |
|  | Data Source(s) | Primary survey data |
| Findings | PCBEO(s) Studied |  |
|  | Direct non-medical costs | Child/dependent care costs |
|  | Indirect impacts | Time spent providing unpaid caregiving |
|  | Intangible burdens | Changes in employment status and/or insurance coverage, Distress, stress, and anxiety directly caused by OOP costs, financial debt, or lost income (i.e., financial toxicity) |
|  | Population-specific Justification Included | Yes |
|  | Justification(s) | Need factor(s): Chronic illnesses, Disability status |

| **Nichols, L. O., Martindale-Adams, J., Burns, R., Zuber, J., & Graney, M. J. (2016). REACH VA: Moving from translation to system implementation... resources for enhancing all caregivers health in the Department of Veterans Affairs. *Gerontologist*, *56*(1), 135–144.** | | |
| --- | --- | --- |
| Study Characteristics | Population(s) of Interest | Veteran |
|  | Study Population and Sample Size | 125 Caregivers |
|  | Medical Condition(s) | Alzheimer's disease |
|  | Study Purpose | To describe the trajectory of REACH VA from national randomized clinical trial through translation to national implementation. |
|  | Population(s) of interest are focus of the research | No |
|  | PCBEOs are a focus of the research | No |
|  | Data Collection Geography | Single institution/site |
|  | Data Source(s) | Primary survey data |
| Findings | PCBEO(s) Studied |  |
|  | Indirect impacts | Time spent providing unpaid caregiving |
|  | Intangible burdens | General or composite measure of caregiver burden |
|  | Population-specific Justification Included | No |

| **Lowes, L., Clark, T. S., & Noritz, G. (2016). Factors associated with caregiver experience in families with a child with cerebral palsy. *Journal of Pediatric Rehabilitation Medicine*, *9*(1), 65–72.** | | |
| --- | --- | --- |
| Study Characteristics | Population(s) of Interest | IDD |
|  | Study Population and Sample Size | 296 Caregivers |
|  | Medical Condition(s) | Cerebral Palsy |
|  | Study Purpose | To identify factors that are associated with higher levels of caregiver stress. |
|  | Population(s) of interest are focus of the research | Yes |
|  | PCBEOs are a focus of the research | Yes |
|  | Data Collection Geography | Single institution/site |
|  | Data Source(s) | Primary survey data |
| Findings | PCBEO(s) Studied |  |
|  | Indirect impacts | Unpaid time off work, early retirement |
|  | Intangible burdens | Distress, stress, and anxiety directly caused by OOP costs, financial debt, or lost income (i.e., financial toxicity), General or composite measure of caregiver burden |
|  | Population-specific Justification Included | No |

| **Haozous, E. A. (2016). Cancer journey for American Indians and Alaska Natives in the Pacific Northwest. *Oncology Nursing Forum*, *43*(5), 625–635.** | | |
| --- | --- | --- |
| Study Characteristics | Population(s) of Interest | Rural |
|  | Study Population and Sample Size | 30 Patients |
|  | Medical Condition(s) | Cancer |
|  | Study Purpose | To describe the experiences of American Indian and Alaska Native cancer survivors to improve understanding of the trajectory of cancer treatment. |
|  | Population(s) of interest are focus of the research | Yes |
|  | PCBEOs are a focus of the research | No |
|  | Data Collection Geography | Multiple sites across multiple states |
|  | Data Source(s) | Focus groups |
| Findings | PCBEO(s) Studied |  |
|  | Direct non-medical costs | Transportation, meals, hoteling |
|  | Population-specific Justification Included | Yes |
|  | Justification(s) | Gaps in the literature |

| **Dyches, T. T., Christensen, R., Harper, J. M., Mandleco, B., & Roper, S. O. (2016). Respite care for single mothers of children with autism spectrum disorders. *Journal of Autism and Developmental Disorders*, *46*(3), 812–824.** | | |
| --- | --- | --- |
| Study Characteristics | Population(s) of Interest | IDD |
|  | Study Population and Sample Size | 122 Caregivers |
|  | Medical Condition(s) | ASD |
|  | Study Purpose | To (a) investigate the perceptions of single mothers of children with ASD regarding their daily stress (as measured by daily hassles and caregiver burden), daily uplifts, depression, and respite care, and (b) to examine the relationship between respite care and depression with stress and uplifts as mediators. |
|  | Population(s) of interest are focus of the research | Yes |
|  | PCBEOs are a focus of the research | No |
|  | Data Collection Geography | National |
|  | Data Source(s) | Primary survey data |
| Findings | PCBEO(s) Studied |  |
|  | Intangible burdens | General or composite measure of caregiver burden |
|  | Population-specific Justification Included | Yes |
|  | Justification(s) | Gaps in the literature; Policy factors |

| **Brioschi Guevara, A., Demonet, J., Polejaeva, E., Knutson, K. M., Wassermann, E. M., Grafman, J., Krueger, F. (2016). Association between traumatic brain injury-related brain lesions and long-term caregiver burden. *Journal of Head Trauma Rehabilitation*, *31*(2), E48–E58.** | | |
| --- | --- | --- |
| Study Characteristics | Population(s) of Interest | Veteran |
|  | Study Population and Sample Size | 128 Patients, 128 Caregivers |
|  | Medical Condition(s) | TBI |
|  | Study Purpose | To investigate the association between traumatic brain injury (TBI) related brain lesions and long-term caregiver burden in relation to dysexecutive syndrome. |
|  | Population(s) of interest are focus of the research | Yes |
|  | PCBEOs are a focus of the research | Yes |
|  | Data Collection Geography | National |
|  | Data Source(s) | Primary survey data |
| Findings | PCBEO(s) Studied |  |
|  | Intangible burdens | General or composite measure of caregiver burden |
|  | Population-specific Justification Included | No |

| **Bashiri, M., Greenfield, L. J., Jr., & Oliveto, A. (2016). Telemedicine interest for routine follow-up care among neurology patients in Arkansas. *Telemedicine Journal and E-Health*, *22*(6), 514–518.** | | |
| --- | --- | --- |
| Study Characteristics | Population(s) of Interest | Rural |
|  | Study Population and Sample Size | 1,441 Patients |
|  | Medical Condition(s) | Neurologic condition |
|  | Study Purpose | To determine patient interest in participating in teleneurology for routine follow-up visits as well as demographic and medical factors associated with interest. |
|  | Population(s) of interest are focus of the research | Yes |
|  | PCBEOs are a focus of the research | No |
|  | Data Collection Geography | Multiple sites in a single state |
|  | Data Source(s) | Primary survey data |
| Findings | PCBEO(s) Studied |  |
|  | Direct non-medical costs | Transportation, meals, hoteling |
|  | Indirect impacts | Time spent traveling to or between medical appointments |
|  | Intangible burdens | Delaying or forgoing medical treatments due to costs, Distress, stress, and anxiety directly caused by OOP costs, financial debt, or lost income (i.e., financial toxicity) |
|  | Population-specific Justification Included | No |

| **Warner, E. L., Kirchhoff, A. C., Nam, G. E., & Fluchel, M. (2015). Financial burden of pediatric cancer for patients and their families. *Journal of Oncology Practice*, *11*(1), 12–18.** | | |
| --- | --- | --- |
| Study Characteristics | Population(s) of Interest | Rural |
|  | Study Population and Sample Size | 254 Caregivers (50 Rural) |
|  | Medical Condition(s) | Cancer |
|  | Study Purpose | To evaluate pediatric cancer caregivers’ perceived financial burden related to socioeconomic factors (eg, parental employment) and health care use factors (e.g., unexpected hospitalizations). |
|  | Population(s) of interest are focus of the research | No |
|  | PCBEOs are a focus of the research | Yes |
|  | Data Collection Geography | Single institution/site |
|  | Data Source(s) | Primary survey data |
| Findings | PCBEO(s) Studied |  |
|  | Direct medical costs | Composite financial burden |
|  | Direct non-medical costs | Composite financial burden |
|  | Intangible burdens | General or composite measure of caregiver burden |
|  | Population-specific Justification Included | No |

| **Saunders, B. S., Tilford, J. M., Fussell, J. J., Schulz, E. G., Casey, P. H., & Kuo, D. Z. (2015). Financial and employment impact of intellectual disability on families of children with autism. *Families, Systems, & Health*, *33*(1), 36–45.** | | |
| --- | --- | --- |
| Study Characteristics | Population(s) of Interest | IDD |
|  | Study Population and Sample Size | 2,406 Caregivers |
|  | Medical Condition(s) | ASD and Other Intellectual Disabilities |
|  | Study Purpose | To describe additional impact on families of children with intellectual disability (ID) in addition to ASD. |
|  | Population(s) of interest are focus of the research | Yes |
|  | PCBEOs are a focus of the research | Yes |
|  | Data Collection Geography | National |
|  | Data Source(s) | Secondary survey data |
| Findings | PCBEO(s) Studied |  |
|  | Direct medical costs | Diagnosis/treatment/management costs (beyond cost sharing) |
|  | Indirect impacts | Time spent providing unpaid caregiving, Unpaid time off work, early retirement |
|  | Intangible burdens | General or composite measure of caregiver burden |
|  | Population-specific Justification Included | Yes |
|  | Justification(s) | Need factor(s): Disability status; Policy factors |

| **Ronis, S. D., Baldwin, C. D., Blumkin, A., Kuhlthau, K., & Szilagyi, P. G. (2015). Patient-centered medical home and family burden in attention-deficit hyperactivity disorder. *Journal of Developmental & Behavioral Pediatrics*, *36*(6), 417–425.** | | |
| --- | --- | --- |
| Study Characteristics | Population(s) of Interest | IDD |
|  | Study Population and Sample Size | 40,000 Caregivers |
|  | Medical Condition(s) | ADHD |
|  | Study Purpose | To describe the influence of PCMH on economic burden reported by families of children with ADHD across the United States. |
|  | Population(s) of interest are focus of the research | Yes |
|  | PCBEOs are a focus of the research | Yes |
|  | Data Collection Geography | National |
|  | Data Source(s) | Primary survey data |
| Findings | PCBEO(s) Studied |  |
|  | Indirect impacts | Unpaid time off work, early retirement |
|  | Intangible burdens | Hindered career or education advancement, General or composite measure of caregiver burden, Financial problems, Avoiding changing jobs due to concerns about health insurance |
|  | Population-specific Justification Included | Yes |
|  | Justification(s) | Need factor(s): Chronic illnesses, Disability status |

| **Parish, S. L., Thomas, K. C., Williams, C. S., & Crossman, M. K. (2015). Autism and families’ financial burden: The association with health insurance coverage. *American Journal on Intellectual and Developmental Disabilities*, *120*(2), 166–175.** | | |
| --- | --- | --- |
| Study Characteristics | Population(s) of Interest | IDD |
|  | Study Population and Sample Size | 316 Caregivers |
|  | Medical Condition(s) | ASD |
|  | Study Purpose | To examine the relationship between family financial burden and children’s health insurance coverage in families raising children with autism spectrum disorders (ASD). |
|  | Population(s) of interest are focus of the research | Yes |
|  | PCBEOs are a focus of the research | Yes |
|  | Data Collection Geography | National |
|  | Data Source(s) | Secondary survey data |
| Findings | PCBEO(s) Studied |  |
|  | Direct medical costs | Copayments, coinsurance, deductibles, premiums |
|  | Population-specific Justification Included | Yes |
|  | Justification(s) | Need factor(s): Disability status |

| **Chatterji, P., Decker, S. L., & Markowitz, S. (2015). The effects of mandated health insurance benefits for autism on out-of-pocket costs and access to treatment. *Journal of Policy Analysis and Management*, *34*(2), 328–353.** | | |  |
| --- | --- | --- | --- |
| Study Characteristics | Population(s) of Interest | IDD | |
|  | Study Population and Sample Size | 53,631 Caregivers (19,475 IDD) | |
|  | Medical Condition(s) | ASD and Non-ASD developmental disorders | |
|  | Study Purpose | To explore whether state ASD mandates are associated with OOP costs and financial burden related to privately insured children’s treatment, and cost or insurance-related problems with access to treatment. | |
|  | Population(s) of interest are focus of the research | Yes | |
|  | PCBEOs are a focus of the research | Yes | |
|  | Data Collection Geography | National | |
|  | Data Source(s) | Secondary survey data | |
| Findings | PCBEO(s) Studied |  | |
|  | Direct medical costs | Copayments, coinsurance, deductibles, premiums | |
|  | Population-specific Justification Included | Yes | |
|  | Justification(s) | Gaps in the literature; Policy factors | |

# Supplement F: Types of PCBEOs justified by population (n=96 articles justified)

| PCBEO (n, % of justifications) | | Veteran | | Intellectual and developmental disabilities (IDD) | | Rural | |
| --- | --- | --- | --- | --- | --- | --- | --- |
|  |  | (n justified=13, % of veteran-specific justifications) | Relevant citations | (n justified=50, % of IDD-specific justifications) | Relevant citations | (n justified=35% of rural-specific justifications) | Relevant citations |
| **Direct medical costs (n=36, 38%)** | **Copayments, coinsurance, deductibles, premiums (n=20, 21%)** | 1 (8%) | (7) | 13 (26%) | (11, 47, 70-74, 76-81) | 6 (17%) | (51, 52, 54, 59, 83, 84) |
|  | **Diagnosis/treatment/management costs (beyond cost sharing) (n=10, 10%)** | 0 (0%) |  | 4 (8%) | (11, 47, 66, 72) | 6 (17%) | (51, 52, 54, 56, 59, 87) |
|  | **Medical transportation (n=1, 1%)** | 0 (0%) |  | 1 (2%) | (78) | 0 (0%) |  |
|  | **Out-of-pocket (OOP) medication costs (n=11, 11%)** | 0 (0%) |  | 5 (10%) | (47, 77-79, 88) | 6 (17%) | (51, 52, 54, 83, 89, 90) |
|  | **Personal medical equipment or consumables (n=4, 4%)** | 0 (0%) |  | 0 (0%) |  | 4 (11%) | (51, 56, 59, 90) |
|  | **Medical financial debt (n=1, 1%)** | 0 (0%) |  | 0 (0%) |  | 1 (3%) | (91) |
|  | **Other/general direct medical costs (n=9, 9%)** | 1 (8%) | (32) | 6 (12%) | (45, 49, 92-94, 98) | 2 (6%) | (55, 99) |
| **Direct non-medical costs (n=23, 24%)** | **Car or home modifications (n=0, 0%)** | 0 (0%) |  | 0 (0%) |  | 0 (0%) |  |
|  | **Child/dependent care costs (n=5, 5%)** | 1 (8%) | (39) | 3 (6%) | (65, 100, 101) | 1 (3%) | (102) |
|  | **Transportation, meals, hoteling (n=17, 18%)** | 0 (0%) |  | 8 (16%) | (70, 71, 73, 76, 77, 100, 103, 104) | 10 (29%) | (51, 53, 58, 59, 62, 84, 103, 105-107) |
|  | **Other/general direct non-medical costs (n=1, 1%)** | 0 (0%) |  | 1 (2%) | (92) | 0 (0%) |  |
| **Indirect Impacts (n=47, 49%)** | **Increased time required for daily tasks (n=1, 1%)** | 0 (0%) |  | 1 (2%) | (109) | 0 (0%) |  |
|  | **Time to attend medical appointments (n=6, 6%)** | 0 (0%) |  | 3 (6%) | (73, 76, 104) | 3 (9%) | (12, 53, 84) |
|  | **Time spent navigating insurance or billing (n=1, 1%)** | 0 (0%) |  | 1 (2%) | (109) | 0 (0%) |  |
|  | **Time spent providing unpaid caregiving (n=24, 25%)** | 4 (31%) | (7, 32, 35, 110) | 16 (32%) | (41, 42, 44-46, 48, 49, 65, 66, 77, 78, 94, 98, 101, 113, 115) | 4 (11%) | (57, 60, 63, 116) |
|  | **Time spent traveling to or between medical appointments (n=12, 13%)** | 1 (8%) | (7) | 3 (6%) | (103, 104, 109) | 9 (26%) | (12, 62, 83, 89, 102, 103, 105, 106, 119) |
|  | **Unpaid time off work, early retirement (n=21, 22%)** | 1 (8%) | (7) | 15 (30%) | (46, 48, 49, 65, 66, 77, 78, 88, 92, 98, 100, 104, 115, 122, 124) | 5 (14%) | (12, 53, 62, 84, 102) |
|  | **Other/general indirect impacts (n=5, 5%)** | 0 (0%) |  | 4 (8%) | (104, 122, 125, 126) | 2 (6%) | (125, 127) |
| **Intangible burdens (n=63, 66%)** | **Delaying or forgoing medical treatments due to costs (n=13, 14%)** | 1 (8%) | (38) | 4 (8%) | (10, 76, 125, 129) | 9 (26%) | (12, 54, 56, 59, 61, 106, 125, 131, 133) |
|  | **Diminished household wealth (n=4, 4%)** | 0 (0%) |  | 2 (4%) | (48, 65) | 2 (6%) | (87, 106) |
|  | **Family/social life impacts (n=3, 3%)** | 1 (8%) | (110) | 2 (4%) | (48, 92) | 0 (0%) |  |
|  | **Fear and anxiety about future ability to afford medical treatments (n=1, 1%)** | 0 (0%) |  | 1 (2%) | (65) | 0 (0%) |  |
|  | **Changes in employment status and/or insurance coverage (n=6, 6%)** | 0 (0%) |  | 3 (6%) | (94, 101, 125) | 4 (11%) | (12, 63, 106, 125) |
|  | **Food insecurity, housing insecurity (n=1, 1%)** | 0 (0%) |  | 0 (0%) |  | 1 (3%) | (106) |
|  | **Hindered career or education advancement (n=5, 5%)** | 1 (8%) | (32) | 4 (8%) | (65, 92, 115, 124) | 0 (0%) |  |
|  | **Quality-of-life impacts (not QALYs or DALYs) due to medical costs (n=1, 1%)** | 0 (0%) |  | 1 (2%) | (65) | 0 (0%) |  |
|  | **Reduced spending on basic goods, groceries, and leisure activities (n=3, 3%)** | 0 (0%) |  | 1 (2%) | (134) | 2 (6%) | (102, 106) |
|  | **Distress, stress, and anxiety directly caused by OOP costs, financial debt, or lost income (i.e., financial toxicity) (n=16, 17%)** | 2 (15%) | (35, 110) | 5 (10%) | (46, 48, 65, 101, 113) | 9 (26%) | (12, 53, 57, 60, 87, 99, 106, 116, 137) |
|  | **General or composite measure of caregiver burden (n=35, 36%)** | 10 (77%) | (7, 33-37, 110, 139, 141, 142) | 20 (40%) | (40-46, 48, 66, 109, 115, 124, 125, 146-152) | 6 (17%) | (50, 57, 102, 125, 127, 153) |
|  | **Other/general intangible burdens (n=9, 9%)** | 0 (0%) |  | 8 (16%) | (40-42, 49, 100, 115, 124, 125) | 2 (6%) | (12, 125) |

DALYs = disability-adjusted life-years; PCBEOs = patient-centered burdens and economic outcomes; QALYs = quality-adjusted life-years.

Note. Other/general direct medical costs include general financial burden, composite OOP costs, OOP costs for medical visits/care, spending per year on healthcare, and spending on child’s care. Other/general direct non-medical costs include moving costs to be closer to medical care. Other/general indirect impacts include disruptions to daily life/schedule, absenteeism and presenteeism, and school absenteeism. Other intangible burdens include general financial burden/problems, emotional burden, reduced self-care, and avoiding changing jobs due to concerns about health insurance. Examples of personal medical equipment or consumables include continuous positive airway pressure (CPAP) machine, syringes, gauze and bandages, glucometer, blood glucose test strips, and insulin pump.
